# ERAMOSA controls lateral branching in snapdragon

**DOI:** 10.1038/srep41319

**Published:** 2017-02-01

**Authors:** Chiara Mizzotti, Bianca M. Galliani, Ludovico Dreni, Hans Sommer, Aureliano Bombarely, Simona Masiero

**Affiliations:** 1 Department of Biosciences, Università degli Studi di Milano, via Celoria 26, 20133 Milan, Italy; 2 Max-Planck-Institut fuer Zuechtungsforschung, Carl-von-Linne-Weg 10, 50829 Koeln, Germany; 3 Department of Horticulture, Virginia Tech, Blacksburg, VA 24060, USA

## Abstract

Plant forms display a wide variety of architectures, depending on the number of lateral branches, internode elongation and phyllotaxy. These are in turn determined by the number, the position and the fate of the Axillary Meristems (AMs). Mutants that affect AM determination during the vegetative phase have been isolated in several model plants. Among these genes, the GRAS transcription factor *LATERAL SUPPRESSOR (Ls*) plays a pivotal role in AM determination during the vegetative phase. Hereby we characterize the phylogenetic orthologue of Ls in Antirrhinum, ERAMOSA (ERA). Our data supported ERA control of AM formation during both the vegetative and the reproductive phase in snapdragon. A phylogenetic analysis combined with an analysis of the synteny of Ls in several species strongly supported the hypothesis that ERA is a phylogenetic orthologue of Ls, although it plays a broader role. During the reproductive phase ERA promotes the establishment of the stem niche at the bract axis but, after the reproductive transition, it is antagonized by the MADS box transcription factor SQUAMOSA (SQUA). Surprisingly double mutant *era squa* plants display a *squa* phenotype developing axillary meristems, which can eventually turn into inflorescences or flowers.

The aerial plant body derives from the Shoot Apical Meristem (SAM), through the iterative production of phytomers. A phytomer unit is comprised of a node, to which a leaf is anchored, the corresponding internode and an Axillary Meristem (AM) at the leaf axil^1^. AM initiation occurs at the anlagen of the apical meristem, giving rise to new buds, again formed by a set of phytomers that can remain dormant or grow out^2^. Plants produce new AMs throughout their life cycle, but the nature and properties of AMs change during the different developmental phases. This becomes evident after the floral transition, since the committed floral meristems are indeed products of AMs and are homologous to vegetative axillary buds^3^.

Two theories have been proposed to explain AM formation. The ‘detached meristem’ theory proposes that the SAM gives rise to AMs during the production of leaf primordia^4^, therefore the AM founder cells remain undifferentiated. The ‘*de novo*’ model postulates that AMs are induced from previously differentiated cells in the leaf axis^5^.

In several plant species, mutants impaired in plant architecture have been described, suggesting a tight genetic control. They are grouped into three main classes: mutants affected in AM initiation, meristem outgrowth, or both^6^.

An interesting mutant altered in AM initiation is the tomato *lateral suppressor (ls*)^7^. Ls function is quite conserved, as indicated by its orthologues in *Arabidopsis thaliana* (LAS) and in *Oryza sativa* (MONOCULM1, MOC1)^7^,^8^,^9^. All these mutants fail to produce vegetative AMs. Compared to wild-type plants, tomato *ls* plants produce fewer flowers (without petals); similarly the rice *moc1* rachis forms fewer branches and spikelets^9^. Conversely, the Arabidopsis *las* plants do not develop lateral branches during vegetative growth, but inflorescences and flowers appear normal.

The *Antirrhinum majus eramosa* phenotype is caused by the disruption of the *Ls* orthologue *ERA. era* plants are unable to produce AMs during the vegetative phase, whilst during the reproductive phase, *era* inflorescences develop very few flowers. *ERA* overexpression in Arabidopsis stimulates branching, strengthening the important role of the Ls clade in controlling agronomic traits. Our phylogenetic reconstruction revealed that leguminous species lack Ls orthologues, suggesting that this family has developed a different means of controlling AM determination.

## Results and Discussion

### *ERA* is required for axillary meristem formation in *Antirrhinum*

**Figure 1:**
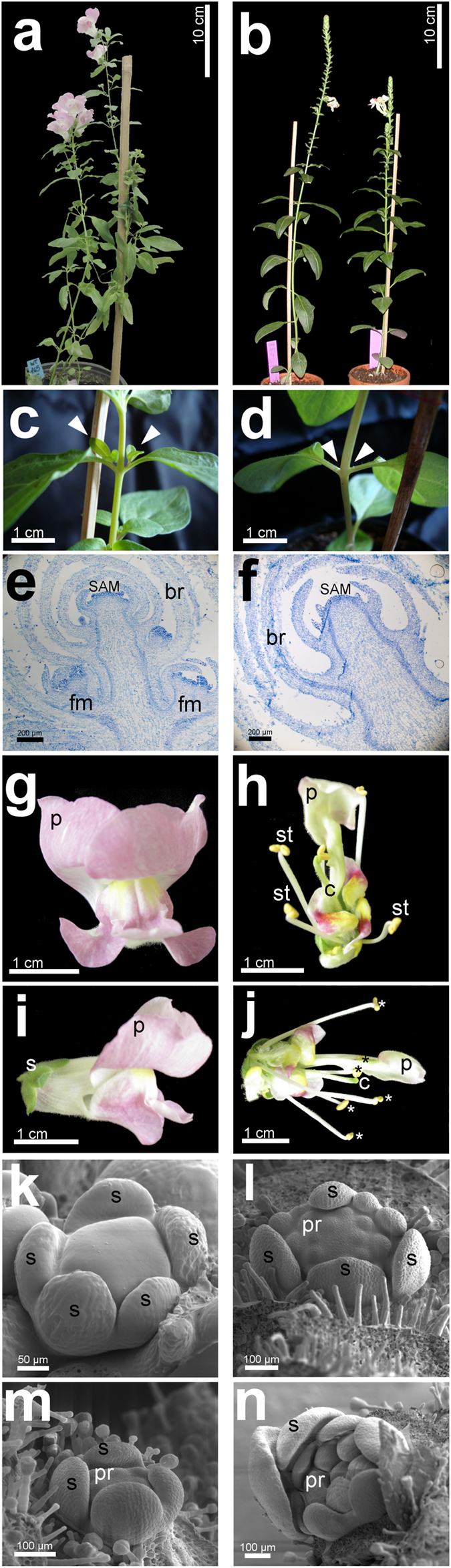
*Antirrhinum majus eramosa* phenotypes. (**a**–**d**) Branching of Antirrhinum plants. Wild-type plants possess a main stem and several lateral branches (**a**) while *era* plants have a single stem and no lateral branches (**b**). At the axil of wild-type leaves AMs form buds that give rise to new phytomers (**c**, arrowheads), while *era* leaf axils do not subtend new buds (**d**, arrowheads). (**e**,**f**) Histological sectioning of wild-type (**e**) and *era* SAM (**f**) stained with Toludine Blue O. At the axil of wild-type bracts developing buds are present (**e**) while the axil of *era* bracts is vacant (**f**). (**g**–**j**) Antirrhinum flower. Wild-type flowers contain five petals with a dorsoventral asymmetry and enclose the flower, hiding the stamens and carpel (**g**,**i**). In *era* flower stamens and carpel are visible and exposed (**h**,**j**); supernumerary organs are developed (6 stamens, asterisks). (**k**–**n**) SEM analysis of flower ontogenesis. In the first whorl of wild-type flowers, five sepal primordia are formed (**k**) and, later in development, in the second and third whorls, petal and stamen primordia are developed (**m**). In *era* flowers, several primordia at different developmental stages are formed in the first whorl (**l**). In the inner whorls supernumerary primordia are developed which are not well positioned and not synchronized (**n**). SAM = Shoot Apical Meristem; br: bracts; fm: floral meristem; s: sepals; p: petals; st: stamens; c: carpel.

Antirrhinum exhibits different types of phyllotaxy during its growth^10^. During the early vegetative phase, the leaves develop according a decussate phyllotaxy and AMs are formed at the leaf axis^11^. The reproductive phase is marked by a switch to a spiral phyllotaxy, during which single bracts develop at each node and subtend a new floral AM (Fig. 1a–d)^11^.

The *era* mutant^12^ resembles *ls/las* mutants. *era* plants do not develop AMs, therefore they have a single stem displaying a very erect growth without secondary branches and inflorescences (Fig. 1a–d). During the vegative phase, *era* plants develop at each node decussate leaves and after the transition to the reproductive phase bracts are produced according to a spiral phyllotaxis. Histological sections confirm the inability of *era* plants to produce AMs (Fig. 1e,f). Occasionally, a few abnormal flowers (from 1–2 to 5–6 per plant) are formed while the wild-type inflorescence is an indeterminate raceme (Fig. 1a,b,g–j and Supplementary Fig. S1). Flower ontogeny determined by Scanning Electron Microscopy (SEM) confirmed that *era* flowers present several defects, such as altered organ position and/or supernumerary organs (Fig. 1k–n). *era* inflorescences develop very few flowers, with unpredictable spatiotemporal distribution of AMS, and *era* flowers sometimes develop at the axis of quite well formed bracts.

### *era* and similar mutants in other species

The severity of the *era* phenotype implicates a pivotal role for this locus in controlling AM formation in either the vegetative or the reproductive phase. *ls* and *las* display less severe effects during the reproductive phase, therefore at least in *Solanaceae* and *Brassicaceae,* reproductive AMs might be controlled by parallel pathways or by redundant factors.

The morphological defects of the *ls* mutant are accompanied by changes in hormone levels: *ls* plants produce more auxin and gibberellic acid, and accumulate less cytokinin^13^. In addition, the level of abscisic acid (ABA) is affected. indeed tomato *ls* stems accumulate more ABA than wild type stems^14^. It has been demonstrated that higher levels of auxin and ABA inhibit bud development^14^. This inhibition, along with the converse relationship between hormonal content and apical dominance^15^,^16^ could explain the *ls* phenotype.

*era* decapitation causes plant death and grafting procedures fail, providing indirect evidence for hormonal defects in *era* plants since hormones like auxin and cytokinin are potential candidates involved in the grafting procedure^17^ and definitely auxin impairment is strictly related to phyllotaxis and apical dominance and it is clear that auxin depletion is strictly tied to phyllotaxis and apical dominance^18^,^19^.

### ERA is a GRAS protein and is the orthologue of LAS/Ls

The similarity recorded between *era* and *ls* raised the hypothesis that the *era* phenotype might result from a defective *Ls*-like gene. We unsuccessfully screened genomic and cDNA libraries using heterologous probes; similar difficulties were encountered isolating *Ls*^7^. By using degenerate primers designed with the conserved region of *LAS* and *Ls* (Supplementary Table S1) we were able to amplify the first *ERA* fragment. The complete genomic locus has been retrieved aligning the *ERA* sequence against the available sequence of the Antirrhinum genome (Xue Yongbiao, Beijing Institute of Genomics, unpublished). The *ERA* gene is 1239 bp long, intronless and encodes a GRAS protein of 412 aa with all the characteristic domains (Supplementary Fig. S2)^20^.

PCR analyses using *era* mutant genomic DNA, amplified with gene specific and generic transposon primers, identified the insertion of a CACTA transposon 561 bp downstream from the ATG. The transposon is a *TAM1***-**like^21^ and a deletion in one of the CACTA ends makes the transposon unable to transfer. We backcrossed *era* on E165 wild-type plants, and then analysed 124 F2 plants. The transposon insertion showed absolute cosegregation with the mutant phenotype (Chi-square test for a 1:2:1 segregation hypothesis = 0.387; P-Value = 0.824).

### *ERA* regulates meristematic tissue formation outside the SAM

**Figure 2:**
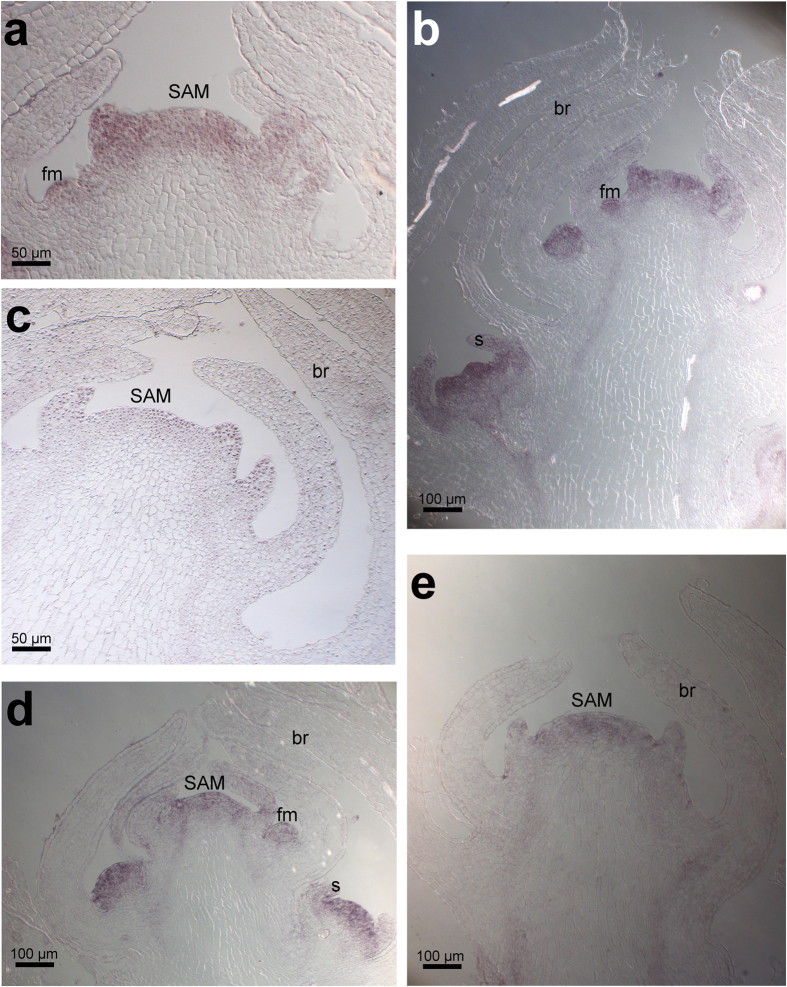
*ERA* and *STM* expression patterns analysed through *in situ* hybridizations. (**a**,**b**) *ERA* transcript in wild-type plants is detected in the SAM (**a**), in the floral meristems and in developing flowers (**b**). (**c**) In *era* mutant plants no transcript is detected. (**d**) In wild-type plants *STM* is expressed in the SAM and in developing floral meristems. (**e**) In *era* mutant plants no STM expression is detected outside the SAM. SAM = Shoot Apical Meristem; br: bracts; fm: floral meristem; s: sepals.

The distribution of *ERA* mRNA was analysed with semi-quantitative RT-PCR and mRNA *in situ* hybridization. Semi-quantitative RT-PCR showed that *ERA* is expressed in roots, stem, leaves, inflorescence and flowers at different developmental stages (Supplementary Fig. S3). *In situ* hybridizations showed *ERA* transcripts in the SAM, both in the epidermal and subepidermal layers, in the floral dome and sepal primordia (Fig. 2a,b)^22^. No *ERA* expression was detected in the *era* mutant (Fig. 2c). Unlike its orthologues, *ERA* expression is not restricted to the incipient AMs, as for *MOC1*, or at the adaxial side of the leaf primordium, as for *LAS*^8^,^9^. This broader expression could explain the severe *era* phenotype during the reproductive phase.

The inability of *era* mutants to produce new axillary meristems during the reproductive phase might be caused by the absence of meristematic tissue in the axil, or by the inability to produce new flower buds from the meristematic tissue. In order to discriminate between these hypotheses, we analysed the expression of *SHOOT MERISTEMLESS (STM*) in wild-type and *era* plants. In wild-type plants *STM* is expressed in the SAM and in floral meristem, but not in leaf initials, providing an early marker for AMs development (Fig. 2d)^23^. In *era* mutants, *STM* transcripts are detected only in the SAM and no *STM* expression is detected at the axil of new putative flowers (Fig. 2e). These data clearly indicate that no meristematic tissue is formed during the reproductive phase at the axil of *era* mutants, highlighting the role of ERA in the production of meristematic tissue outside the SAM. Similarly, the rice orthologue MOC1 is able to control the expression of *OSH1* in leaf axils while in Arabidopsis, LAS controls *STM* accumulation only in old leaf axils^8^. These data still suggest a fundamental role of ERA in AM formation, and are consistent with the idea that LAS/MOC1/ERA prevent the differentiation of cells in the boundary zone, thus stimulating meristem formation^1^.

### ERA and SQUAMOSA are epistatic after the floral transition

MADS box transcription factors are important regulators of vegetative development. Silencing of the *Paulownia kawakamii PkMADS1* stimulates leaf formation with altered phyllotaxy at the expense of meristem maintenance^24^, whilst potato MADS-box 1 (*POTM1*) maintains the growth equilibrium between axillary and apical vegetative meristems. POTM1 belongs to the APETALA1/SQUAMOSA (AP1/SQUA) clade^25^.

Arabidopsis AP1 participates in the establishment of the floral meristem^26^ by regulating cytokinin homeostasis. In *ap1* plants, perturbation in cytokinin content causes the formation of ectopic flowers at the axils of sepals. In Antirrhinum, *SQUA* encodes a MADS-box transcription factor that shares high sequence similarity with Arabidopsis *AP1*^10^, *SQUA* mainly regulates floral meristem identify and proliferation^27^,^28^.

**Figure 3:**
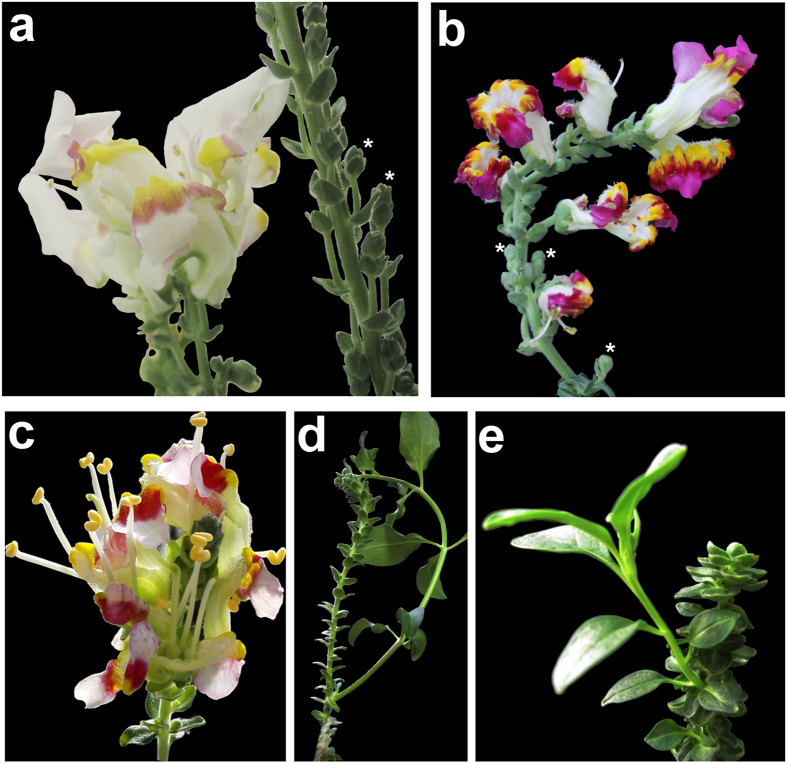
Double mutant *era squa.* (**a**) *squa* single mutant produces a reduced number of abnormal flowers, while the *era squa* double mutant plants produced more flowers than *squa* (**b**). (**c**) Intriguingly these flowers are similar to the *squa* mutant flowers and they appear much more regular than the *era* flowers. (**b**,**d**,**e**) At the axis of the *era squa* bracts, new primordia develop frequently producing either a flower, an inflorescence or a new seedling (**d**,**e**).

We therefore generated *era squa* double mutants to assay whether these two genes can interact to modulate axillary meristem formation during the reproductive phase. e*ra squa* plants resemble *era* in the vegetative phase, with no AM development at the axis of the decussate leaves. SQUA is epistatic to ERA after the floral transition, since new primordia develop frequently at the axis of bracts (Fig. 3b,d,e). These primordia can give rise to flowers, inflorescences or new seedlings (reversion, Fig. 3d,e). The reversion phenotype can be explained by decreased levels of *INCOMPOSITA* in the *squa* mutant^28^. INCO is homologous to tomato JOINTLESS (J), and it has been reported that *j* inflorescences revert to a vegetative shoot after the production of few flowers^29^.

Indeed it was clear that *squa* can recover *era* defects. Intriguingly, *era squa* plants produced more flowers than *squa* (Fig. 3a–c), which completely resembled *squa* flowers, and appeared much more regularly than those of *era* (Fig. 1h,j).

### ERA forms homodimers and heterodimers

**Figure 4:**
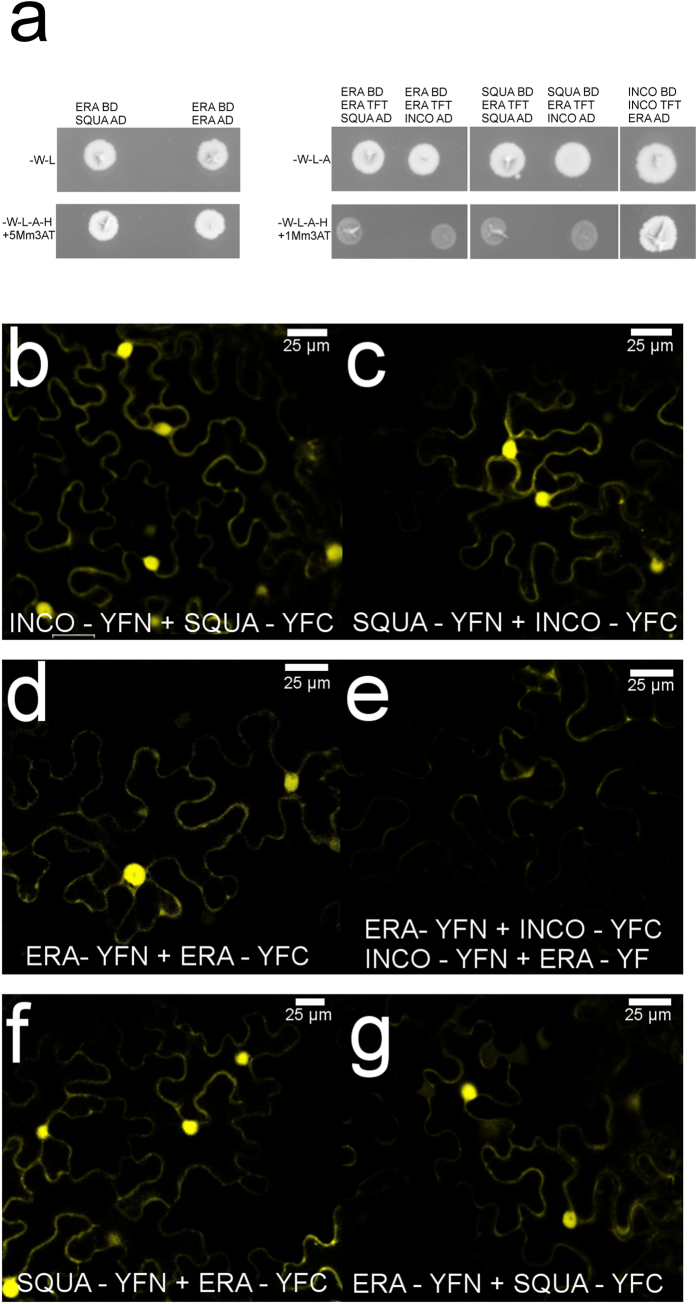
*In vivo* demonstration of the interaction among ERA, SQUA and INCO with yeast hybrid and BiFC assays. (**a**) yeast two and three hybrid (Y2H and Y3H) assays demonstrated that ERA physically interacts with SQUA and forms homodimers. The Y3H assay proved that the homodimer INCO-INCO can interact with ERA while all the other combinations tested fail to grow. (**b**,**c**) The interaction among INCO and SQUA was used as positive control for testing the interaction: the two proteins are able to interact *in planta* and to fully reconstruct the YFP signal. The reconstruction of the YFP signal was also observed when ERA interacted with itself (**d**), suggesting the formation of homodimers, and when ERA interacted with SQUA (**f**,**g**), suggesting the formation of heterodimers. No interaction was detected between ERA and INCO as suggested by lack of reconstitution of the YFP signal (**e**).

Since ERA and SQUA interact genetically, we asked whether ERA is also able to heterodimerize with SQUA and eventually with other MADS-box transcription factors involved in the establishment of the floral meristems, using yeast two and three hybrid (Y2H and Y3H) and Bimolecular Fluorescence Complementation (BiFC) assays. Y2H tests using ERA as bait demonstrated that this GRAS protein physically interacts with the MADS box transcription factor SQUA (Fig. 4a). Furthermore, the Y2H assays demonstrated that ERA is able to form homodimers (Fig. 4a). In order to reproduce *in vivo* the interaction among these proteins we exploited the BiFC technique, using leaves of *Nicotiana benthamiana* for the transient expression of protein chimeras (Fig. 4b–g)^30^. The results confirmed the data obtained through the Y2H assay, thus ERA is able to interact with SQUA, forming heterodimers, and with itself forming homodimers (Fig. 4d,f,g).

SQUA participates in the commitment of the Antirrhinum floral meristem together with the MADS-box INCO. Indeed, genetic and Y2H evidence indicates that the dimer INCO-SQUA promotes flower development^28^. Interestingly, INCO can homodimerise (although quite weakly) and the INCO-INCO homodimer represses flower development^28^. We therefore asked whether ERA could interact with INCO, since *era squa* inflorescences strongly resemble those of *inco era* plants. To this end, we set up an yeast ternary assay^28^,^31^, in which the INCO open reading frame was cloned into pGBKT7 and pTFT1 yeast expression vectors (see material and methods) and these plasmids were used to co-transform yeast strain AH109 with GAL4AD-ERA. The ternary assay scored positively, demonstrating the INCO-INCO dimer can interact with ERA (Fig. 4a) and participates in floral meristem outgrowth. In conclusion, ERA is able to interact with SQUA and with INCO but only when INCO is a homodimer, but the ternary complex ERA-SQUA-INCO was not detected. These data suggest that the interaction between ERA and INCO necessarily relies on INCO homodimer formation, providing a possible explanation for the lack of a ternary complex.

### Heterologous overexpression of *ERA* causes an increase in lateral branching but is not able to complement the *las* phenotype

To confirm the key role of *ERA* in AM determination, we overexpressed *ERA* in *A. thaliana* (Col-0) under the control of the constitutive promoter 35SCaMV. We analysed the potential shoot branching in the overexpressing lines, compared to wild-type, counting the number of AMs formed from the rosette leaves, without the removal of primary bolts to maintain the correct apical dominance. Half of the analysed lines produced more lateral branches (n = 10). The progeny of two lines with a single T-DNA insertion were used to count the number of lateral branches, confirming the data collected in T1 (Supplementary Fig. S3). Interestingly *MOC1* overexpression in rice also stimulates tiller proliferation^9^.

To understand if *ERA* is able to complement the *Arabidopsis thaliana las* phenotype we crossed the wild-type Columbia overexpressing plants with the las mutant. In the T2 generation, we counted the number of lateral branches both in WT, las and las 35S::AmERA (Supplementary Fig. S3). The *las* plants carrying the 35S::*AmERA* construct did not show an increase in the number of lateral branches respect to the *las* mutant. Two possible scenarios can explain this result. First, ERA is not able to complement *las*, possibly due to the fact that Arabidopsis and Antirrhinum are quite distantly related plant species and the genes could have acquired different functions. Second, and most probably, the regulatory pathways controlling the funtions of ERA and LAS are significanctly different and prevent complementation.

It is also possible that the 35S promoter is unable to express *ERA* in the founder AM cells. However, we observed that wild-type plants over- (and probably also mis-) expressing *ERA* produce more lateral branches; therefore we infer that the dimer ERA/LAS is sufficient to activate lateral branch formation, but not ERA alone.

### Phylogenetic reconstruction of the Ls subfamily

Previous screens, limited to Arabidopsis and rice, divided the GRAS transcription factor family into eight monophyletic subfamilies^32^. The Ls subfamily is further split into two main lineages: the first Ls lineage includes Ls, LAS, ERA, MOC1 and its close relative Os-GRAS7, whereas the second lineage includes Os-GRAS19, At-GRAS19 and At-GRAS32 proteins^33^. At-GRAS19 and At-GRAS32 are allelic to SCL7 (SCARECROW-LIKE7) and SCL4 respectively^20^. Therefore, we renamed these two lineages the Ls lineage and the SCL4 lineage respectively, from their founding member.

**Figure 5:**
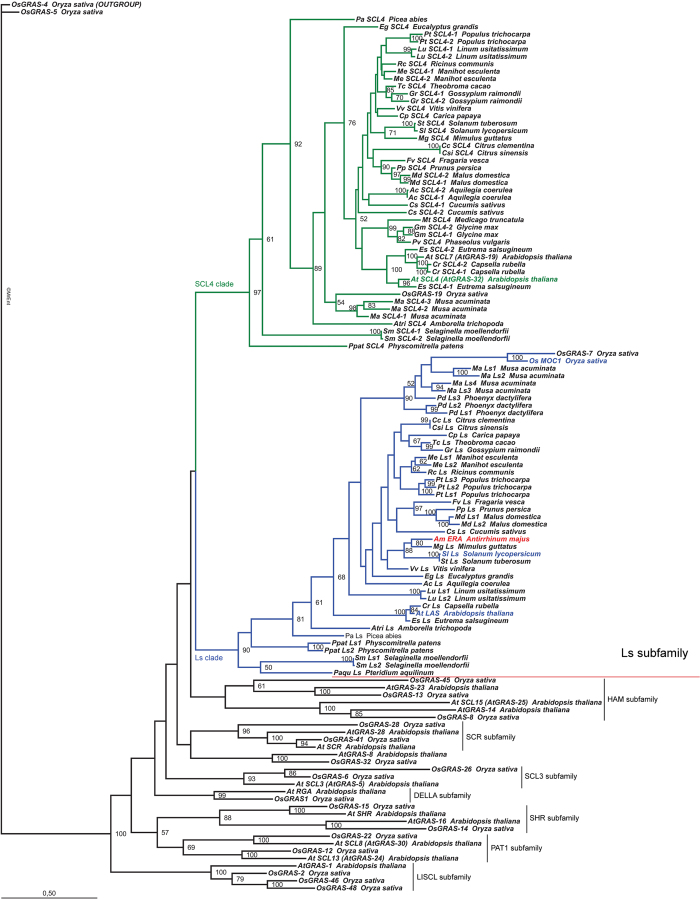
Maximum Likelihood phylogenetic reconstruction of Ls and SCL4 clades in land plants. Both clades appear as monophyletic and conserved in all of the taxa analyzed, without conserved gene duplication events, which seem mostly species-specific. However, Ls orthologues are missing in all of the genomes available from legumes. The scale bar indicates the number of amino acid changes per site.

The Ls subfamily originated before the divergence of mosses and vascular plants^34^. To understand the evolution of the Ls subfamily during land plant diversification, we screened several plant genomes and EST collections (Supplementary Table S2). Protein alignment analyses allowed us to define the Ls subfamily throughout the plant kingdom (Supplementary Fig. S4). Either Ls or SCL4 orthologues are present in the moss *Physcomitrella patens*, as well as orthologues from other GRAS subfamilies (Supplementary Fig. S4)^34^. Therefore, the Ls and SCL4 clades diverged before the last common ancestor (LCA) of mosses and Tracheophyta. Our analysis revealed that both the clades are highly conserved within land plants (Fig. 5). Our analysis suggests that SCL4 lineage is sister to Ls, in agreement with previous studies conducted on a few angiosperm species^33^,^35^, which is worthy of further investigation.

Blast analysis failed to retrieve GRAS-like proteins from the available green algae genomes (Phytozome). In agreement with Engstrom’s conclusions^34^, our reconstruction suggests the GRAS family probably arose within the Streptophyta and soon diverged in land plant ancestors.

Within the Ls lineage, most eudicot species possess one *Ls/ERA* gene, although two *Ls-*like genes are found in species with recent whole genome duplication events such as *Linum usitatissimum, Manihot esculenta* and *Malus domestica*, and there are three *Ls-*like genes in poplar (Supplementary Fig. S4). The average number of Ls-like factors in monocots is higher: There are two copies in rice, four in *Musa acuminata* and at least three in *Phoenyx dactylifera* (Supplementary Fig. S4)^36^,^37^.

Surprisingly, no Ls/ERA orthologues were found in *Medicago truncatula, Phaseolus vulgaris* or *Glycine max*. We therefore screened the genomes of the other eight legume species currently available. Again, we could not retrieve any Ls-related factors. A new phylogenetic tree has been generated with all the ERAMOSA orthologues currently available (Supplementary Fig. S5), confirming that no ERA homologues can be found in the Leguminosae family. In conclusion, the Ls lineage has been lost before the LCA of Papilionoideae, the largest subfamily of Fabaceae. Therefore the molecular mechanism regulating branching is somehow modified in legumes, and apparently an Ls-like transcription factor is not required. However, it cannot be excluded that other GRAS transcription factors might assume this role.

### Micro-synteny of the Ls clade

**Figure 6:**
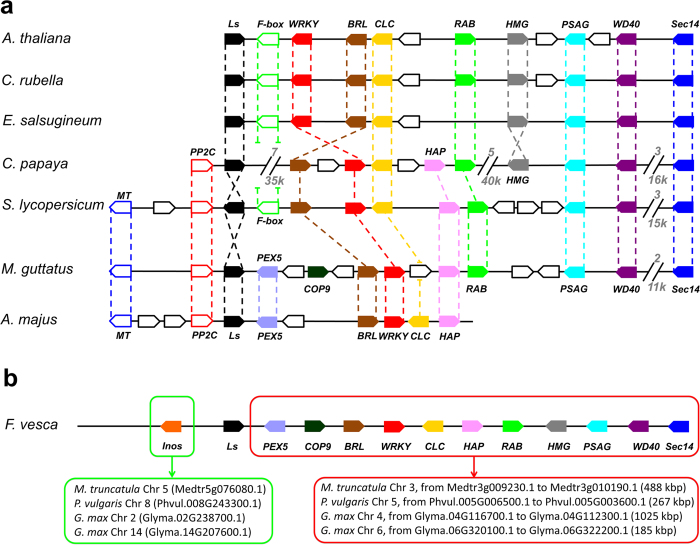
Micro-synteny of the Ls-like loci in core eudicots. (**a**) The figure shows the synteny downstream of the Ls homologous locus (represented in black). Genes which are poorly or not syntenic are indicated by white boxes with black margin and, where numerous, gaps have been inserted. In each gap, the number above is relative to the hidden known *loci*, and the number below is the approximative length of the gap, in kilobase pairs. For detail description, see materials and methods. (**b**) Schematic representation of the 256 kbp long corresponding region in the woodland strawberry (*Fragaria vesca*) chromosome LG3. A highly similar, 250 kbp long region is found in the chromosome 6 of peach (not shown). On the genome assembly, the *Ls* homolog is named mrna01184.1-v1.0-hybrid. Upstream, a gene which is conserved in sequence in core eudicots is encoding for an inosine-5-monophosphate dehydrogenase related protein, indicated in orange (*locus* gene01181-v1.0-hybrid). Downstream, the syntenic region is formed by the same genes found in (**a**) and in most other core eudicots, terminating with the gene encoding for the putative Sec14p-like phosphatidylinositol transfer protein (mrna01444.1-v1.0-hybrid). The scheme shows the corresponding genes and regions in the genomes of the legumes *Medicago truncatula, Phaseolus vulgaris* and the ancient tetraploid *Glycine max*. Any *Ls*-like gene or partial sequence is found within 1 Mbp upstream and 1 Mbp downstream from each of these regions, which was further confirmed by NCBI translated nucleotide blast tool (tblastn) using as a query the woodland strawberry Ls homolog protein sequence. In both (**a**) and (**b**), the length of the genes and of the intergenic spaces is not to scale. In (**b**), due to their high number and variability, non-syntenic genes are omitted. The analysis has been done by manual check of the genome browsers available in the Phytozome database.

In the phylogenetic trees the Brassicaceae Ls-like factors are separated from the Ls orthologues of other eudicots, although the eudicot clade includes the homolog from *Carica papaya*, which belongs to the order Brassicales (Fig. 5). However, the hypothesis that two paralog lineages were differentially lost and retained in Brassicaceae and in all other eudicots seems extremely unlikely. Genome synteny can be used to unambiguously identify orthologous genes in plants^38^. The chromosome regions downstream of the *Ls* genes of Arabidopsis and *Capsella rubella* display conservation in the gene order^39^; according to our analyses, synteny is also conserved in *Eutrema salsugineum,* papaya, mimulus, tomato and grape (Fig. 6). To a variable extent, the downstream synteny is conserved in many other core eudicots, or towards the upstream region in the case of *Vitis vinifera*, but not in the basal eudicot *A. coerulea* (data not shown). Altogether, the synteny reconstructions confirm that LAS is the true orthologue of Ls and ERA. Thus, the independent clustering of Brassicaceae Ls proteins seems only due to a higher degree of sequence divergence. We extended synteny reconstruction to legumes, and we have been able to identify the “ERA downstream block”, further supporting the absence of the *ERA/Ls* locus in this family (Fig. 6).

In conclusion, we identify the phylogenetic snapdragon orthologue of LAS/Ls/MOC1. Altogether our data further support the detached theory, suggesting that axillary meristem founder cells remain undifferentiated^4^,^5^,^40^.

## Material and Methods

### Plant material and growth conditions

The mutant plant *era* was originally described by Stubbe^12^, and the corresponding seeds were obtained from the Gatersleben Centre. *Antirrhinum majus* wild-type (ecotype E165) and *era* mutant plants were grown at 22 °C under long-day (16 h light/8 h dark) conditions.

### Analytical microscopy: histological analysis, SEM and *in situ* hybridization

For morphological analysis, wild-type (ecotype E165) and *era* inflorescences and seedlings were fixed as previously described^41^. Sections (8 μm) were stained in 0.5% (w/v) toluidine blue O or hybridized with digoxigenin-labelled *ERA* and *STM* antisense probes (primers are reported in Supplementary Table S1). DIG-labelled RNA probes were prepared as previously described^41^. The samples were observed using a Zeiss Axiophot D1 microscope equipped with differential interface contrast (DIC) optics. Images were recorded with an Axiocam MRc5 camera (Zeiss) using the Axiovision program (version 4.1). Scanning electron microscopy (SEM) was performed by producing replicas of flowers and developing inflorescences as described by Green and Linstead^42^. The samples were observed using a Zeiss DSM 940 scanning electron microscope.

### cDNA isolation

In order to isolate the *ERA* cDNA we screened a cDNA and a genomic DNA Antirrhinum library with probes generated by amplifying the conserved region of *LAS* and *Ls*. Subsequently, the *ERA* cDNA was amplified using degenerate primers to the conserved region of *LAS* and *Ls* (Supplementary Table S1). The *ERA* CDS and protein sequences from this publication have been submitted to the GenBank database (http://www.ncbi.nlm.nih.gov/genbank/) and assigned the identifier KT698108.

### Expression Analysis

To analyze *ERA* expression, semi-quantitative RT-PCR was performed in duplicate on cDNA obtained from two different biological replicates of RNA from different tissues. Total RNA was extracted using the LiCl method^43^. DNA contamination was removed using the Ambion TURBO DNA-free DNase kit according to the manufacturer’s instructions (Life Technologies). The RNA was reverse transcribed using the ImProm-II reverse transcription system (Promega) and the cDNA was used as template in semi-quantitative RT-PCR reactions. As a control, we simultaneously amplified an ACTIN fragment. Primers used for semi-quantitative RT-PCR are reported in Supplementary Table S1.

### Yeast two and three hybrids and BiFC assays

To determine the ability of ERA to interact with other partners, the coding sequences of ERA, INCO and SQUA were cloned into pDONR207 (Life Technologies) and subsequently by Gateway recombination transferred to the GAL4 system (pGADT7 and pGBKT7; Clontech) or pTFT1 Gateway (kindly provided by Marcos Egea Cortines; ref. 44). The two- and three-hybrid assays were performed at 28 °C in the yeast strain AH109 (Clontech) and were tested on selective yeast synthetic dropout medium lacking leucine, tryptophan, adenine and histidine and supplemented with different concentrations of 3-aminotriazole (1, 2 and 5 mM of 3-AT). For BiFC assay, the coding sequences of ERA, INCO and SQUA cloned into pDONR207 (Life Technologies) were transferred by Gateway recombination to the pYFPN43 and pYFPC43 vectors. BiFC was performed as previously described by Belda-Palazon *et al*.^30^ using in the co-infiltration procedure the p19 protein of the tomato bushy stunt virus to suppress gene silencing. The abaxial surfaces of infiltrated tobacco leaves were analyzed 5 days after inoculation. Primers used for gene cloning are reported in Supplementary Table S1.

### Plasmid Construction and Plant Transformation

To overexpress *ERA* in Arabidopsis plants, the entire *ERA* gene was amplified with primers containing attB1 and attB2 recombination sequences (Supplementary Table S1). *ERA* cDNA was then cloned into the pB2GW7 binary vector, under the regulation of the CaMV 35S constitutive promoter. The vector was introduced into *Agrobacterium tumefaciens* by electroporation and used to transform Arabidopsis wild-type Col-0 plants using the floral dip method^45^. Transformants were selected through BASTA selection. The number of AMs of transformed and control plants was counted to verify the role of ERA in AM formation. RNA from overexpressing lines was extracted from seedlings using the NucleoSpin RNA Plant kit (Macherey-Nagel). The RNA was reverse transcribed as above and the cDNA was used as template in semi-quantitative RT-PCR reactions. The semi-quantitative RT-PCR assay was conducted in triplicate. As control, we simultaneously amplified an ACTIN fragment. Primers used for semi-quantitative RT-PCR are reported in Supplementary Table S1.

### Phylogenetic analysis

Protein sequences from ERA, known ERA homologues, and the SCL4 clade, were used to conduct tblastn and blastp analyses to screen the plant sequences available at Phytozome (www.phytozome.net) and NCBI Genomes (https://www.ncbi.nlm.nih.gov/genome/). Homologues of *Arabidopsis lyrata, Brassica rapa* and of grasses, with the exception of *O. sativa*, were not included in the final phylogenetic analysis. *Picea abies*^46^ (www.congenie.org), *Amborella trichopoda* (www.amborella.org), *Musa acuminata*^47^ (http://banana-genome.cirad.fr/) and *Phoenyx dactylifera*^36^,^37^ were included. We also added a cDNA fragment of the *Pteridium aquilinum* LAS^48^. In *Ricinus communis* (www.phytozome.net) synteny reconstruction allowed identification of an ERA homologue (locus 28966.m000535). To confirm the absence of ERA and Ls homologues in the Leguminosae family, we extended blast analysis to the legume genomes currently available at WGS Sequencing Projects (NCBI) and Kazusa DB, which includes two independent genome sequences of *Cicer arietinum*^49^,^50^, *Lotus japonicus*^51^, *Cajanus cajan*^52^, *Lupinus angustifolius*^53^, *Vigna angularis*^54^, *Arachis duranensis*^55^, *Arachis ipaensis*^55^, *Glycine max*^56^, *Medicago truncatula*^57^, *Phaseolus vulgaris*^58^, *Trifolium pretense*^59^, and*Vigna radiate*^60^.

The best blast hits were then aligned using MAFFT (http://mafft.cbrc.jp/alignment/server/). The protein alignments were manually corrected and used to generate phylogenetic trees with the Maximum Likelihood methods with Phylip Package^61^. The Maximum Likelihood tree has been prepared with the MrEnt software^62^. We also included in the analysis members of the other GRAS subfamilies reported by Tian *et al*.^33^. This allowed us to discard blast hits belonging to different GRAS subfamilies, and to build the Ls subfamily trees presented in this work.

The phylogenetic tree reported in Supplementary Fig. S5 has been constructed using a Maximum Likelihood method through the PhyML software (JTT protein model, bootstrapping of 100). The optimal protein model was previously analyzed using ProTest. Protein datasets were downloaded from Phytozome, GenBank genomes, Sol Genomic Networks, Cucubits Genomic Database and the Kazusa Genomic Data FTP site.

The analysis of synteny between genomes has been conducted manually between the three Brassicaceae species *Arabidopsis thaliana* (49 kbp of chromosome 1), *Capsella rubella* (46 kbp of scaffold 1) and *Eutrema salsugineum* (46 kbp of scaffold 7), the related papaya (Caricaceae, Brassicales; 178 kbp of supercontig 12) and the three asterids: *Solanum lycopersicum* (Solanaceae, 143 kbp of chromosome 7), *Mimulus guttatus* (Phrymaceae, 110 kbp of scaffold 13) and *Antirrhinum majus* (Plantaginaceae, limited to the available sequence of contig 909). The conserved *loci* considered in the analysis encode the following: a putative ribosomal protein L11 methyltransferase (MT, white boxes with blue margin), a protein phosphatase 2C (PP2C, white boxes with red margin), a F-box and LRR interaction motif protein (F-box,. white boxes with green margin), a WRKY transcription factor (red), a putative leucine-rich receptor serine/threonine-protein kinase, BRASSINOSTEROID INSENSITIVE1-like (BRL, in brown), a CLC chloride channel (yellow), a protein similar to prenylated RAB acceptor 1 (green), a putative HMG-box and ARID/BRIGHT domain DNA-binding protein (grey), a photosystem I subunit G (PSAG, in light blue), a transducin/WD40 domain-containing protein (violet), a putative Sec14p-like phosphatidylinositol transfer protein (blue), a HAPLESS8 protein (HAP, in pink), a peroxisomal targeting signal 1 receptor PEX5 (in lavender color), and a COP9 signalosome subunit 6 (in dark green).

## Additional Information

**How to cite this article**: Mizzotti, C. *et al*. ERAMOSA controls lateral branching in snapdragon. *Sci. Rep.* **7**, 41319; doi: 10.1038/srep41319 (2017).

**Publisher's note:** Springer Nature remains neutral with regard to jurisdictional claims in published maps and institutional affiliations.

## Supplementary Material

Supplementary Figures and Tables
