# Supplementary material for: ERAMOSA controls lateral branching in snapdragon
Source: Sci Rep. 2017 Feb 1;7:41319. doi: 10.1038/srep41319 (PMC5286501; doi:10.1038/srep41319)
Supplement: Supplementary Figures and Tables [file srep41319-s1.pdf]

SUPPLEMENTARY INFORMATION

ERAMOSA controls lateral branching in snapdragon

Chiara Mizzotti, Bianca M. Galliani, Ludovico Dreni, Hans Sommer, Aureliano Bombarely and Simona Masiero

Figure S1. Antirrhinum wild-type inflorescence is an indeterminate raceme

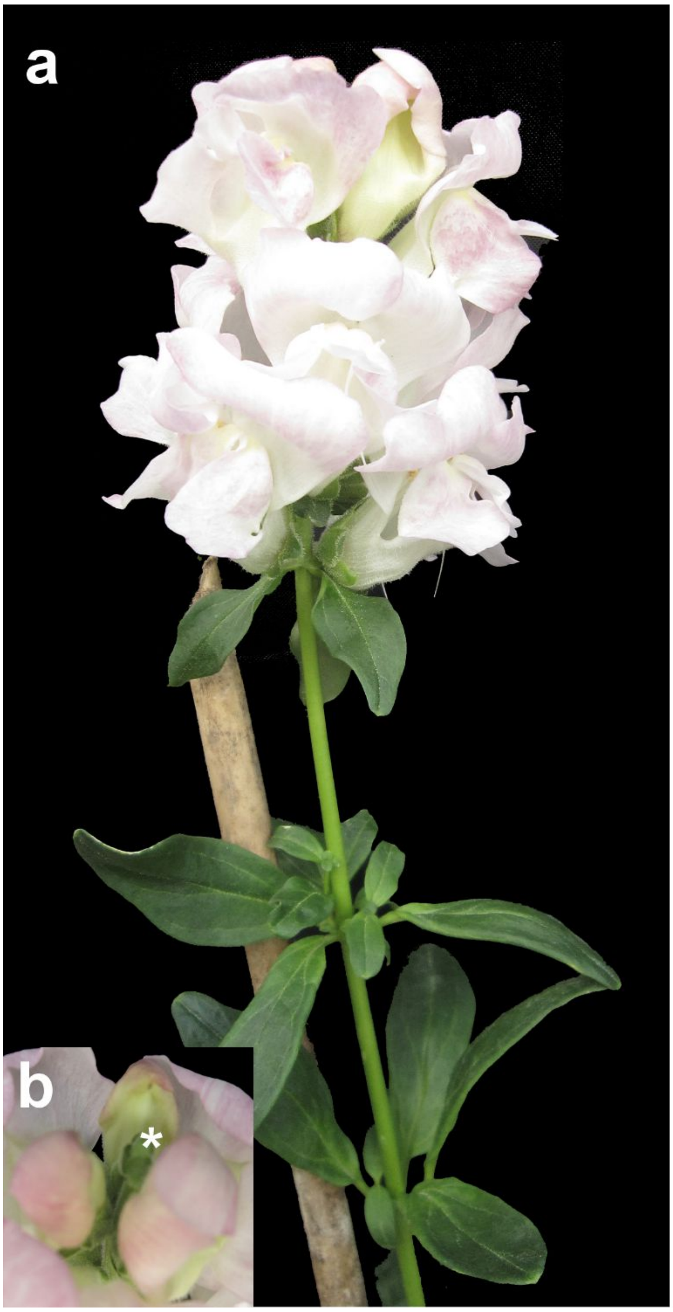

(**a**) The Antirrhinum wild type inflorescence is an indeterminate raceme.

(**b**) The flowers are disposed according to a spiral phyllotaxy starting from the SAM (asterisk).

Figure S2. *ERAMOSA* sequence.

```
 M  L  G  S  S  F  G  S  S  S  T  P  H  E  E  E  E  N  N  P
ATGTTGGGCAGCTCATTTGGTTCATCATCAACACCTCATGAAGAAGAGGAAAATAATCCC

 S  P  P  N  F  Y  D  L  H  H  H  H  H  H  H  Q  S  F  A  S
TCACCACCAAATTTCTACGATCTCCACCACCACCACCACCACCACCAGAGCTTCGCCTCT

 P  P  I  H  M  R  Q  L  L  I  S  C  A  E  L  V  A  R  S  D
CCACCAATCCACATGCGCCAGCTCCTCATCAGCTGTGCCGAGCTCGTTGCACGTTCCGAT

 Y  S  A  A  H  R  L  I  A  I  L  S  S  N  A  S  P  F  G  D
TACTCTGCCGCACACCGTCTCATCGCCATCCTCTCCTCAAACGCCTCGCCCTTCGGAGAC

 S  T  E  R  L  V  H  Q  F  T  K  A  L  T  L  R  L  N  R  A
TCCACCGAGAGACTAGTCCACCAATTCACCAAAGCCCTAACCCTCCGCCTCAACCGTGCC

 S  T  A  N  T  T  S  F  M  I  P  I  I  N  P  N  T  T  T  S
TCCACCGCAAACACTACTTCCTTCATGATCCCCATAATCAATCCCAACACCACCACGAGT

 A  N  E  E  A  L  L  Q  S  S  Y  L  S  L  N  Q  V  T  P  F
GCTAATGAAGAAGCCCTACTTCAGTCCTCATACTTGTCACTAAACCAAGTCACACCCTTC

 I  R  F  S  Q  L  T  A  N  Q  A  I  L  E  A  I  D  G  Q  Q
ATTAGGTTTAGCCAGCTCACCGCCAACCAAGCGATACTCGAAGCGATCGACGGCCAACAA

 A  I  H  I  L  D  F  D  I  M  H  G  V  Q  W  P  P  L  M  Q
GCCATCCACATACTCGATTTCGACATCATGCACGGCGTGCAATGGCCGCCGCTAATGCAG

 A  V  A  E  R  F  P  P  P  T  L  R  I  T  G  T  G  K  D  I
GCGGTAGCCGAGCGGTTCCCTCCCCCTACCCTCCGCATAACCGGCACTGGCAAAGACATT

 E  I  L  R  K  T  G  D  R  L  T  K  F  A  H  S  L  G  L  R
GAAATCCTTCGAAAAACCGGAGACCGTCTAACCAAATTTGCCCATTCGTTAGGCCTAAGG

 F  Q  F  H  P  I  T  N  Q  D  P  V  S  I  T  S  S  V  I  L
TTCCAATTCCACCCCATAACCAACCAAGACCCCGTCTCGATAACCTCCTCCGTTATCCTC

 L  P  D  E  T  L  A  V  N  C  V  H  Y  L  H  H  L  L  N  D
CTCCCCGACGAAACCCTAGCCGTAAACTGCGTGCATTATCTCCATCACTTGTTAAACGAC

 R  D  R  L  R  L  F  L  H  R  I  K  A  M  N  P  R  V  F  T
CGAGATCGCCTCCGGTTGTTCCTACACCGCATTAAGGCAATGAATCCTAGGGTTTTCACG

 V  A  E  R  E  A  N  H  N  H  P  L  F  Q  Q  R  F  L  E  A
GTCGCGGAGAGGGAAGCAAACCACAACCATCCCTTGTTTCAGCAGCGGTTCCTCGAGGCG

 L  D  H  Y  A  A  V  F  D  S  L  E  A  T  L  P  P  N  S  R
CTTGATCATTACGCGGCGGTGTTCGACTCGTTGGAGGCTACGTTACCGCCTAATAGCCGA

 E  R  M  A  V  E  Q  I  W  F  G  R  E  I  A  D  I  V  A  N
GAGCGGATGGCGGTGGAGCAGATATGGTTTGGGAGAGAAATAGCGGATATTGTGGCGAAT

 E  G  E  N  R  R  E  R  H  E  R  F  R  S  W  E  L  V  L  R
GAGGGTGAGAATAGAAGAGAAAGGCATGAGAGGTTTAGATCATGGGAGTTGGTTTTAAGA

 N  S  G  F  G  N  V  P  L  S  T  F  A  L  S  Q  A  K  L  L
AATTCGGGTTTTGGGAATGTTCCTTTGAGTACTTTTGCACTTTCGCAAGCTAAGCTTTTG

 L  R  L  H  Y  P  S  E  G  Y  Q  L  H  V  L  Q  D  S  C  F
TTGAGGCTTCATTATCCTTCTGAAGGGTATCAGCTTCATGTTCTTCAAGATTCTTGCTTT

 L  G  W  Q  N  Q  P  L  F  S  V  S  S  W  H  *
TTGGGATGGCAAAATCAACCCCTTTTCTCGGTTTCTTCGTGGCACTGA
```

Nucleotide and protein sequence of *ERA* gene. Degenerate primers used to identify the first *ERA* fragment are underlined. Black triangle indicates the insertion point of the CACTA transposon in the *era* mutant.

Figure S3. ***ERAMOSA*** **expression pattern and its overexpression in** ***Arabidopsis thaliana*****.**

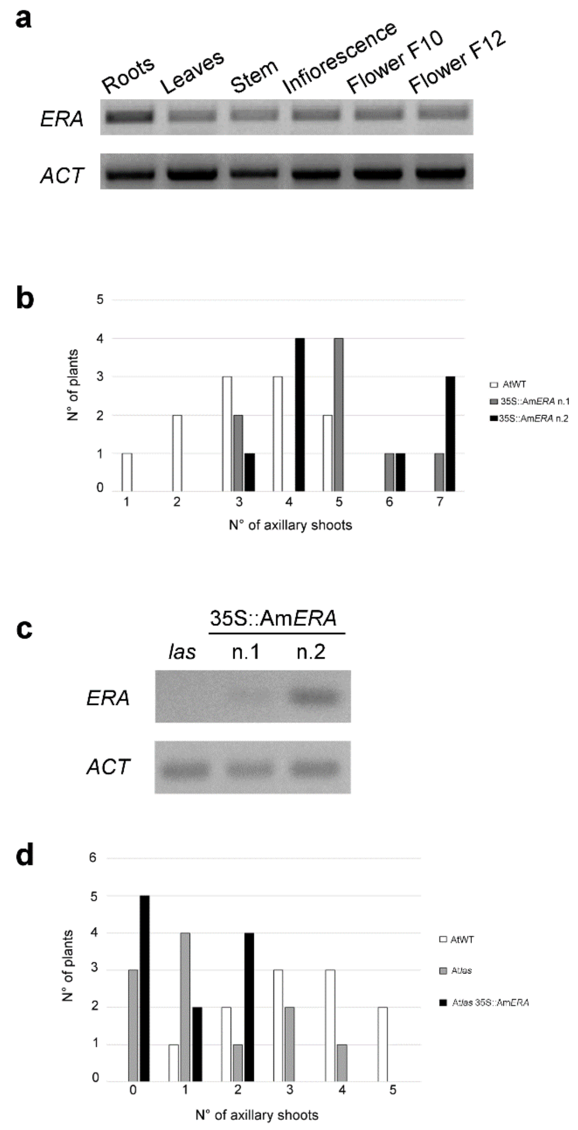

(**a**) The expression of *ERA* transcript analysed by semi-quantitative RT-PCR in duplicate using cDNA obtained from two different biological replicates of RNA (one of two identical results is shown). *ERA* is expressed in roots, leaves, stems and flowers at different stages of development (stages defined according to Vincent and Coen, 2004). As a control we amplified a fragment of an *ACTIN* gene.

(**b**) *ERA* overexpression in *Arabidopsis thaliana* Col-0 causes an increase in the number of secondary inflorescences formed at the axil of rosette leaves.

(**c**) The increase in lateral branching correlates with the overexpression of *ERA* transcript.

(**d**) *ERAMOSA* overexpression in a *las* background is not enough to complement the *las* phenotype.

Figure S4. Multi-fasta file of protein sequences used for the phylogenetic analysis.

```
>OsGRAS-4 .
MRLVQLLVACAEAVACRD-----RA-QAAALLRE---L-QA----GAP-----VHGT--------A-FQRVASCFVQG-LADRLPL-AHPP-AL--
GPASMAF--------------------------CIPPSSCAGR------------DGARGEALALA-----YELCPYLRFAHFVANACMLEAFE------
-GESN--------------VHVVDLG-MTLG-LDRGHQWRGLLDGLAARA--------SGK------PA-------------RVRVTGV-----------
----------GAR-MDTMRAIGRELEAYAEGLGMY-LEFRG-INR-GLE----------------------------SL-------HIDD-------
LGV--DAD-----EAVAINSVLELHSVV-----------------------------------------------------KESRG-------
ALNSVLQTIRKLSPRAFVLVEQDA-----------GH-N-GPFFLGRFM-------EALHYYAALFDAL-DA-A----LPRYDAR------
RARVEQFHFGAEIRNVV-GC----EGA--A-----R----VE-RHERADQ----WRRRMSR-AGFQSVPIK--MAAKAREWLDENAGGGG-------
YTVAEE--------KG----------CLVLGWKGKPVIAASCW
>OsGRAS-5 .
RRWRRAKTACRR---------------------------QR----------CRPARR--------A-FQRVASFFVQELLDRRASAGAGP----------
-------------------------------------------------------------------------------------------------
CEHG-------------VLH----------------------------------------------PA------------VVVR----------------
------------------------EAYAEGLGMY-LEFRG-INR-GLE----------------------------SL-------HIDD-------LGV-
-DAD-----EAVAINSVLELHSVV-----------------------------------------------------KESRG-------
ALNSVLQTIRKLSPRAFVLVEQDA-----------GH-N-APFFLGRFM-------EALHYYAALFDAL-DA-A----LPRYDAR------
RARVEQFHFGAEIRNVV-GC----EGA--A-----R----VE-RHERADQ----WRRRMSR-AGFQSVPIK--MAAKAREWLDENAGGDG-------
YTVADEKV------------------CLVLGWKGKPVIAASCW
>PaquLs .
-------------------------------------------------------------------MERVVTHFVDS-LAARVVQ--------------
--------------------------LXETQAASQLTAEQWLP--------HNLTDTELQGA-YLSLNQVTPFIRFTHLTANQAILEALQ-------
EWDA--------------VHIVDME-IMQG-----VQWPPLMQALAGRA--------GGP------P--------------KIRISGV------------
---------GSA-LSLLEQTGNRLTTFAASLGLPMFEFHP------------------------------------------------------------
-------------------LHAAK----------------------------------------------------------------------------
---------------------------N------------------------------------------------------------------DVV---
---------E------------------------------------------------------------------------------------------
--------------------
>SmLs1 .
--LRELLVECAAAVTSSD-----WH-RAIRCLVH---L-SR----AAS-----PHGD--------A-VERLAFYFSAA-LARCLCS-L------------
----SA-PCASEIRSLLRLNNL-HFLLEEDQPPPSFEDDLFYY------------SGGAEEA-YLALNQVTPFIRFSHLSANQAILEAVD-------
NERA--------------VHIVDLG-IMQG-----LQWPPLMQAL---------------------PPTLSSSSSSSSTTLTLRITGT------------
---------GPS-ISLLEQTGARLRDFARTLHLD-FEFDA-VCT-TSR----------------------------HV-VA-------S-LQ--QHLEL-
-RRG-----EALVVNCMTQLHKLL----------------------------------------------------PAAHRA-------
ALPHALEFMRSLCPRILTVAEKES-----------EH-DLSQSFLERFL-------VTLDHYAAVFDSL-EA-T----LPPRSPQ------
RLMIERLVLAKEISGIV-LE----DGG--GDDENLAVV-----RHQSFGN----WRRDMEA-AGFQLVPPSDFAIAQAKLLLRLHYPADG-------
YRL-----LVENQ-HG----------SLFLSWHDKPLVALSTW
>SmLs2 .
--LRELLVECAASVTSSD-----WH-RAIRCLVH---L-SR----AAS-----PHGD--------A-VERLAFYFSAA-LARCLCS-L------------
----ST-PCASEIRSLLRLNNL-HFLLEEDQPPPSFEDDLFYY------------SGGAEEA-YLALNQVTPFIRFSHLSANQAILEAVD-------
NERA--------------VHIVDLG-IMQG-----LQWPPLMQAL---------------------PPTLSSSSSSSSTTLTLRITGT------------
---------GPS-ISLLEQTGARLRDFARTLHLD-FEFDA-VCT-TSR----------------------------HV-VA-------S-LQ--QHLEL-
-RRG-----EALVVNCMTQLHKLL----------------------------------------------------PAAHRA-------
ALPHALEFMRSLCPRILTVAEKES-----------EH-DLSQSFLERFL-------VTLDHYVAVFDSL-EA-T----LPPRSPQ------
RLMIERLVLAKEISGIV-LE----DGG--GDDENLAVV-----RHQSFGN----WRRDMEA-AGFQLVPPSDFAIAQAKLLLRLHYPADG-------
YRL-----LVENQ-HG----------SLFLSWHDKPLVALSTW
>AtriLs .
QQLRQLLMTCAEHISRSD-----LQ-SAARILSL---L-SS----NSS-----PYGD--------S-TDRLVSQFVAA-LSLHLRL-------------
PLPQQQQ-QS------------------PRHIPPSLVTP------------------QTVESS-FLSLNQITPFIRFSHLTANQAILDALDG------
HN-S--------------VHIVDLN-TMQG-----VQWPPFMQAMAERF--------PHH------GA-------------TIRITGT------------
---------GPD-LSNLHRTGARLSRFAQTLGLD-FHFHP-F-L------------SNSLSDLLL--------------LP-------Q-A-----
IASVAQPN-----EALAVNCVHHLHVHL-------------------------------------------------------GHD--------
LPSLLRALKGLNPRVLTLSEREA-----------GH-N-HPSFTERFS-------EALSHYSALFESL-EA-T----VPPSSRE------
RMAVEQVWFGREITRVL-AN----EEEI-G-----RRERERE-RHERFER----WAEMMRV-CGFEGVVLSDFAVSQARLLLRLHYPSEG-------
YNL-------HLH-HD----------ACFLGWQNHPLFSVSSW
>PaLs .
AHLRNLLTECAEYVGQCA-----WQ-RADRMISI---L-ST----RAS-----TRGD--------S-TERLVAQFTQA-LKIRSDL-AI--------
ARPPPQ-----------------------------PAGATE------------------REIESA-YLCLNQVTPFIRFAHLTANQAILEALDT------
DSPS--------------IHVVDIN-IMQG-----VQWPPFMQALAERP--------AGP------PG-------------HIRLTGA------------
---------GTD-AEALRRTGRRLERFAHTLGLR-FEFNP-WIL------------DDDISSL---------------------------A-----IST-
-RPG-----ESLAINCVLHLHELL-----------------------------------------------------DEAHN------
EKLKSFLVRIRSLEPKVVALAEREA-----------DH-N-RPGFADRFA-------EALNHYSALFESL-EA-T----LPPRSQE------
RLDVEQVWFGREITNIV-AL----EGH--G-----R----SE-RHQRFER----WTEIMNE-CGFTSLSLSEFALSQARLLLRLHYPSEG-------
YQL-------HSR-NN----------AAFLGWQNTPLFSVSSW
>AcLs .
AQMRRLLITCADFLSQSN-----FS-AAHRLVSF---L-SS----TSS-----PHGD--------S-TERLVHQFVRA-LTIRLNR-AS-----------
----AS-MISSTTPSLSSCVPHPHFLPPASVASSSTSSGLTLLELEVVEEEEALLHHQLQSS-FICLNQVTPFIRFSHLTANQAILEAIE-------
GQES--------------IHILDFDTIMQG-----VQWPPLMQAIAER---------SNP------PN-------------MIRITGI------------
---------GYD-LETLARTGQRLENFAKSLGLR-FHFHP-LLL-L------------DSTSVAL-----------QL--S-------S-S-----ITL-
-LQE-----ETLVVNCVLYLHRLLNRND--------------------------------------------------DDYE--------
VRLFLHTIKAMNPKVVTLAEREA-----------NL-N-NPLFSQRFL-------EALDHYTALFDSM-EA-T----LPPNNTE------
RLAIEQVWLGREIVDVV-SA----EGE--E-----R----KE-RYERFQN----WAEKMRR-SGFSNVPLSPFAVAQAKLLLRLHYPSEG-------
YQL-------HIL-ND----------SLFLGWQNHALFSVSSW
>VvLs .
```

```
IQMRQLLISCAELVSQSD-----FS-AARRLLSI---L-SS----NSS-----PFGD--------S-TERLVHQFSAA-LSLRLSR--------------
-----------------------------YATPATSSGAM-----SASANTAAADSEAFHST-YLSLNQITPFIRFSQLTANQAILEAIE-------
GQRA--------------IHILDFD-IMHG-----VQWPPLMQAIAER----CGN--LHP------PP-------------MIRITGT------------
---------GED-LGILQRTGDRLLKFAQSLGLK-FQFHP-LLL------------RNDPTSVPL-----------YL--P-------S-A-----LQL-
-LPD-----ETLAVNCVLYLHRLL--KD--------------------------------------------------DSRD--------
LRLFLHKIKAMEPKVVTIAEREA-----------NH-N-HPLFLQRFV-------EALDHYTAVFDSL-EA-T----LPPTSRE------
RLAVERIWFGREIVDIV-SA----EGD--N-----R----RE-RHERFES----WEVMLRS-SGFSNVPLSPFALSQAKLLLRLHYPSEG-------
YRL-------QII-ND----------SFFLGWQNQALFSVSSW
>TcLs .
THMRQLLISCAELVSQSD-----FP-AANRLLSI---L-SS----NSC-----LYGD--------S-TERLVHQFTKA-LSLRINR-LH-----------
----GP-GSLLMMMN-INYPIA---------AATATATAIANY--D--------IDPSLQSC-YLSLNQITPFIRFAHLTANQAILEAIQV------
GQQS--------------IHILDFD-IMHG-----VQWPPLMQALAER----SANT-LHP------PP-------------MLRITGT------------
---------GHD-LNILQRTGDRLFKFAQSLGLR-FQFHP-LLV-L----------NNDPTSVAL-----------NL--P-------S-T-----LTI-
-LPD-----EALAVNCMFYLHRLL--KD--------------------------------------------------DSRD--------
LRLFLHNIKAMNPAVVTIAEREA-----------NH-N-HPLFLQRFI-------EALDYYTAIFDSL-EA-T----LPPNSRE------
RLAVEQIWFGREIVDIV-AA----EGE--N-----R----RE-RHERLET----WEVILRS-TGFSNVPLSPFAHSQAKLLLRLHYPSDG-------
YRL-------QIL-NN----------SFFLGWQNRALFSVSSW
>GrLs .
LKMRQLLISCAELVSQSD-----FP-AAIRLLSI---L-SS----SSS-----PSGD--------S-IERIVYQFVRA-LSLRINR-LHH-------
HHHHHGLAG-SSLMRMMNDISFPI------------TSTAGPV-NY--D--------IDPSLQSC-YLSLNQITPFIRFAHLTANQAILEAIQV------
GQQC--------------IHIIDFD-IMQG-----VQWPPLMQALAER----SANT-HHP------PP-------------MLRITGT------------
---------GHD-LNILHRTGDRLFKFAQSLGLR-FEFHP-LLV-L----------NEDPTWIAT-----------NF--T-------S-M-----VTI-
-LPD-----EALAVNCMLYLQRLL--KD--------------------------------------------------DSRD--------
IRLFLHHMKALNPTVVTVAEREA-----------NH-N-HPLFLQRFV-------EALDYYTAIFDSL-EA-T----LPPNSRE------
RLAVEQIWFGREIVDIV-AA----EGE--N-----R----RE-RHERLET----WEVILRS-SGFINVPLSPFAHSQAKLLLRLHYPSEG-------
YQL-------QIV-NN----------CFFLGWQNRPLFSVSSW
>MeLs1 .
IHMRQLLITCAELISQSD-----FS-AAHRLISV---L-SS----GSS-----PYGD--------S-RERLVHQFVKA-LSLRLNP---------H----
----AA-AISCALMNIGNTTLS-VGAAG-GASSAATGDA--NV--GSLISREY-EEEALQSC-YLSLNQITPFIRFSHLTANQAILEAIEV------
GQQA--------------IHIIDFD-IMHG-----VQWPPLMQALAER----SSNN-LSP------PP-------------VLRITGT------------
---------GHD-LNILHRTGDRLFKFAQSLGLK-FQFHP-LLV-S----------NNDPSSL-----------------P-------S-A-----ITL-
-LPD-----EVLAVNCVHYLHRLL--KN--------------------------------------------------DSRD--------
LRIFLHRIKALNPKVVTIAEREA-----------NH-N-HPFFFQRFL-------EALDHYTAIFDSL-EA-T----LPPNSRE------
RLAVEQIWFGREIMDIV-AA----EGE--D-----R----RE-RHERFES----WEMMLRS-TGFTNVPLSPFALSQAKLLLRLHYPSEG-------
YHL-------QIL-SN----------SFFLGWQNHALFSVSSW
>MeLs2 .
IHMRQLLITCAELISQSD-----FS-AAHRLISV---L-SA----SSS-----PYGD--------S-RERLVHQFVKA-LSLRLNP---------H----
----GN-AMATVLMNITNLPSS-GGGGGIGSASTATVSAI-NV--GSLFTRE--SEEALQSC-YLSLNQITPFIRFSHLTANQAILEAIEV------
GQRA--------------IHIIDFD-IMHG-----VQWPPLMQALAER----SSNT-LYP------PP-------------ALRITGT------------
---------GHD-LNTLNRTGDRLLKFAQSLGLK-FQFHP-LLL-L----------NTDPSSL-----------------P-------F-A-----ITL-
-LPD-----EALAVNCVLYLHRLL--KD--------------------------------------------------DSRG--------
LRIFLNRIKALNPKVVTIAEREA-----------NH-N-HPFFFQRFL-------EALDHYTALFDSL-EA-T----LPPNSRE------
RLAVEQIWFGREIMNIV-AA----DGE--D-----R----RE-RHERFES----WEMMLRS-TGFTNVPLSPFALSQAKLLLRLHYPSEG-------
YHI-------EIV-NN----------SFILGWQSHSLFSVSSW
>PtLs1 .
FHTRQLLVSCADLISQSD-----FS-AAQRLLSH---L-LST--YNSS-----PYGD--------S-TERLVHQFVRA-LSLRLN------------
RHANPARST-TTAPLVFNMNSIAPP-------PPPPCTTTNTNNNK--RMVISYESMDQDTLQSC-YLSLNQITPFIRFSHLTANQAILEAIQV------
GQQA--------------IHIIDFD-IMHG-----VQWPPLMQALADR----SNNT-LHP------PP-------------MLRITGT------------
---------GHD-LSILHRTGDRLLKFAHSLGLR-FQFHP-LLL-L----------NNDPASLAL-----------YL--S-------S-A-----ITL-
-LPD-----EALAVNCVLCLHRFL--MD--------------------------------------------------DSRE--------
LLLLLHKIKALNPNVVTVAEREA-----------NH-N-HLLFLQRFL-------EALDHYTALFDSL-EA-T----LPPNSKE------
RLSVEQIWFGREIMDIV-AA----EGE--G-----R----RE-RHQRFET----WEMMLKS-SGFSNVPLSPFALSQAKLLLRLHYPSKG-------
YQL-------QIV-NN----------SFFLGWQNHSLFSVSSW
>PtLs2 .
FQLRQLLVTCADLITQSD-----YS-AAKRLLSI---L-SS----NSS-----PYGD--------S-IERLVYQFVRA-LSLRLDR------------
HGIPT-SP-APAPHVFNINNIVH--------TSPPCGT-----NN--KMLNSYDS-DQETLRSC-YLSLNQITPFIRFSHLTANQAILEAVQG------
GQQA--------------IHIIDFD-IMHG-----VQWPPLMQALADR----PNNT-LHP------PP-------------MLRITGT------------
---------GHD-LNILHRTGDRLLKFAQSLGLR-FQFHP-LLL-L----------NNDPTTLAL-----------YL--P-------S-A-----ITL-
-LPD-----EALAVNCVLYLHRFL--KD--------------------------------------------------DSRE--------
LLLFLHKIKALNPKVVTVAEREA-----------NH-N-QPLFLQRFL-------EALDHYKALFDSL-EA-T----LPPNNRE------
RLAVEQIWFGREILDIV-AA----EGE--G-----R----RE-RHQKFET----WEMMLKS-VGFNKVPLSPFALSQAKLLLRLHYPSEG-------
YQL-------QIL-KN----------SFFLGWQNHSLFSISSW
>PtLs3 .
FHMRQLLVSCADLISQSD-----YS-AAKRFFSI---L-SS----NSS-----PYGD--------S-TERLVHQFIRA-LSLRLN------------
GHGIST-ST-APAAHVFNINNMV---------TSRPCGT-----ND--KMLISYEA-DQETLRSC-YLSLNKITPFIRFCHLTANQAILEAIQV------
GQQA--------------IHIIDFD-IMHG-----VQWPPLMQALAER----SNNT-LHP------PP-------------MLRITGT------------
---------GHD-LNVLHRTGDRLLKFAQSLGLR-FHFHP-LLL-L----------NNDPTSLAH-----------YL--P-------S-A-----ITL-
-LPD-----EALAVNCVSYLHRFL--KD--------------------------------------------------DSRE--------
LLLFLHKIKALNPKVVTVAEREA-----------NH-N-HPLFLQRFL-------EALDHYTALFDSL-EA-T----LPPNSRE------
RLAVEQIWFGREIMDIV-AA----EGE--G-----R----RE-RHQRFET----WEMMLKS-VGFIKVPLSPFALSQAKLLLRLHYPSDG-------
YQL-------QIL-NN----------SFFLGWRNHSLFSVSSW
>CsiLs .
IHMRQLLISCAELFSQAD-----FS-AAHRLISI---L-SA----NSS-----PYGD--------S-IERLVHQFIRA-LSLRLN-----------LH-
----HA-NATLLMMNITT-------------TTTATTLPY-NT--TTYNDRN-----ALQSC-YLSLNQITPFIRFSHLTANQAILESLQV------
GQQS--------------IHILDFD-IMHG-----VQWPPLMQALVERF--KNSNM-LQP------PP-------------MLRITGT------------
---------GND-IEILQRTGERLLKFAQSLGLR-FQFHP-LLL-M----------NDDPTSVAF-----------YL--P-------S-A-----LTI-
-LPD-----ETLAVNCMLFLHKLL--KD-------------------------------------------------HDTRD--------
LRLFLHKIKALNPRVVTIAEREA-----------SH-N-HPLFLQRFV-------EAVDHYGAIFDSL-EA-T----LPPNSRE------
```

```
RLAVEQVWFGREIVEIV-AT----EGE--N-----R----KE-RHERFDS----WEMILRS-CGYSNVPLSGYALSQAKLLLRLHYPSEG-------
YQL-------QVL-NN----------SLFLGWQNRALFSVSSW
>CcLs .
IHMRQLLISCAELFSQSD-----FS-AAHRLISI---L-SA----SSS-----PYGD--------S-IERLVHQFIRA-LSLRLN------------LH-
----HA-NATLLMMNITT-------------TTTATTLPY-NT--TTYNDRN-----ALQSC-YLSLNQITPFIRFSHLTANQAILESLQV------
GQQS--------------IHILDFD-IMHG-----VQWPPLMQALVERF--KNSNM-LQP------PP-------------MLRITGT------------
---------GND-IEILQRTGERLLKFAQSLGLR-FQFHP-LLL-M----------NDDPTSVAF-----------YL--P-------S-A-----LTI-
-LPD-----ETLAVNCMLFLHKLL--KD-------------------------------------------------HDTRD--------
LRLFLHKIKALNPRVVTIAEREA-----------SH-N-HPLFLQRFV-------EAVDHYGAIFDSL-EA-T----LPPNSRE------
RLAVEQVWFGREIVEIV-AT----EGE--N-----R----KE-RHERFDS----WEMILRS-CGYSNVPLSGYALSQAKLLLRLHYPSEG-------
YQL-------QVL-NN----------SLFLGWQNRALFSVSSW
>CpLs .
IHMRQLLITCAELLSRSD-----FP-AAQRLLSF---L-SA----NSS-----PYGD--------S-TERLVHQFARA-LSMRITS-----------HH-
----GS-GSVLMMLNLNL-------------NDAALPTPVINS--SLEEDRYS---STSQSC-YLSLNQITPFIRFSHLTANQAILEAIEV------
GQQA--------------IHILDLD-IMQG-----VQWPPLMQALAER----SHNS-LNP------PP-------------MLRITAT------------
---------GHD-LNMLNTTGERLFRFAQSLGIR-FSFHP-LLV-L----------NDDPASLAL-----------FL-IP-------S-A-----LAI-
-LPD-----ESLAVNCVLYLHRLI--KD-------------------------------------------------DDSRG--------
LRLLLQKIKALNPTVVTVAEREG-----------NH-N-HPLFLQRFV-------DALDHYTAIFQSL-EA-T----LPPNSRE------
RLGVEQIWFGREIVDIV-TA----QQE--N-----R----RE-RHERFEW----WEMMMRG-AGFSNVALSPFALSQAKLLLRLHYPSEG-------
YKL-------QIL-NN----------SFFLGWHNRRLFFVSSW
>StLs .
IQIRQLLISCAELISRSD-----FS-AAKRLLTI---L-ST----NSS-----PFGD--------S-TERLVHQFTRA-LSLRLNR-YI-----------
----SS-TTNHFMTPVET-------------TPTDSSSSL-----------PSSSLALIQSS-YLSLNQVTPFIRFTQLTANQAILEAING------
NHQA--------------IHIVDFD-INHG-----VQWPPLMQALADR----------YP------AP-------------TLRITGT------------
---------GND-LDTLRRTGDRLAKFAHSLGLR-FQFHP-LYI-ANN--NR--DHDEDP-SI-----------------I-------S-S-----IVL-
-LPD-----ETLAINCVFYLHRLL--KD-------------------------------------------------REK----------
LRIFLHRVKSMNPKIVTIAEKEA-----------NH-N-HPLFLQRFI-------EALDYYTAVFDSL-EA-T----LPPGSRE------
RMTVEQVWFGREIVDIV-AM----EGD--K-----R----KE-RHERFRS----WEVMLRS-CGFSNVALSPFALSQAKLLLRLHYPSEG-------
YQL-------GVS-SN----------SFFLGWQNQPLFSISSW
>SlLs .
IQIRQLLISCAELISQSD-----FS-AAKRLLTI---L-ST----NSS-----PFGD--------S-TERLVHQFTRA-LSLRLNR-YI-----------
----SS-TTNHFMTPVET-------------TPTDSSS--------------SSSLALIQSS-YLSLNQVTPFIRFTQLTANQAILEAING------
NHQA--------------IHIVDFD-INHG-----VQWPPLMQALADR----------YP------AP-------------TLRITGT------------
---------GND-LDTLRRTGDRLAKFAHSLGLR-FQFHP-LYI-ANN--NH--DHDEDP-SI-----------------I-------S-S-----IVL-
-LPD-----ETLAINCVFYLHRLL--KD-------------------------------------------------REK----------
LRIFLHRVKSMNPKIVTIAEKEA-----------NH-N-HPLFLQRFI-------EALDYYTAVFDSL-EA-T----LPPGSRE------
RMTVEQVWFGREIVDIV-AM----EGD--K-----R----KE-RHERFRS----WEVMLRS-CGFSNVALSPFALSQAKLLLRLHYPSEG-------
YQL-------GVS-SN----------SFFLGWQNQPLFSISSW
>MgLs .
LQMRQLLISCAELISRSD-----LS-AAHRLISI---L-SS----NSS-----PYGD--------S-TERLVHQFTRA-LSLRLTR-YT-----------
----TA-AAASTSI------------------ISTTIA--------------GRDDALVQSS-YLSLNQVTPYIRFSQLTANQAILEAIDG------
AHHA--------------IHILDFD-IMHG-----VQWPPLMQAVAER----------YP------LP-------------TLRITGT------------
---------GSD-LEILRRTGDRLAKFAHSLGLR-FQFHP-LIL-L---------PGEDPISV-----------------A-------S-S-----VLL-
-LPD-----ETLAVNCVHYLHRLL--RD-------------------------------------------------RDR----------
LALFLHRIKAMSPRVVTVAEREA-----------NH-N-HPIFQQRFL-------EAVDHYAAVFDSL-EA-T----LPPNSRE------
RLAVEQIWFGREIADIV-AA----EGE--S-----R----RE-RHERFRS----WEVI----------------------------------------
TL-------HII-ND----------SFFLGWQNHPLFSVSSW
>AmERA .
IHMRQLLISCAELVARSD-----YS-AAHRLIAI---L-SS----NAS-----PFGD--------S-TERLVHQFTKA-LTLRLNR-AA-----------
----TA-NTTSFMIPI-I-------------NPNTTTS--------------ANEEALLQSS-YLSLNQVTPFIRFSQLTANQAILEAIDG-------
QQA--------------IHILDFD-IMHG-----VQWPPLMQAVAER----------FP------PP-------------TLRITGT-------------
--------GKD-IEILRKTGDRLTKFAHSLGLR-FQFHP-IT-------------NQDPISI-----------------T-------S-S-----VIL--
LPD-----ETLAVNCVHYLHHLL--ND-------------------------------------------------RDR----------
LRLFLHRIKAMNPRVFTVAEREA-----------NH-N-HPLFQQRFL-------EALDHYAAVFDSL-EA-T----LPPNSRE------
RMAVEQIWFGREIADIV-AN----EGE--N-----R----KE-RHERFRS----WELVLRN-SGFGNVPLSTFALSQAKLLLRLHYPSEG-------
YQL-------HVL-QD----------SCFLGWQNQPLFSVSSW
>EgLs .
INTRQLLVTCAELLSLSN-----FA-PAHRLISI---L-SA----NSS-----PYGD--------S-TERLVHQFSRA-LSIRLHR--------------
----SA-VAAAAPPNRSSLLFP-------HRSNSSTVFGFGVD--SAVRPGQGSDPSALQSC-YLTLNQVTPFIRFSHLTANQAILEAID-------
GQRA--------------VHIVDLD-IMHG-----VQWPPLMQAIVERSSTNDKNA-LLL------PP-------------TLRITGA------------
---------GHD-LEALHRTGDRLSTFAQSLGLE-FQFCP-LLL------------PHDALSVAL-----------HAAIP-------S-E-----IST-
-LPD-----EALAVNCVLCLHRLL--KD--------------------------------------------------DARE--------
LSIFLRKIKALRPRVVTVAEREA-----------SH-N-HPMFLQRFV-------EALDHYTAVFDSL-EA-T----LPPSSRE------
RLALEQAWFGREIADII-GE----EGE--G-----R----LE-RHERFES----WEVLLRS-SGFVNVPLSPFALSQAKLLLRLHYPSEG-------
YQL-------QVK-NS----------SVFLGWQSRALFTVSSW
>PpLs .
--MRQLLITCAELISQLD-----FS-SARRLISL---L-SS----KSS-----PFGD--------S-TERLTHQFVKA-LSLRLN---------------
NPNPSS-S-----AALTT-----------AATAAASSSSNYL---LLEEEDDNNNEEALHSC-YLTLNQITPFIRFSHLTANQAILEAIDS------
SHHS--------------IHILDFD-IMHG-----VQWPPLMQALTDRS-YNSDRTVQHP------PP-------------MLRITAT------------
---------GHS-LALLLKTGDRLLKFANSLGLA-FHFHP-LVL-NDA--VQ----PSDIIS------------------P-------S-T-----LGL-
-LPN-----EALAVNCVLYLHTLV--TD--------------------------------------------------DSRE--------
LSLFLRKIKSLNPKVLTIANKEA-----------NH-N-HPLFFNRFV-------EALEHYGAVFDSL-EA-T----LPPNSRE------
RQAVEDVWMGREIRDVV-GA----EEG--R-----R----RQ-RHEKYETY---WEVMLRR-AGFENVALSPFALSQAKLLLRLHYPSEG-------
YQL-------RII-ND----------SFFLGWQNRPLFSVSSW
>MdLs1 .
THLRHLLITCAELISHLD-----FP-SANRLISL---L-SS----NSS-----PXGD--------S-TERLTHYFVKA-LSFRLNT--------------
NPNYSX-TTICPMTALTT-----------AASASASSSSNYLFPPMLLEEEDD-NEETLHSC-YLTLNQITPFIRFSHLTANQAILESIDS------
SHLS--------------IHILDFD-IMHG-----VQWPPLMQALVERS-YNSP---LHP------PP-------------TLRITAT------------
```

```
---------GRD-LTLLLRTGERLLRFAQSLGLA-FQFHP-LVL-NNA--AR----PSDLIS------------------P-------S-T-----LGL-
-HQN-----EALAVNCVLYLHTLV--TD--------------------------------------------------DSRD--------
LLLFLQKIKSLNPKVFTVANKEA-----------NH-N-NPLFFNRFV-------EALDHYGAVFESL-EA-T----LPPNSRE------RLAVEE-
WXGREVKDVV-GV----EGE--K-----R----KQ-RHERYETF---WEVVLRR-AGFENVALSPFALSQAKLLLRLHYPSEG-------YQL-------
RIL-ND----------SFFLGWQNRPLFSVSSW
>MdLs2 .
THLRHLLITCAELISHLD-----FP-SAHRLISL---L-SS----NSS-----PFGD--------S-TERLTHHFVKA-LCFRLNT--------------
NPNYSP-TTIGPLTALTN-----------AASASASSSSNYLFLPVLQEEEEDNNEEALHSC-YLTLNQITPFIRFSHLTANQAILEAIDA------
SHLS--------------IHILDFD-IMHG-----VQWPPLMQALVERS-YNSP---LHP------PP-------------TLRITAT------------
---------GRD-LSLLLRTGERLLRFAQSLGLA-FQFHP-LVL-NNT--SP----PSDLIS------------------P-------S-T-----LGL-
-HQN-----EVLAVNCVLYLHTLV--TD--------------------------------------------------DSRD--------
LFLFLQKIKSLNPKVFTIANKEA-----------NH-N-NHLFFNRFV-------EALDHYGAVFDSL-EA-T----LPPNSRE------RLVVEE-
WFGREIKDVV-GV----EGE--K-----R----KQ-RHESYETF---WEVVLRR-VGFENVPLSPFALSQAKLLLRLHYPSEG-------YQL-------
RMF-ND----------SFFLGWQNRPLFSVSSW
>FvLs .
--ARQLLIRCAELISQLD-----FS-SAHGLISI---LASS----NYS-----PHGD--------S-TERLVHQFVRA-LSLRL----------------
PPVAAA-PPDMARVAAAT-----------GTSAASTSSA------LSLEVETEAEEETLQSC-YLTLNQITPFIRFSHLTANQAILEAIDS------
SHHA--------------IHILDFD-IKHG-----VQWPPLMQALVERS-YGSS---SSP------PP-------------LLRITAT------------
---------GRD-LTLLRRTGERLLRFAQSLGLT-FHFRP-IVL-LND--VA----MIDYLN------------------P-------A-S-----LGL-
-FPN-----EALAVNCVLYLHRLL--TD--------------------------------------------------DARD--------
LHLFLDKIRALNPKIVTVAEREA-----------NH-N-SPMFLNRFV-------EAVDHYGAVFGSL-EA-T----LPPNSRE------
RLAVEQMWFGREIADVV-AA----DDD--QG----R----KQLRHERYEN----WEMMMRR-SGFSNVPLSPFALSQAKLLLRLHYPSEG-------
YQL-------HSL-KD----------SFFLGWMNRPLFSVSSW
>CsLs .
LQMRQLLIRCAHFISQSD-----FI-SAHHLLSI---L-SS----NSS-----PYGD--------S-TQRLLHYFSSS-LSHLL----------------
-------------------------------PSSNYNSSFHHH---------HHDIEKIQSC-YLSLNQITPFIRFTHLTANQAILEGIE-------
ESGM--------------IHVLDFD-IMHG-----VQWPPLMQALADR----------FP------SP-------------MLRITAT------------
---------GVD-LNFLHKTGDRLSKFAQSLGLR-FQFHP-LLL-L---------HDRDHHRV----------------IP-------A-A-----LTL-
-FPD-----EALAVNCVLYLHRLM--KD-----------------------------------------------------D--------
VRVLLNKIKALNPKVVTIAEKEA-----------NF-N-HPLFMQRFV-------EALNHYTLLFDSL-EA-T----LPPNSRE------
RLAVEQVWFGREINDIV-SG----EVN--K-----K----KQHYAERYES----WETMLKS-LGFSNIPLSPFALSQAKLLLRLHYPSEG-------
YHL-------QIL-HD----------SLFLGWQNQPLFSVSSW
>RcLs .
IYMRQLLISCAELISQSD-----FS-AAHRLISV---L-SS----SSS-----PYGD--------S-RER------------------------------
----------------------------------------------------------------------------------------------------
----------------------------------------------------------------------------------------------------
----------------------------------------------------------------------------------------------------
----------------------------------------------------------------------------------------------------
----------------------------------------------------------------------------------------------------
-------------------------------------S-AGFNNVPLSPYALSQAKLLLRLHYPSEG-------YHL-------QMI-ND----------
SFFLGWQNRALFSVSSW
>MaLs1 .
IHARQLLISCADLVHRGD-----LP-AADRAISI---L-SA----AAS-----PCGD--------S-TERLIRQFCRA-LSVRVGR-V------------
-------------------------------SPSAESL------------------GSLRSS-YLSFNQISPFLRFSHLTPNQAILEA------------
----------------------------------------AIAERSD-------PSD------PP-------------FIRITGT---------------
------GSN-LEVLRRTGHRLQNFAHSLGLG-FQFHP-LLL--------------HPTSTSL-----------NF-TP-------S-P-----FRL--
HPG-----ETLVVNCVLFLHKLQ--KE---------------------------------------------GGNEDGSRD--------
LQAFLRTIRAMNPSVVTVAEREA-----------SH-N-SPNFMQRFV-------EALDYYMAVFESL-EA-T----LPPTSQE------
RLAVEQVWLGQEIEGIV-GG----EGG--G----------HE-RSER-------WENVLRD-AGFSSVQLSNFAVSQARLLLRLHYPSEG-------
YQV-------ELV-RD----------SLFLGWQNRHLFSVSSW
>MaLs2 .
VNARQLLISCAELVHCGD-----LP-AAERAISI---L-TA----AAA-----PYGD--------S-IDRLIRQFCRA-LSVRIGR-V------------
-------------------------------SPSAASL------------------GSLQSS-YLLFNQMTPFLRFSHLTANQAILEA------------
----------------------------------------AIADRSD-------PGD------PP-------------FIRISGT---------------
------GSS-LETLQRTGDRLRNFAHSLGLE-FQFHP-LLL-P---------PSVHSTNTSY-----------NF-TP-------S-C-----LQF--
HPS-----ETLVVNCVLFLHKLQ--RE---------------------------------------------DGNDDGSRK--------
LQAFLRTIRVMNPSVVTVAERET-----------VH-S-SRSFMQRFV-------EALDYYTAVFEAL-EA-T----LPPTSEE------
RMAVEQVWLGREIESIV-GG----EGD--G-----R----RE-RHER-------WDSLMRD-AGFSSLAPSTFAVSQARLLLRLHYPSEG-------
YQL-------QLV-RD----------SFLLGWHSRHLFSVSSW
>MaLs3 .
AHARQLLLSCAELAHRGD-----LP-AAQRTASL---L-LA----TAS-----PYGD--------S-TDRLVHQFARA-LSLRVER--------------
-----------------------------LLPPVVDAAS----------------PEALQSS-YLSFNQITPFLRFAHLTANQAILEA------------
--------------------------------------------RSD-------PKD------PP-------------SIRVTGT---------------
------GSN-LEVLRRTGDRLQTFADSLNLH-FQFHP-LLL-PST-------SSNPLSSTSA-----------DL-TS-------S-S-----FQI--
HPG-----EILAVNCVLFLHKLL--KD---------------------------------------------GGGSDGSHD--------
LRAFLQAVRAMNPAVVTVAEREA-----------SH-N-SPIFLQRFT-------EALDYYTAVFESL-EA-T----LPPTSRE------
RVAVEQVWLSKEIEDVV-SR----EGD--G-----R----RE-RHERFEW----WEALMRR-AGFTNLPLSPFALSQARLLLRLHYPSEG-------
YQL-------QMV-RD----------SFFLGWQKKSLFSVSSW
>MaLs4 .
APVRQLLVSCAELVHRGD-----LP-AARHTGSL---L-LA----TAS-----PYGD--------S-TDRLVHQFARA-LSLRVDP--------------
-----------------------------LFPS-VDAAS----------------PEALQSS-YLSFNQITPFLRFAHLTANQAILEA------------
--------------------------------------------RSD-------PNN------PP-------------SIRITGT---------------
------GRN-LDVLRRTGDRLQTFADSLGLG-FEFHA-LLF-PST-------TSDPSSSTTT-----------DF-TS-------S-S-----LRL--
HPG-----EILTVNCVLFLHNLL--QD---------------------------------------------GSGSDDSRD--------
LRAFLQAVRAMNPAVVTVAEREA-----------NH-N-APIFFQRFM-------EALDYYTAVFESL-EA-T----LPPTSRE------
RAAVEQVWLGREIEDVV-AR----EGE--R-----R----RE-RHERFDR----WETLMRG-AGFTNLPLSPFALSQARLLLRLHYPSEG-------
YQL-------HTV-RD----------SFFLGWQNKPLFSVSSW
>PdLs1 .
```

TRSCQLLIGCAELIHRAD-----YS-AARRILSL---L-SN----ISS-----PHGD--------S-TDRLVHQFTRA-LSLHIDS-L---------TH-
-----------------------------LLPPSFFSSN----------------GDTLQSS-YLSLNKVTPFLRFAHLTANQAILEAVD-------
GRQS--------------IHILDFD-TSHG-----VQWPPLLQAIAERSE-------PSN------RP-------------SVRITGT------------
---------GTD-LDVLHRTGDRLKAFASSLGLR-FQFHP-LHL-P---------TTNPDTSASF-----------------------S-S-----FQP-
-HPG-----ETLAVNCTLFLHKLL--RD--RGSDDPACK--------
LMGFLRAVKEMNPAVVTVAEREA-----------NH-N-SPVFLQRFM-------EALDHYAAVFESL-EA-T----LPPKSRE------
RQEVEEMWLGREIEDIV-GK----EGE--E-----R----RE-RHERFEW----WEALMRG-RGFSSLQLSPFALTQAKLLLRLHYPSEG-------
YQL------IQMS-KN----------CLFLAWQHKPLFSVSSW
>PdLs2 .
THSCRLLISCAELIHRAD-----YS-AARRILSL---L-SN----ISS-----PHGD--------S-TDRLVHQFARS-LSLRIDR-L---------TH-
-----------------------------LISPSLFSDN----------------EDTLQSS-YLSLNKVTPFLRFAHLTANQAILEAID-------
GHRS--------------IHILDFD-TSHG-----VQWPPFLQAIAERSD-------PSS------LP-------------SIRITGT------------
---------GTG-LDVLRRTGERLQAFANSLGLR-FQFHP-LYL-P---------TADPDASASL---------------N-------S-S-----FQL-
-HPG-----ETLAVNCMLFLHKLL--KD--GSSDDPACK--------
LVAFLHAVKAMNPVVVTVAEREA-----------NH-N-SPIFLRRFM-------EALDHYTAVFESL-EA-T----LPPKSPE------
RLEVEQVWLGREIEDIV-GR----EGE--G-----R----KE-RHERFEW----WEGLMRG-RGFSSLPLSPFALSQAKLLLRLHYPSEG-------
YQL-------PMS-KN----------SLFLGWQHKPLFSVSSW
>PdLs3 .
AHARQLLISCAERIHCGD-----LP-TARRTASL---L-SA----AAS-----PYGD--------S-ADRLTHQFARA-LSLRLDS---P--SR---LA-
-----------------------------SPSPSAASSS----------------SEALQSS-YLSLNQVTPFLRFAHLTANQAILEALD-------
GCRR--------------IHILDFD-TSHG-----VQWPPLLQAIAERSN-------ADG------PP-------------SIRITGT------------
---------GTD-LSVLRRTGDRLQTFAHSLGLQ-FQFHP-LLL-PIS--ST--NSSSPSSSTSTSILSTTTTTTNL-TS-------S-S-----FQL-
-HPG-----ETLAVNCVLFLHKLL--QDQD--SRSEDDGSRE--------
LRAFLQAVKALNPAAVTVAEREA-----------SH-N-SPIFLQRFM-------EALDYYTVVFESL-EA-T----LPPKSQE------
RLAVEQVWLGREIEDIV-AW----EGE--G-----R----RE-RHERFAR----WEGLMRD-AGFSNLPLSPFALSQAKLLLRLHYPSEG-------
YQL------QMV-RD----------CIFLGWQNKALFSVSSW
>AtLAS .
HHLRRLLFTAANFVSQSN-----FT-AAQNLLSI---L-SL----NSS-----PHGD--------S-TERLVHLFTKA-LSVRINR-----------
QQQDQT-AE-TVATWTTNEMTM-----------SNSTVFTSSVCKE--QFLFRTK-NNNSDFESCYYLWLNQLTPFIRFGHLTANQAILDATET-----
NDNGA--------------LHILDLD-ISQG-----LQWPPLMQALAER----SSNP-SSP------PP-------------SLRITGC-----------
----------GRD-VTGLNRTGDRLTRFADSLGLQ-FQFHT-LVI-V----------EEDLAGLLL-----------QI-RL-------L-A-----
LSA--VQG-----ETIAVNCVHFLHKIF--ND--DGDM--------
IGHFLSAIKSLNSRIVTMAEREA-----------NH-G-DHSFLNRFS-------EAVDHYMAIFDSL-EA-T----LPPNSRE------
RLTLEQRWFGKEILDVV-AA----EET--E-----R----KQ-RHRRFEI----WEEMMKR-FGFVNVPIGSFALSQAKLLLRLHYPSEG-------
YNL-------QFL-NN----------SLFLGWQNRPLFSVSSW
>EsLs .
HHLRRLLFTAADFVSQSN-----FS-AAQNLLSI---L-SA----NSS-----PYGD--------S-TERLVHLFSKA-LSIRINR-----------
QQQDPT-AE-NVATWTTNEMT-------------SSTVFTSSVCKE--QFLFRTKNNNNSEFESCYYLWLNQLTPFIRFGHLTANQAILDATET-----
NGDGA--------------LHILDLD-ISQG-----LQWPPLMQALAER----SSNP-NSP------PP-------------SLRITGC-----------
----------GRD-VTGLNRTGDRLTRFANSLGLQ-FQFHT-LVI-V----------EEDLAGLLL-----------QI-RL-------L-A-----
LSA--VQG-----ETIAVNCVHFLHRFS--ND--DGDM--------
IGHFLSAIKSLNPRIVTMAEREA-----------NH-G-DHSFLNRFA-------EAVDHYVAIFDSL-EA-T----LPPNSRE------
RLTLEQRWFGKEILDVV-AA----KAE--E-----R----KQ-RHRRFEI----WEEIMKR-FGFVNVPIGSFAFSQAKLLLRLHYPSEG-------
YNL-------QFL-NN----------SLFLGWQNRPLFFVSSW
>CrLs .
HHLRRLLFTAADFVSQSN-----FT-AARNLLSI---L-SL----NSS-----PYGD--------S-TQRLAHLFTKA-LSLRINR---------
LQQEQDP-----TVATCTTNEMTM----------STNSTVFTSSVCKE--QFLFRTK-NNNSDFESCYYLWLNQLTPFIRFGHLTANQAILDATET----
-NDNGA--------------LHILDLD-ISQG-----LQWPPLMQALAER----SSSP-NSP------PP-------------SLRITGC----------
-----------GRD-VTGLNRTGDRLTRFANSLGLQ-FQFHK-LVI-V----------DEDLPGLLL-----------QI-RL-------L-A-----
LSA--VQG-----ETIAVNCVHFLYKFF--ND--DADL--------
IGHFLTVIKSLNPRIVTMAEREA-----------NH-G-DHSFLIRFS-------EALDHYVAIFDSL-EA-T----LPPNSRE------
RLTLEQRWFGKEIMDVV-AA----EAT--E-----R----KQ-RHRRFEI----WGEMMKR-FGFVNVPIGSFALSQAKLLLRLHYPSEG-------
YNL-------QFL-ND----------SLFLGWQNRLLFSVSSW
>LuLs1 .
IQVRQLIVRCSDLISQAD-----YS-SAHRLIGI---L-SA----ASS-----PYGD--------S-TQRLAHHFSSA-LSLRLSG--------------
-----------------------------HLPIAVV------------------DHREVASA-
YLTLNQITPFIRFTHLTANQAILESVLLGQQQPDDQGT--------------IHIVDFD-IMHG-----VQWPPLMQALAER----------HP------
PP-------------SLRITGT---------------------GHD-PETLTRTGDRLSKFAQSLGLT-FQFHP-LLL-LHA--------SAD-------
----------HL-IP-------S-A-----VTV--LPG-----ETLAVNCMFYLHRLI--NDF-------------------------------------
----------QNDDYM--------IRTFLRRIREMNPRVVTIGEREM-----------DS-----------VGSGGGIGEAVEYYQRVLESL-EE-T---
-VAPTSAD------RAEVEGVWFSKEIKEVV-NG---------G-----R-------VDHKLRW----WSEMMTS-
VGFRNVGLSPFAVSQAKLLLRLHYPSEG-------YRLDHE-DDDDGV-VS----------GLFLGWRDRPLFSLSSW
>LuLs2 .
IQVRQLIVRCSDLISQAD-----YS-TAHCLIAI---L-SA----ASS-----PYGD--------S-TQRLAHHFSSA-LSLRLSR--------------
-----------------------------HPRPAAV------------------DHRDVASA-
YLTLNQITPFIRFTHLTANQAILESVLLGQKQPDDQGT--------------IHIVDFD-IMHG-----VQWPPLMQALAER----------HP------
PP-------------SLRITGT---------------------GHD-PETLTRTGDRLSKFAQSLGLT-FQFHP--LL-FHA--------SAD-------
----------HL-IP-------S-A-----VTI--LPG-----ETLAVNCMFYLHRLI--NDF-------------------------------------
----------QNDDYM--------IRTFLRRVREMNPRVVTIGEREM-----------D--------------SGGGVGEEVEYYQRVFESL-EE-T---
-VAPTSVD------RAEVEGVWFSKEIKEVV-NG---------G-----R-------VDHKLRW----WSEMMTS-
VGFRNVGLSPFAVSQAKLLLRLHYPSEG-------YRLDHD-DDVEGV-VS----------GLFLGWRDRPLFSLSSW
>PpatLs1 .
MQLRDLLLETAQLISQCD-----WD-RARPLLQL---L-SR----RVS-----TTGD--------S-SERVASCFFEA-LATRFSR-V------------
--------SGIQINEL---------LPSRIQGPSNQE---------------------MISA-YLALNQVTPFMRFAHLTANQALLEALT-------
GENF--------------VHIVDLE-IGHG-----IQWPLFMQALADL---------RG---------------EEGYTIQHLRITGV------------
---------GQD-RDVLNRTGIRLAEFAQSINLP-FEFSP-LVQ-ISE-----------------------------HL-VP-------R-M-----LGL-
-RVG-----EAVAINCMLQLHRLL--AKGPE-------
KLISFLCMLESLTPKVVTLAELEA-----------SH-N-QPHFLDRFA-------EALNHYSTLFDSL-DA-T----LPPTSAD------

RIRVEQTWCKMEIVNIV-AC----DGA--E-----RIV-----RHQRFEL----WRRYFHR-AGFQLLSTSRFATSQARLLLRLHYPCDD-------
YQL-----LENVD-DG----------CLLLGWQDHPLFCVSSW
>PpatLs2 .
MLLRDILVDTAQYISQCD-----WE-RARPLLQV---L-RR----QVS-----STGD--------S-SERVASCFFEA-LATRFSR-V------------
--------SGTEINEL---------LSSPTQEPSSEE---------------------ILSA-FLALNQVTPFMRFAHLTANQALLEALT-------
GEDF--------------VHIVDLD-IGHG-----VQWPPFMQALADI---------RG---------------EEGHTIQHLRITGV------------
---------GKD-REMLDRTGTRLAEFAQSIQLP-FEFTP-LVQ-APE----------------------------NL-IP-------S-M-----FGL-
-RIG-----EAVAFNCMLQLHQLL--AKGSE-------
KLTSFLYMLESLTPRVVTLAELEA-----------SH-N-QPHFLDRFA-------EALNHYSTLFDSL-DA-T----LPPTSPE------
RIRVEQTWYKMEIINIV-AC----DGT--E-----RTV-----RHQRCEQ----WRRFFER-AGFQLLPTSRFATSQARLLLRLHYPCDG-------
YRL-----VEDVE-DG----------CLLLGWQDRPLFCVSSW
>OsMOC1 .
PSTRDLLLACADLLQRGD-----LP-AARRAAEIV--L-AA----AAS-----PRGD--------A-ADRLAYHFARA-LALRVDAKAGHG-HV------
-----------------------VVGGGAARPASSGA-------------------------YLAFNQIAPFLRFAHLTANQAILEAVD-------
GARR--------------VHILDLD-AVHG-----VQWPPLLQAIAERAD-----PALGP------P--------------EVRVTGA------------
---------GAD-RDTLLRTGNRLRAFARSIHLP-FHFTP-LLL-SCA-TTAPHHVAGTSTGAAA--------------AA-------STAAAATGLEF-
-HPD-----ETLAVNCVMFLHNLAG-HDE--
LAAFLKWVKAMSPAVVTIAEREA-GGGGGGG---DHID----DLPRRV------GVAMDHYSAVFEAL-EA-T----VPPGSRE------
RLAVEQEVLGREIEAAV-GP----SGG--------RWWRGIE-R-----------WGGAARA-AGFAARPLSAFAVSQARLLLRLHYPSEG-------
YLV-------QEA-RG----------ACFLGWQTRPLLSVSAW
>OsGRAS-7 .
-----LVLACADLVHRGD-----LD-GARRVAEA---VL-A----AAD-----PRGE--------A-GDRLAHHFARALLALRGGG-KG--------
GHG-------------------------GGGGGVVPSSAA--------------------------YLAYIKIAPFLRFAHLTANQAILEAAAA---
DAGGAHRR------------VLHIVDLD-AAHG-----VQWPPLLQAIADRAD-----PAVGP------PP-------------EVRLTGA---------
------------GTD-RDVLLRTGDRLRAFSSSLNLP-FRFHP-LIL-PC---TA--ELAADPTAA--------------------------------
LEL--HPD-----ETLAVNCVLFLHKLGG-DGE--
LAAFLRWVKSMNPAVVTIAERE----GVLGGDVDDD-N-VPDELPRRV------AAAMDYYSSVFDAL-EA-T----VPPASAD------
RLAVEQEILSREIDAAV-AA----PGAG-GG----G-------RARDFDA----WASAARA-AGLAPRPLSAFAASQARLLLRLHYPSEG-------
YKA-----DDDGG-RG----------ACFLRWQQRPLMSVSSW
>AtSCR .
LHLLTLLLQCAEAVSADN-----LE-EANKLLLE---I-SQ----LST-----PYGT--------S-AQRVAAYFSEA-MSARLLN-SCL--GI---
YAALPS--------------------------RWMPQTHSL-------------------KMVSA-FQVFNGISPLVKFSHFTANQAIQEAFE-------
KEDS--------------VHIIDLD-IMQG-----LQWPGLFHILASR---------PGG------PP-------------HVRLTGL------------
---------GTS-MEALQATGKRLSDFADKLGLP-FEFCP-LAE-KVG----------------------------NL-DT-------E-R-----LNV-
-RKR-----EAVAVHWLQ--HSLY---DV---TGSDAHT------------
LWLLQRLAPKVVTVVEQDL-----------SH---AGSFLGRFV-------EAIHYYSALFDSL-GA-S----YGEESEE------
RHVVEQQLLSKEIRNVL-AV----GGP--S-----RS------GEVKFES----WREKMQQ-CGFKGISLAGNAATQATLLLGM-FPSDG-------
YTL-------VDD-NG----------TLKLGWKDLSLLTASAW
>OsGRAS-41 .
LHLLTLLLQCAESVNADN-----LD-EAHRALLE---I-AE----LAT-----PFGT--------S-TQRVAAYFAEA-MSARLVS-SCL--GL---
YAPLPN--------------------------P-SPAAARLH-----------------GRVAAA-FQVFNGISPFVKFSHFTANQAIQEAFE-------
REER--------------VHIIDLD-IMQG-----LQWPGLFHILASR---------PGG------PP-------------RVRLTGL------------
---------GAS-MEALEATGKRLSDFADTLGLP-FEFCP-VAD-KAG----------------------------NL-DP-------E-K-----LGV-
-TRR-----EAVAVHWLR--HSLY---DV---TGSDSNT------------
LWLIQRLAPKVVTMVEQDL-----------SH---SGSFLARFV-------EAIHYYSALFDSL-DA-S----YSEDSPE------
RHVVEQQLLSREIRNVL-AV----GGP--A-----RT------GDVKFGS----WREKLAQ-SGFRVSSLAGSAAAQAVLLLGM-FPSDG-------
YTL-------IEE-NG----------ALKLGWKDLCLLTASAW
>AtGRAS-28 .
IKLLSLLLQCAEYVATDH-----LR-EASTLLSE---I-SE----ICS-----PFGS--------S-PERVVAYFAQA-LQTRVIS-SYLS-GA---
CSPLSE--------------------------K--PLTVVQS-----------------QKIFSA-LQTYNSVSPLIKFSHFTANQAIFQALD-------
GEDS--------------VHIIDLD-VMQG-----LQWPALFHILASR---------PRK------LR-------------SIRITGF------------
---------GSS-SDLLASTGRRLADFASSLNLP-FEFHP-IEG-IIG----------------------------NLIDP-------S-Q-----LAT-
-RQG-----EAVVVHWMQ--HRLY---DV---TGNNLET------------
LEILRRLKPNLITVVEQEL-----------SY-DDGGSFLGRFV-------EALHYYSALFDAL-GD-G----LGEESGE------
RFTVEQIVLGTEIRNIV-AH----GGG--R-----R-----------KRMK----WKEELSR-VGFRPVSLRGNPATQAGLLLGM-LPWNG-------
YTL-------VEE-NG----------TLRLGWKDLSLLTASAW
>OsGRAS-32 .
LELVRALTACADSLSAGN-----HE-AANYYLAR---L-GE----MAS-----PAGP--------TPMHRVAAYFTEA-LALRVVR-MWP--HM---FD-
-----------------------------IGPPRELTDDAFG------------GGDDDAMA-LRILNAITPIPRFLHFTLNERLLREFE-------
GHER--------------VHVIDFD-IKQG-----LQWPGLLQSLAAR---------AVP------PA-------------HVRITGV------------
---------GES-RQELQETGARLARVAAALGLA-FEFHA-VVD-RLEDV--------------------------RL----------------WMLHV-
-KRG-----ECVAVNCVLAMHRLL--RD--DA---------
ALTDFLGLARSTGATILLLGEHEG-----------GGLN-SGRWEARFA-------RALRYYAAAFDAVDAA-G----LPEASPA------RAKAEEM-
FAREIRNAV-AF----EGP--E-----R----FE-RHESFAG----WRRRMEDGGGFKNAGIGEREAMQGRMIARMFGPDK--------YTV-----
QAH------GGGGSGGGEALTLRWLDQPLYTVTAW
>AtGRAS-8 .
FELVNLLTGCLDAIRSRN-----IA-AINHFIAR---T-GD----LAS-----PRGR--------TPMTRLIAYYIEA-LALRVAR-MWP--HI---FH-
-----------------------------IAPPREFDRTV---------------EDESGNA-LRFLNQVTPIPKFIHFTANEMLLRAFE-------
GKER--------------VHIIDFD-IKQG-----LQWPSFFQSLASR---------INP------PH-------------HVRITGI------------
---------GES-KLELNETGDRLHGFAEAMNLQ-FEFHP-VVD-RLEDV--------------------------RL----------------WMLHV-
-KEG-----ESVAVNCVMQMHKTL--YD--GTGA---------
AIRDFLGLIRSTNPIALVLAEQEA-----------EH-N-SEQLETRVC-------NSLKYYSAMFDAI-HT-N----LATDSLM------
RVKVEEMLFGREIRNIV-AC----EGS--H-----R----QE-RHVGFRH----WRRMLEQ-LGFRSLGVSEREVLQSKMLLRMYGSDNEG-----
FFNV-----ERSDE-DNGGEGGRGGG--VTLRWSEQPLYTISAW
>OsGRAS-19 .
--ILQSLLSCSRAAATD------PG-LAAAELAS---V-RA----AAT-----DAGD--------P-SERLAFYFADA-LSRRLAC-G-T---------
-----------------------------GAPPSAEPDA-------------RFASDELTLC-YKTLNDACPYSKFAHLTANQAILEATG-------
AATK--------------IHIVDFG-IVQG-----IQWAALLQALATRP--------EGK------PT-------------RIRITGV------------

--PSPLL--GPQPAASLAATNTRLRDFAKLLGVD-FEFVP-LLR-PVH----------------------------EL-NK-------S-D-----FLV-
-EPD-----EAVAVNFMLQLYHLL---GDSDE-------
LVRRVLRLAKSLSPAVVTLGEYEV-----------SL-N-RAGFVDRFA-------NALSYYRSLFESL-DV-A----MTRDSPE------
RVRVERWMFGERIQRAV-GP----EEGA-D-----R----TE-RMAGSSE----WQTLMEW-CGFEPVPLSNYARSQADLLLWNYDSKYK-------
YSLV------ELP-PA----------FLSLAWEKRPLLTVSAW
>AtSCL4 .
--LLKAIYDCARISDSD------PN-EASKTLLQ---I-RE----SVS-----ELGD--------P-TERVAFYFTEA-LSNRL-----S----------
----PN-SP---------------------ATSSS-SSS----------------TEDLILS-YKTLNDACPYSKFAHLTANQAILEATE-------
KSNK--------------IHIVDFG-IVQG-----IQWPALLQALATRT--------SGK------PT-------------QIRVSGI------------
--PAPSL--GESPEPSLIATGNRLRDFAKVLDLN-FDFIP-ILT-PIH----------------------------LL-NG-------S-S-----FRV-
-DPD-----EVLAVNFMLQLYKLL---DETPT-------
IVDTALRLAKSLNPRVVTLGEYEV-----------SL-N-RVGFANRVK-------NALQFYSAVFESL-EP-N----LGRDSEE------
RVRVERELFGRRISGLI-GP----EKTG-I-----H----RE-RMEEKEQ----WRVLMEN-AGFESVKLSNYAVSQAKILLWNYNYSNL-------
YSIV------ESK-PG----------FISLAWNDLPLLTLSSW
>EsSCL4-1 .
--LLRAIYDCARILESE------SD-VAAEALVR---I-RD----SVS-----ELGD--------P-TERLGFYFTEA-LCDRLSP--------------
------------------------------DSVPKESPS----------------VEEMILS-YKTLNDACPYSKFAHLTANQAILEATE-------
NSNK--------------IHIVDFG-IVQG-----LQWPALLQALATRS--------SGK------PI-------------QVRVSGI------------
--PAPSL--GESPEPSLIATGNRLRDFAKVLDLN-FDFIP-ILT-PIH----------------------------SL-NG-------S-T-----FRV-
-DPD-----EVLAVNFMLQLYKLL---DETPT-------
IVDTALRLARSLNPIVVTLGEYEV-----------SL-N-RVAFANRMR-------NALKFYSAVFESL-EP-N----LGRDSEE------
RVRVERVLFGRRISGLI-GP----EKTG-N-----Q----RE-RMEEKEQ----WRVLMES-AGFESVKLSNYAVSQAKILLWYYNYSDL-------
YTIV------ESM-PG----------FISLAWNDLPLLTVSSW
>EsSCL4-2 .
--VLKAIHDCARNLEIK------PD-VAAETLVR---I-RE----SVS-----DSGD--------P-TERVGFYFTEA-LSKRLSS-TAK----------
----KE-TE---------------------------SPS----------------LKDFILS-YKTLNDACPFSKFAHLTANQAILEATH-------
NSSN--------------IHIVDFG-IFQG-----VQWAALLQALATRA--------SGK------PA-------------RIRISGI------------
--PAPSL--GDSPGMSLIATGNRLRDFAAVLDLN-FEFYP-ILT-PIH----------------------------LL-NV-------S-S-----FRV-
-EPD-----DFLAVNFMLELYKLL---DETAT-------
IVDNALRLARSLNPRIVTLGEYEV-----------SL-N-RVEFVNRVK-------NALRFYSAVFESL-EP-N----LERDSKE------
RLRVERVLFGRRIADLV-RPGN--NKPG-N-----R----SG-RMEEKEQ----WRVLMEK-AGFEPVKPSNYAVSQASLLLWSYNYSTL-------
YSLV------ESE-PG----------FISLAWDNVPLLTVSSW
>AtSCL7 .
--IFKAIHDYAR----K------PE-TKPDTLIR---I-KE----SVS-----ESGD--------P-IQRVGYYFAEA-LS--------H----------
----KE-TE---------------------SPSSSSSSS----------------LEDFILS-YKTLNDACPYSKFAHLTANQAILEATN-------
QSNN--------------IHIVDFG-IFQG-----IQWSALLQALATRS--------SGK------PT-------------RIRISGI------------
--PAPSL--GDSPGPSLIATGNRLRDFAAILDLN-FEFYP-VLT-PIQ----------------------------LL-NG-------S-S-----FRV-
-DPD-----EVLVVNFMLELYKLL---DETAT-------
TVGTALRLARSLNPRIVTLGEYEV-----------SL-N-RVEFANRVK-------NSLRFYSAVFESL-EP-N----LDRDSKE------
RLRVERVLFGRRIMDLV-RSDDDNNKPG-T-----R----FG-LMEEKEQ----WRVLMEK-AGFEPVKPSNYAVSQAKLLLWNYNYSTL-------
YSLV------ESE-PG----------FISLAWNNVPLLTVSSW
>CrSCL4-1 .
--ILKAIYEYAR----K------PG-TIPETLTQ---I-RE----SVS-----ELGN--------P-IERVGFYFVEA-LS--------H----------
----KE-TE---------------------PSPAA-SSS----------------LEEFILS-YKTLNDACPYSKFAHLTANQAILEATS-------
QSQN--------------IHIVDFG-IFQG-----IQWSALLQALATRA--------SGK------PT-------------RIRISGI------------
--PAPSL--GDSPEPSLIATGNRLRDFAAILDLN-FEFNP-VLT-PIQ----------------------------LL-TG-------S-S-----FRV-
-DPD-----EVLVVNFMLELYKLL---DETAT-------
GVGTALRLARSLNPRIVTLGEYEV-----------SL-N-RVEFANRVK-------NSLRFYSAVFESL-EP-N----LERDSKE------
RQRVERVLFGRRISELV-RTGNVNIKPG-T-----R----LG-LMEEKEQ----WRVLMEK-AGFEPVKPSNYAVSQAKLLLWNYNYSTL-------
YSLV------ESE-PG----------FISLAWNNVPLLTVSSW
>CrSCL4-2 .
--ILKAIYDYAR----K------PG-TKPEALIQ---I-RE----SVS-----ELGN--------P-IERVGFYFAEA-LS--------H----------
----KE-TE---------------------PSPAASSSS----------------LEEFILS-YKTLSDACPYSKFAHLTANQAILEATS-------
QSQN--------------IHIVDFG-IFQG-----IQWSALLQALATRA--------SGK------PI-------------RIRISGI------------
--PAPSL--GDSPEPSLIATGNRLRDFAAILDLN-FEFNP-VLT-PIQ----------------------------LL-TE-------S-S-----FRV-
-DPD-----EVLVVNFMLELYKLL---DETAT-------
GVGTALRLARSLNPRIVTLGEYEV-----------SL-N-RVEFSNRVK-------NSLRFYSAVFESL-EP-N----LERDSKE------
RSRVERVLFGRRISELV------------------RMD--NG-LMEEKEQ----WRVLMEK-AGFEPVKPSNYAVSQAKLLLWNYNYSTL-------
YSLV------ESE-PG----------FISLAWNNVPLLTVSSW
>MgSCL4 .
--ILKALIDCARLSESD------PE-NAMASLIQ---L-QD----SVS-----RVGD--------P-VERVSYYFSEA-LCSRVST---R----------
----AE-KT----------------------STIYDTTS-----------------EEFTLS-YKALNDACPYSKFAHLTANQAILEATE-------
KANK--------------IHIVDFG-IVQG-----IQWAAFLQALATRP--------SGK------PD-------------RIRISGI------------
--PAPSL--GNSPAASLFATGNRLRDFAKLLDLR-FEFDP-ILT-PVH----------------------------EL-EG-------S-S-----FRV-
-DPD-----EVLAVNFMLQLYNLL---DESND-------
GVEDALKLAKSLNPCVVTLGEYEV-----------SL-N-RVGFLHRFK-------NGLKYYTAIFESL-EP-G----LARDSPE------
RLQAERLLLGRRIAGVV-GP----EE---R-----K----RE-RMEDKER----WRFLMEG-AGFQPLVFSHYAQSQAKILLWNYTNSSF-------
YKLL------DS-------------------------------
>SlSCL4 .
--LLKSLVECARLAESE------PE-NVVKSLIR---L-RE----SVS-----QQGD--------P-MERVGFYFLEA-LYNRLSS-C-Q----------
----AE-RT----------------------PSIFGTAP-----------------EELTLS-YKAFNDACPYSKFAHLTANQAILEATE-------
KATR--------------IHIVDFG-IVHG-----IQWAAFLQALATRS--------AGK------PV-------------SVRISGI------------
--PSVVL--GNSPSASLLATGNRLRDFAKLLDLN-FEFEP-ILT-PVQ----------------------------EL-NG-------S-S-----FRV-
-DPD-----EILAVNFMLQLYNLL---DETNV-------
GVKTALSLAKSLNPSIVTLGEYEV-----------NL-N-DVGFLQRFK-------NALKYYSTIFESL-DP-S----LTRDSAE------
RVQVERLILGRRIAGAV-GL----DDGG-T-----R----RE-CMEDKEH----WKELMEG-AGFKPVTLSHYAMSQAKILLWNYNYSSS-------
FGLI------DSA-PG----------FLSLAWKDNPLLTVSSW
>StSCL4 .

--LLKSLVECARLAESE------PE-NVVKSLIR---L-RE----SVS-----QQGD--------P-MERVGFYFLEA-LYNGLSS-C-Q----------
----AE-RT----------------------PSIFGTTP-----------------EELTLS-YKAFNDACPYSKFAHLTANQAILEATE-------
KATR--------------IHIVDFG-IVHG-----IQWAAFLQALATRS--------AGK------PV-------------SVRISGI------------
--PSVVL--GNSPAASLLATGNRLRDFAKLLDLN-FEFEP-ILT-PVQ----------------------------EL-NG-------S-S-----FRI-
-DPD-----EILAVNFMLQLYNLL---DETNV-------
GVETALSLAKSLNPSIVTLGEYEV-----------NL-N-DVGFLQRFK-------NALKYYSTVFESL-DP-S----LTRDSPE------
RVQVERLILGRRIAGAV-GL----DDGG-T-----R----RE-CMEDKEH----WRELMEG-AGFKPVTLSHYAMSQAKILLWNYNYSSS-------
FGLI------DSA-PG----------FLSLAWKDNPLLTVSSW
>VvSCL4 .
--ILKALLDCARLADSE------PD-RAVKSLIR---L-RE----SVS-----EHGD--------P-TERVAFYFSEA-LYSRVSH---Q----------
----AE-KR----------------------PTLFETSS-----------------EEFTLS-YKALNDACPYSKFAHLTANQAILEATE-------
RARK--------------IHIVDFG-IVQG-----VQWAALLQALATRS--------AGK------PA-------------RIRISGI------------
--PAPAL--GKSPASSLFATGNRLRDFARLLDLN-FEFEP-ILT-PIQ----------------------------EL-NE-------S-T-----FRV-
-DPD-----EVLAVNFMLQLYNLL---DETPV-------
SVNAALRLAKSLNPKIMTLGEYEA-----------CL-N-EVDFINRFK-------NALRYYRAIFDSL-EP-N----LARDSSD------
RLQVERLLLGRRIAGVI-GP----EEPG-T-----R----RE-RMEDKEK----WKFLVES-CGFESVPLSHYAVSQAKILLWNYNYSSL-------
YAII------ESA-PG----------FLSLAWNKVPLLTVSSW
>CpSCL4 .
--LFKALLDCARLAESE------PD-RAVKSLIC---L-RE----SVS-----DQGN--------P-TERVGFYFTEA-LHSRLSL---Q----------
----AE-KS----------------------LTIVETSC-----------------EDFTLS-YKALNDACPYAKFAHLTANQAILEATE-------
TASM--------------IHIVDFG-IVQG-----VQWAALLQALATRT--------AGK------PA-------------RIRISGI------------
--PAPAL--GKSPGSSLLATGNRLRDFAKLLDLN-FEFEP-ILS-PIQ----------------------------EL-NE-------A-T-----FRV-
-EPD-----EVLAVNFMLQLYNLL---DETTA-------
AVEAALRLAKSLKPKIVTLGEHEA-----------SL-N-RVGFASRFN-------NALRFYAAVFESL-EP-N----LTRDSPE------
RLQVERLLLGRRISGVI-GR----EEPE-T-----R----RE-RMEDKEQ----WRVLMES-CGFDSVPLSHYAMSQAKILLWNYNYSPL-------
YSLI------DSE-PG----------FLSLAWNEVPLLTVSSW
>RcSCL4 .
---LKALLECARLAESE------PE-RAVKSLIK---L-RE----SVC-----EHGD--------P-TERVAFYFTEA-LYSRLCL---Q----------
----AE-KS----------------------LAMFETSS----------------EEDFTLS-YKALNDACPYSKFAHLTANQAILEATE-------
GASK--------------IHIVDFG-IVQG-----VQWAALLQALATRS--------AGR------PL-------------SIRISGI------------
--PAPVL--GKSPAASLLATGNRLGDFAKLLDLN-FEFVP-ILT-PIQ----------------------------EL-NE-------S-N-----FRV-
-DPD-----EVLAVNFMLQLYNLL---DETYG-------
AVETALKMAKSLNPEIVTLGEYEA-----------SL-N-QIGYENRFK-------NALRYYSAVFESL-EP-N----LSRDSTE------
RLQVERLLLGRRIAGAV-GP----EEAG-T-----R----RE-RIEDKEQ----WRILMES-CGFESVALSHYAMSQAKILLWNYNYSSL-------
YSLV------ESQ-PG----------FLSLAWNEVPLLTVSSW
>MeSCL4-1 .
--LLKALLECARLAESE------PD-KAVKSLVK---V-RE----SVS-----EQGD--------P-TERVAFYFTEA-LYSRVSQ---Q----------
----TE-RS----------------------LTMYETTS-----------------EDFTLS-YKALNDACPYSKFAHLTANQAILEATE-------
RATK--------------IHIVDFG-IVQG-----VQWAALLQALATRS--------AGK------PA-------------RIRISGI------------
--PATVL--GKSPAASLFATGNRLRDFSKLLDLN-FEFEP-ILT-PIN----------------------------EL-NE-------S-C-----FRI-
-DPD-----EVLAVNFMLQLYNLL---DETPV-------
AVETALRMAKSLNATIVTLGEYEA-----------SL-N-RIGFVNRFK-------NALRYYSAVFESL-EP-N----LSRDSPQ------
RLQVEKFLLGRRIAGII-GP----EEAG-T-----R----RE-CIEDKEQ----WRILMES-CGFESVALSNYAMSQAKILLWNYNYSSL-------
YSLI------ESQ-PG----------FLSLAWKEVPLLTVSSW
>MeSCL4-2 .
--LLKALLECAILAESE------PD-KAVKSLIK---M-RE----SVS-----EQGD--------P-TERVAFYFTEA-LYSRVSL---Q----------
----AE-KS----------------------LTIVETTC-----------------EEFTLS-YKTLNDACPYSKFAHLTANQAILEATE-------
SAGK--------------IHIVDFG-IVQG-----VQWAALLQALATRS--------TGK------PA-------------RIRISGI------------
--PAPVL--GKSPAASLFATGNRLRDFAKLLDLN-FEFEP-ILN-PIQ----------------------------EL-NE-------S-S-----FRV-
-DPD-----EVLAVNFMLQLYNLL---AETPV-------
AVETALRMAKSLNPRIVTLGEYEA-----------SL-N-QIGYVNRFK-------NALRYFSSVFESL-EP-N----LSRDSPE------
RLQVEKLLLGRRIAGVI-GP----EEAG-S-----R----RE-RIEDKEQ----WRVLMES-CGFESVALSHYAMSQAKILLWNYNYSSL-------
YSLI------ESQ-PG----------FLSLAWKEVPLLTVSSW
>TcSCL4 .
--LLKALLDCARLAESE------PE-RAIKSLIR---L-RE----SIS-----ECGD--------P-TERVAFYFTEA-LYSRVSR---Q----------
----AE-KR----------------------LTILDTTS-----------------EEFTLS-YKALNDACPYSKFAHLTANQAILEATE-------
RASK--------------IHIVDFG-IVQG-----VQWAALLQALATRS--------AGK------PT-------------QIRISGI------------
--PAPVL--GKSPASSLYATGNRLRDFAKLLDLN-FEFEP-ILT-PIE----------------------------EL-NE-------L-S-----FRV-
-DQD-----EVLAVNFMLQLYNLL---DEAPV-------
TVETALLLAKSLNPKIVTLGEYEA-----------SL-N-RVGFENRFK-------NALRYYSAVFESL-EP-N----LPRDSPE------
RIQVERLLLGRRIAGVI-GP----EEVE-K-----R----RE-RMEDKEQ----WKNLLES-AGFETVALSHYARSQARILLWNYNYSSS-------
YSLI------ESQ-PG----------FLSLAWNEVPLLTVSSW
>GrSCL4-1 .
--LLKALLDCARLSDSE------PE-RAIKSLVE---L-RE----SVS-----ERGD--------P-TERVAFYFTQA-LYSRVSL---P----------
----AE-KR----------------------LNLLETTS-----------------EDFTLS-YKVLNDACPYSKFAHLTANQAILEATE-------
RATK--------------IHIVDFG-IVQG-----VQWAALLQALAARS--------AGK------PT-------------RIRISGI------------
--PAPVL--GSSPAPSLYATGNRLRDFAKLLDLN-FEFEP-ILT-PIK----------------------------EL-KE-------S-C-----FRV-
-DND-----EVLAVNFMLQLYNLL---DATPV-------
TVEAALCLAKSLNPKIVTLGEYEA-----------SL-N-RVGFVNRFK-------NALRYYTAVFESL-EP-N----LPRDSPE------
RVEVERQLLGGKIGAII-GA----EEAE-K-----R----RE-RMEDKEQ----WKVLMES-AGFETVSLSHYAISQAKILLWNYNYSSS-------
YSLI------ESK-PG----------FLTLAWKEVPLLTVSSW
>GrSCL4-2 .
--LLKALLDCATLADSE------PE-RAIKSLAK---L-KE----SVS-----EHGD--------P-TERVAFYFTEA-LHSRLSH---Q----------
----TD-QR----------------------LTMIETTS-----------------EDFTLS-YKALNDACPYSKFAHLTANQAILEATD-------
RASK--------------IHIVDFG-IVQG-----VQWAALLQALATRS--------AGK------PT-------------RIRISGI------------
--PAPVL--GSTPAPSLYATGNRLRDFSKLLDLN-FEFEP-ILT-PVT----------------------------EL-TG-------S-S-----FRV-
-DED-----EVLAVNFMLQLYNLL---DSTPA-------
AVETALALAKSLNPKIVTLGEYEA-----------SL-N-RVGFLNRFK-------NALRYYSAVFESL-EP-N----LPRDSPE------

RIQVEKHLLGRKVAGVI-GR----EEAG-T-----Q----RE-RMEDKEQ----WKILMEI-SGFETVTLSHYAISQAKILLWNYNYSSS-------
YSLI------ESQ-PG----------FLSLAWNEVPLLTVSSW
>FvSCL4 .
--LLKALLDCARVIESE------PE-RAIKSLVR---L-RE----SVS-----ERGD--------P-TERVTFRFTEA-LYSRVSP---P----------
----PD-KT----------------------SAAYDGSC----------------EEDFTLS-YKALNDACPYSKFAHLTANQAILEATE-------
RATK--------------IHIVDFG-IVQG-----VQWAALLQALATRS--------TGK------PS-------------KIRISGI------------
--PAPSL--GNSPAASLFATGNRLCEFAKLLELN-FEFEP-VLT-PIQ----------------------------EL-NE-------S-C-----FRV-
-DPD-----EFLAVNLMLQLHNLL---DESTT-------
GVEAALTLAKLLNPKIVTLGEYEA-----------KL-N-RVGFMSRFK-------TALKYYKAMFESL-EP-N----MARDSPE------
RLQVERLLLGRRIASVV-GA----EESG-T-----K----RQ-RLEDKEQ----WKFLMES-AGFEPVTLSHYAVSQAKILLWNYSYNAS-------
YSLI------ESP-PG----------FLSLAWKEVPLFTVSSW
>MdSCL4-1 .
--LLKALLDCARLAESD------PD-GAVKSLVR---L-RE----SIS-----DHGD--------P-TQRVAFYFAEA-LQNRVSF-L-Q----------
----SE-KS------------F---------TTAHDTPC-----------------EDFTLS-YKALNDACPYSKFAHLTANQAILEATE-------
RATK--------------LHIVDFG-IVQG-----VQWAALLQALATRS--------TGK------PV-------------SIRISGI------------
--PAPSL--GDSPAASLIATGNRLREFAXLLELN-FEFEP-ILT-PVH----------------------------QL-DE-------S-C-----VRV-
-DPD-----EALAVNLVLQLYNLL---DEKPT-------
AVQSALKLAKSLNPQIVTLGEYEA-----------NL-N-RVGFASRFK-------NALKYYAALFESL-EP-N----MIRDSPE------
RLKVERLLLGRRIGSLV-GP----EQPG-T-----K----RE-RFEDKEQ----WKYLMEC-SGFEPVALSXYSVSQAKILLWNYNYSSL-------
YSLS------ESP-PG----------FLSLSWNEVPLFTVSSW
>MdSCL4-2 .
--LLKALLDCARLTESD------TD-RAVKSLIR---L-RE----SIS-----DHGD--------P-TERVAFYFAEA-LHNRVSV-L-Q----------
----SE-KT------------F---------TTAYDTPC-----------------EDFTLS-YKALNDACPYSKFAHLTANQAILEATE-------
RATK--------------IHIVDFG-IVQG-----VQWAALLQALATRS--------TGK------PV-------------SIRISGI------------
--PAPSL--GDSPAASLFATGNRLREFAKLLELN-FEFEP-ILT-SVN----------------------------EL-DE-------S-C-----FRF-
-EPE-----EALAVNFMLQLYNLL---DDKPA-------
AVHSALKLAKSLNPQIVTLGEYEA-----------NL-N-RVEFASRFK-------NALKYYTALFESL-EP-N----LTRDSPE------
RLKVERLLLGRRIGGLV-GP----EQSG-T-----K----RE-RFEDKEQ----WKYLMES-SGFESVALSHYSVSQAKILLWNYNYSSL-------
YSLK------ESP-PG----------FLSLSWSEVPLFTVSSW
>PpSCL4 .
--LLKALLDCARLAESD------PD-RAVKSLIR---L-RE----SVS-----DRGD--------P-TERVGFYFTEA-LQSRVSS-L-Q----------
----SE-KSLA-------------------ATTTYDTAC-----------------EDFTLS-YKALNDACPYSKFAHLTANQAILEATE-------
RATK--------------IHIVDFG-IVQG-----VQWAALLQALATRS--------TGK------PS-------------RIRISGI------------
--PAPSL--GTSPAASLFATGNRLRDFAKLLELN-FEFEP-ILT-PVH----------------------------EL-DE-------S-C-----FRV-
-EPD-----EALAVNLMLQLYNLL---DETPT-------
AVQSALKLAKSLNPKIVTLGEYEA-----------NL-S-RVGFTSRFK-------NALKYYTALFESL-EP-N----MTRDSPE------
RLKVEKLLLGRRIGGVV-GP----EQPG-T-----K----RE-RFEDKEQ----WKYLMES-SGFEPVALSHYAVSQAKILLWNYN-NSL-------
YSLI------ESP-PG----------FLSLAWNEVPLFTVSSW
>LuSCL4-1 .
--LLQSLLECARLSESE------TE-SAVKSLVK---I-RV----SAS-----EHGN--------P-TERVAYYFSEA-LYNRLSK---Q----------
----AG-ES----------------------FTMFETTS-----------------EEFTLS-YKSLNDACPYSKFAHLTANQAILEATE-------
TATK--------------IHIVDFG-VVQG-----VQWAALLQALATRS--------AGK------PV-------------LVRISGI------------
--PAPAL--GSSPAASLFATGKRLLDFAKLLDLN-FEFEP-ILT-PIQ----------------------------DL-NE-------S-C-----FRV-
-EPD-----EVLTVNFMLQLFNLL---DETTA-------
TVETALRMAKSLNPRIVTLGEYEV-----------SL-N-RIGYLTRFN-------NALKYYSAVFESL-EP-S----LSRDSTE------
RLEVERMLLGRRIAGVI-GP----EEPGVV-----Q----RE-RMEDREQ----WRLLMEK-CGFESVALSNYAMSQAKILLWNYNYSPR-------
YSLV------ESK-PG----------FVSLAWKEVPLLTVSSW
>LuSCL4-2 .
--LLKSLLECARLSESE------TE-SAVKSLVK---I-RE----SVS-----EHGD--------P-TERVAYYFTEA-LYNRLSK---Q----------
----AG-EN----------------------FIMFETTS-----------------DEFTLS-YKSLNDACPYSKFAHLTANQAILEATE-------
TAAK--------------IHIVDFG-VVQG-----VQWAALLQALATRS--------AGK------PA-------------LVRISGI------------
--PAPAL--GSSPAASLFATGKRLLDFAKLLDLN-FEFEP-ILT-PTQ----------------------------DL-NE-------S-C-----FRV-
-EPD-----EVLAVNFMLQLFNLL---DESTA-------
TVETALRMAKSLNPRIVTLGEYEV-----------SL-N-RIGYLTRFN-------NALRYYSAVFESL-EP-S----LSRDSTE------
RLEVERMLLGRRIAGVI-GP----EEPGVV-----K----RE-RMEDREQ----WRLLMEM-CGFEPVALSNYAMSQAKILLWNYNYSPR-------
YSLV------ESK-PG----------FISLAWKEVPLLTVSSW
>PtSCL4-1 .
--VLKALVECAQLVESK------AD-QAVKSLVK---C-KD----LVS-----ENGD--------P-VERAGFYFAEG-LCRRVAV-G-E----------
----LD-VL----------------------KNFDQ-TS-----------------EEFTLS-YKALNDACPYSKFAHLTANQAILEATE-------
KASK--------------IHIVDFG-IVHG-----VQWAALLQALATRS--------AGK------PV-------------RIRISGI------------
--PAPVL--GKNPAASLLATGNRLLDYAKLLGLN-FEFEP-ILT-PIQ----------------------------EL-NE-------S-C-----FRA-
-EPD-----EVLAVNFMLQLYNLL---DESPV-------
AVETALKMAKSLNPIIVTLGEYEA-----------SL-N-RVGYLTRFK-------NALRYYTAVFESL-EP-N----LSRDSPE------
RLQVERLLLGQRISSVV-GP----EEPG-M-----R----RE-RMEDKDQ----WGVLMES-SGFESVSLSHYAMSQAKILLWYCNYSDL-------
YSLD------DSQ-PG----------FLTLAWNEVPLLTVSSW
>PtSCL4-2 .
--VLKALVECAQLVESK------AD-QAVKSLIR---F-KE----SVS-----ENGD--------P-GERVGFYFVKG-LCRRVAV-G-E----------
----LD-DL----------------------KNFHQTTS-----------------EEFTLC-YKALNDACPYSKFAHLTANQAILEATE-------
KASK--------------IHIVDFG-IVQG-----IQWAALLQALATRS--------AGK------PV-------------RIRISGI------------
--PAPVL--GKNPAASLLATGNRLLDFAKLLDLN-FEFEP-ILT-PIQ----------------------------EL-NE-------S-C-----FRV-
-EPD-----EVLAVNFMLQLYNLL---GETPG-------
AVETALKMAKSLNPRIVTLGEYEV-----------SL-N-RVGYLTRFK-------NALRYYTAVFESL-DP-N----MSRDSQE------
RLQVERLLLGRRISGVV-GP----D--G-I-----R----RE-RMEDKEQ----WRVLMES-SGFESVSLSHYAMSQAKILLWNYNSSTM-------
YSLD------DSQ-PG----------FLTLAWNEVPLLTVSSW
>CsSCL4-1 .
--VLKVLLDCARLCDSE------PN-RAAKTLNR---I-SK----SLR-----EDGD--------P-IERVGFYFGDA-LRKRLSS-T-P----------
----MK-NCLD--------------------STESDANS-----------------EDFLLS-YKALNDACPYSKFAHLTANQAILEVTE-------
RASK--------------IHIVDFG-IVQG-----VQWAALLQALATRA--------TGK------PV-------------RVRISGI------------

```
--PAPSL--GDSPAASLYATGNRLSEFAKLLELN-FEFQP-ILT-PIE----------------------------NL-KE-------S-S-----FSV-
-QSD-----EVLAVNFMLQLYNLL-----------------------------------------------------DENPT-------
GVHNALRLAKSLSPHIVTLGEYEA-----------SL-N-RNGFYNRFK-------NALKFYSAIFESL-EP-N----LPRNSPE------
RLQLERLLLGRRIAGVV-GTV---EDSRRE-----R----RV-RMEDKEQ----WKNLMEN-TGFEPVALSHYAISQAKILLWNYNYSSL-------
YTLI------ESA-PE----------FLSLAWNDVPLLTVSSW
>CsSCL4-2 .
--LLKTLIECARISESE------PD-RAAQTLIK---L-KE----SSS-----EHGD--------P-TERVAFYFMDA-LCRRLS---------------
----------------------------------------------------------------------------------------------------
----------------------------------------------------------------------------------------------------
----------------------------------------------------------------------------------------------------
----------------------------------------------------------------------------------------------------
----------------------------------------------------------------LPSDS-------------RLI---------------
----------------------------------------------------------------------------------------------------
-----------------
>GmSCL4-1 .
--LLKALSECASLSETE------PD-QAAESLSR---L-RK----SVS-----QHGN--------P-TERVGFYFWQA-LSRKMWG---D----------
----KE-KM--------------------------EPSS----------------WEELTLS-YKALNDACPYSKFAHLTANQAILEATE-------
NASN--------------IHILDFG-IVQG-----IQWAALLQAFATRA--------SGK------PN-------------KITISGI------------
--PAVSL--GPSPGPSLSATGNRLSDFARLLDLN-FVFTP-ILT-PIH----------------------------QL-DH-------N-S-----FCI-
-DPN-----EVLAVNFMLQLYNLL-----------------------------------------------------DEPPS-------
AVDTALRLAKSLNPRIVTLGEYEA-----------SV-T-RVGFVNRFR-------TAFKYFSAVFESL-EP-N----LAADSPE------
RFQVESLLLGRRIAAVI-GP-------GPV----------RE-SMEDKEQ----WRVLMER-AGFESVSLSHYAISQAKILLWNYSYSSL-------
FSLV------ESKPPG----------FLSLAWKDVPLLTVSSW
>GmSCL4-2 .
----------------------------------------------------------------------------------------------------
---------------------------------------------------------------------------FDQLTANQAILEATQ-------
TASN--------------IHIVDFG-IVQG-----IQWAALLQAFATRP--------SGK------PN-------------KIRISGI------------
--PALSL--GSSPGPSLSATAHRLSDFAKLLDLN-FHFTP-ILT-PIH----------------------------QL-DR-------N-S-----FCI-
DDTN-----EALAVNFMLQLYNLL-----------------------------------------------------DEPPT-------
AVDTALRLAKSLNPKIVTLGEYEA-----------SV-T-RFGFVNRFK-------TAFKYFSAVFESL-EP-N----LAADSPE------
RFQVESLLLGRRIAAVIGGP-------GSV-----R----RE-SMEDKEQ----WRVLMER-AGFESVSLSHYAISQAKILLWNYSYSSL-------
FSLV------ESTPPG----------FLSLAWKDVPLLTVSSW
>PvSCL4 .
--LLKALTECASFAESE------PD-QAAESLAR---L-RK----SVS-----QHGN--------P-TQRVGFYFCEA-LSRKIWA---E----------
----KE-KA----------------EPTSTSTTTTTTTS-----------------EELTLS-YKAMNDACPYSKFAHLTANQAILEATD-------
GAAN--------------IHILDFG-IVQG-----IQWAALLQAFATRS--------SGK------PN-------------KIKISGI------------
--PAIAL--GASPGPSLSATGNRLSEFAKLLDLN-FEFTP-ILT-PIH----------------------------HL-DH-------N-S-----FCI-
-DPD-----EALAVNFMLQLYNLL-----------------------------------------------------DEPPT-------
AVDTALRLAKSLNPKIVTLGEYEA-----------SL-T-RVGFLNRFK-------TAFNYFSAVFESL-DP-N----LPADSPE------
RFQVESLLLGRRIDAVI-GP-------GPA----------RE-SMEDREQ----WRVLMER-AGFESVSLSHYAVSQAKILLWNYGYSSL-------
FSLL------ESTPPG----------FLSLAWKDVPLLTLSSW
>MtSCL4 .
--LLKTLTEIASLIETQK-----PN-QAIETLTH---L-NK----SIS-----QNGN--------P-NQRVSFYFSQA-LTNKITA---Q----------
-------SS--------------------IASSNSSSTT----------------WEELTLS-YKALNDACPYSKFAHLTANQAILEATE-------
GSNN--------------IHIVDFG-IVQG-----IQWAALLQAFATRS--------SGK------PN-------------SVRISGI------------
--PAMAL--GTSPVSSISATGNRLSEFAKLLGLN-FEFTP-ILT-PIE----------------------------LL-DE-------S-S-----FCI-
-QPD-----EALAVNFMLQLYNLL-----------------------------------------------------DENTN-------
SVEKALRLAKSLNPKIVTLGEYEA-----------SLTT-RVGFVERFE-------TAFNYFAAFFESL-EP-N----MALDSPE------
RFQVESLLLGRRIDGVI-GV--------------------RE-RMEDKEQ----WKVLMEN-CGFESVGLSHYAISQAKILLWNYSYSSL-------
YSLV------ESQ-PA----------FLSLAWKDVPLLTVSSW
>CcSCL4 .
--LTKALIDCARLVESE------PD-KAVKSLVR---L-RG----SVC-----AHGN--------P-TERVAYYFTEA-LYKRLTQ---R----------
----AE-KS----------------------ITTLEANC-----------------EDCILS-FKTLNDACPYSKFAYLTANQAILEATE-------
NASH--------------IHIVDFG-IVQG-----IQWSFLLQALANRP--------TGK------PV-------------KVRISGI------------
--PAPAL--GKSPAASLLATGDRLREFAGSLSLN-LEFEP-ILI-PIR----------------------------KL-RA-------S-S-----FRV-
-DPN-----EALVVNFMLQLNSLL-----------------------------------------------------DDNRL-------
AVENALQMAKSLNPNVVTLAEYEA-----------NL-N-RTGFLARFK-------NALKYYTAVFESL-EP-N----MTTDSDE------
RFQVERQILGPRIANLL-AP----EKQG-A-----K----RE-RVEDIEN----WRIFMEN-SDFEGIPFSHYALSQAEILLWNYNYSPL-------
FTLN------QSH-DN----------LLTLSWKKVPLLTVSSW
>CsiSCL4 .
--LTKALIDCACLVESE------PD-KAVKSLVR---L-RG----SVC-----AHGN--------P-TERVAYYFTEA-LYKRLTQ---R----------
----AE-KS----------------------ITTLEANC-----------------EDCILS-FKTLNDACPYSKFAYLTANQAILEATE-------
NASH--------------IHIVDFG-IVQG-----IQWSFLLQALANRP--------TGK------PV-------------KVRISGI------------
--PAPAL--GKSPAASLLATGDRLREFAGSLSLN-LEFEP-ILI-PIR----------------------------KL-RA-------S-S-----FRV-
-DPN-----EALVVNFMLQLNSLL-----------------------------------------------------DDNRL-------
AVENALQMAKSLNPIVVTLAEYEA-----------NL-N-RTGFLARFK-------NALKYYTAVFESL-EP-N----MTTDSDE------
RFQVERQILGPRIANLL-AP----EKQG-A-----K----RE-RVEDIEN----WRIFMEN-SDFEGIPFSHYALSQAEILLWNYNYSPL-------
FTLN------QSH-DN----------LLTLSWKKVPLLTVSSW
>EgSCL4 .
--LLKALLDCARVSESD------PE-LAAKTLAR---L-RE----SAS-----ERGD--------P-AERVAFYFSDA-LHSRLSL-D------------
------------------------------GSPIASSTS----------------PEEFTLS-YKALNDACPYSKFAHLTANQAILEATE-------
KASK--------------IHIVDFG-IVQG-----VQWAALLQALATRS--------TGK------PD-------------RIRISGI------------
--PARAL--GENPAPSLLATGNRLREFAKLLDLN-FEFVP-ILD-PTD----------------------------SL-DA-------A-S-----FRV-
-DPD-----EDLAVNFMLQLYNLL-----------------------------------------------------GETSA-------
AVDAALRLSKSLGPKIVTLGEYEA-----------SL-N-RAPYPARFK-------NALAYYAAVFESL-EP------MSRDSLQ------
RREVERLLLGRRIVRSV-GPEAP-PGTG-A-----E----RE-RMEDKEQ----WRALMER-AGFEAVPLSHYAVSQAKILLWNYKYSEL-------
YSLQ------DSP-PG----------FLSLAWNKAPLLTVSAW
>AcSCL4-1 .
```

CSLLKSLLLCARISDSE------PE-KAIKTLNR---L-RE----SVS-----ERGD--------S-TERVAFYFSEA-LFSRISS---K---------
----SL-KN----------------------FPTFDSNS-----------------EEFLLS-YKALNDACPYSKFAHLTANQAILEATE-------
SANQ-------------IHIVDFG-IVQG-----LQWAALLQALATRS--------SGK------PT------------LIRISGI------------
--PAPAL--GKSPAASLIATGNRLREFAELLELN-IQFEP-ILT-PIE----------------------------EL-NE-------S-S-----FRV-
-NSD-----EVLAVNFMLQLYNLL--GETTL-------
AVEKALKLAKSLNPKVLTLGEYEV-----------SL-N-RVEYYDRFK-------NAMKYYSAVFESL-EP-S----LERDSED------
RLQVERLILGRRIMSVV-GP----GEGS-M-----R----RE-RMEEKEE----WKNLIES-CGFEAIAVSHYAVSQADILLWNYNYSSK-------
YSLV------ESP-PG----------FLSLCWNGTPLLTVSSW
>AcSCL4-2 .
CSLLKSLLLCARISDSE------PE-KAIKTLNR---L-RE----SVS-----ERGD--------S-TERVAFYFSEA-LFSRISS---K---------
----SL-KN----------------------FPTFDSNS-----------------EEFLLS-YKALNDACPYSKFAHLTANQAILKATE-------
SANQ-------------IHIVDFG-IVQG-----LQWAALLQALATRS--------SGK------PT------------LIRISGI------------
--PAPAL--GKSPAASLIATGNRLREFAELLELN-IQFEP-ILT-PIE----------------------------EL-NE-------S-S-----FRV-
-NSD-----EVLAVNFMLQLYNLL--GEITL-------
AVEKALKLAKSLNPKVLTLGEYE---

>MaSCL4-1 .
--LLESLLDCARIADSD------PD-LAAKSLIH---V-RE----SAS-----ESGD--------P-TERVAFYFAEA-LYRRLHG-------------
----AQRKHHS-------------YPSTAAAPPSCSSSS------------FDSSPEDFTLC-YKALNDACPYSKFAHLTANQAILEATE-------
SAAR-------------IHIVDFG-IMQG-----VQWAALLQALATRP--------SGK------PS------------RVRISGI------------
--PAPAL--GAAAAASLAATGNRLRDFAALLDLD-FEFDP-ILT-PIS----------------------------EL-SA-------S-T-----FRV-
-DPE-----ETLAVNFMLQLCHLL--GDSPE-------
PVERVLRVAKSLGPRVVTLGEYEA-----------SL-N-RAAFLERFR-------TALAYYAAVFDSL-DP-A----MQRDSAE------
RARVERLLFGRRILGVV-GPG---DEQ--I-----R----RE-RMEANAE----WRAVMEQ-CGFETVPLSNYAVSQAKLLLWNYNYSSK-------
YTLS------DSA-PV----------FLSLAWGDRPLLTVSSW
>MaSCL4-2 .
--LLESLLDCARLADGD------PD-LAAKSLIH---V-RE----SAS-----VLGD--------P-TERVAFYFAEA-LNRRLLG-------------
----DQ-KDHS-------------HPST-----------------------------------VAVFNDACPYSKFAHLTANQAIVEATE-------
SAAR-------------IHIVDFG-IIQG-----IQWAALLQALATRP--------RGK------PS------------RVRVSGI------------
--PAPML--GAAPAASLTATGNRLRDFAAILDLD-FEFDP-ILT-PIA----------------------------EL-TV-------S-C-----FRI-
-DSD-----EVVVVNFMLQLYHLL--ADSPE-------
SVERVLGIAKSLVPRVVTLGEYEA-----------SV-N-RGRFVERFK-------AALAYYAAVFDSL-DP-A----IRRDSAE------
RAQMERVLLGPRILGAV-GAG---DGPN-------R----RE-RMEAKEE----WRAVMER-CGFEPVPVSNFAVSQAKLLLWNYDYSYK-------
YAVL------DSA-PG----------FLTLAWGDRPLLTVSSW
>MaSCL4-3 .
--LLESLLQCARLADSD------PH-LATKSLIH---V-RE----SAS-----ELGD--------P-TERVAFYFAEA-LHRRLLG-------------
----AQRKH--------------PLPTSAPQPPSLDSSP-----------------EEFTLC-YKVLNDACPYFKFAQLTANQAIIEATE-------
SAGR-------------IHIVDFG-IVQG-----VQWPSLLQALATRP--------SGK------PS------------KVRISGI------------
--PAPALG-GAAIATSLAATGNRLRDFAALLDLD-FEFDC-ILT-PIP----------------------------EI-AA-------S-T-----FRV-
-DPD-----ELVVVNFMLQLSQLL--GDSPE-------
PVERVLRVAKSLGPRILTLAEYEA-----------SL-N-HAGFVDRFG-------AALGHYAAVFESL-DP-A----MGRDAVE------
RARMERVLIGHRILEAV-GPF---EGQN-------R----RV-RMAPKEE----WRAVMER-CGFSSVPLSNYAVSQAKLLLWNYNYSSR-------
YTLL------DSP-PP----------CLTLAWGDRPLLTVSSW
>PaSCL4 .
--LLHSLLDCAKIVDTE------PE-RAGQSVAY---L-QS----IAS-----QHGD--------P-TQRIVFHFADA-LAKRLSK-G-----------
----IE-QK------------------P--QFQSSDECS--------------KSFEGLTLA-YKALNDACPYSKFAQLTGNQAILEAMD-------
KAEK-------------IHIVDFG-IVQG-----VQWAALLHAFATRP--------GGK------PQ------------KIKITGI------------
--PAPTL--GQNPTSSLLATGKRLTEFAKLLDLE-FEFCP-VSK-HMS----------------------------EV-EL-------S-T-----LKI-
-EQD-----ECIAVNFMLQLYNLL--GDSPE-------
PLVKILKLAHALSPKVVTLGEYEA-----------YL-N-ACHFQVRFR-------NALDYFSAFFDSM-EPTN----MSRDCAE------
RLNVEKLFFAEKIMGIV-------AFEGAE-----R----KT-RLEGRDQ----WRILMES-AGFKFTNLSHYAKSQARILL--YNYCEG-------
YSLQ------ESS--G----------FLSLAWQTRPLLTVSAW
>AtriSCL4 .
--LLLSLVDAARTVHSD------PG-HAAASLAE---L-RR----SAP-----DSGS--------P-TERLVSYFAEA-LIERLDP-T------------
------------------------------GHPVAQFSA-----------------EESTLC-YKALNDACPFSKFAHLTANQAILEAME-------
TADS-------------IHIVDFG-IVQG-----VQWAALLQALATRP--------AGK------PA------------KIRISGI------------
--PAPSL--GSNPAPSLAATGNRLCDFAKLLELN-FEFSP-VPC-SIE----------------------------DV-ME-------G-D-----LRR-
-ADG-----EFVAVNFMLQLHRLV--DEKME-------
KVARALRVAKALKPKVVTLGEYEL-----------GL-N-RGDFMGRLS-------RALRYYGAMFECL-EA-G----MGRACKE------
RERVERVVIGRRIAGAV------------K-----N----TE-RMEERCI----WREEIER-AGFVGLGLSHYAVSQARMLLWNYDYSSS-------
YSLI------EAG-PG----------FLTLAWKDQPLLTVSAW
>PpatSCL4 .
VHLVHLLLECATQIEKN------QH-LAVSTLCR---L-RD----LSS-----PLGD--------P-MQRVAAYFCDA-LTKRIAR-G-K----------
----GE-AD------------------P-GVLEAPHNSPK--------------------A-CQVLNEACPYMKFAHLTANQAILEAVK-------
GCES-------------VHILDFG-ITHG-----IQWAALLQAFASLP--------KKQP-----PP------------KVRITGIS----------
VNNPA-----SESASLSVLATGKRLQSFAEHLNVE-FEFCPVILV-SME----------------------------DF-TP-------E-S-----
IQL--NPD-----EKTVANFMLQLHEML--DEEGS------
PSILRLLRSVISLSPALVTLTEHDA-----------AL-N-RPEFRPRFM-------DALHFYCALFDSL-DS-T----MPRDCHD------
RLNVENNYFAKQIENIV-------ANEGVD-----R----TE-RYECTET----WIRIMET-VGFTLVPLSHYAYSQAQQLLWQF--CDS-------
FRLQ------RPS--G----------CIALAWQDRSLITVSAW
>SmSCL4-1 .
-QLVQLLVSCLDDMESGQ-----AS-PAMDKLAT---L-KA----MAS-----SSGN--------A-VEKCAWYFSSG-LEARLHR-RGG---------
----DD-HS---------------DG-DDEEEESPSSSP--------------NKAEAIAMA-YKTLTDACPYLKFAHLTANQALLEATD-------
GAPK-------------IHIVDYG-TMQG-----VQWAAFLQAFATWP--------AKNP----SPR------------SLRITGI------------
--PSPHL--GSNPAPAMLATQRRLTDFAKLLGVD-FQFCP-ILE-PIR----------------------------DF-QP-------SQS-----LRT-
-DPD-----EVVAVNFVLQLAQL---PA------
PALKRAFSLVQRLNPRIVTVAEYEA-----------N--N-GASLRDQLA-------SNARFYSSVFESL-DV-A----LPGDDPQ------

RITAERLFFGREITKSL-----V---EG-T-----N----CE-CPEKQRE----WQRCIDG-AGLWSAALSHYTVSQARLLLWLYNKSEN-------
FTLL------QG--PG----------SLSLGWLGTSIVTVSAW
>SmSCL4-2 .
-QLVQLLVSCLDDMESGQ-----AS-SAMDKLAT---L-KA----MAS-----SSGN--------A-VEKCAWYFSSG-LEARLHR-RGG----------
----ND-HS---------------DGDDDEEEESPSSSP--------------NKAEAIAMA-YKTLTDACPYLKFAHLTANQALLEATD-------
GAPK--------------IHIVDYG-TMQG-----VQWAAFLQAFATWP--------AKNP----SPR-------------SLRITGI------------
--PSPHL--GSNPAPAMLATQRRLTDFAKLLGVD-FQFCP-ILE-PIR----------------------------DF-QP-------SQS-----LRT-
-DPD-----EVVAVNFVLQLAQL---PA------
PALKRAFSLVQRLNPRIVTVAEYEA-----------N--N-GASLRDQLA-------SNARFYSSVFESL-DV-A----LPGDDAQ------
RITAERLFFGREITKSL-----V---EG-T-----N----CE-CPEKQRE----WQRCIDG-AGLWSAALSHYTVSQARLLLWLYNKSEN-------
FTLL------QG--PG----------SLSLGWLGTSIVTVSAW
>OsGRAS-28 .
VRMIALLMECAAAMSVGN-----LA-GANGALLE---L-SQ----MAS-----PYAA--------SCGERLVAYFARA-MAARLVG-SWV----------
----------------------------GVVAPMAPPS-----------------CGAINAA-FRALYNVAPFARLAYLACNQAILEAFH-------
GKRL--------------VHIVDLD-VVPGG---ALQWLSLLPALAARP--------GGP------P--------------VIRVTGF------------
---------GMS-ASVLHDTGNQLAGLARKLCMF-FEFYA-VAK-RPG--------DADAVA--------------DM---------PG-R---------
-RPG-----EAVAVHWLR--HAMY---DA--AGDDGAS------------
MRLVRWLEPAAVTLVEQER--AHGGGG------G-HGRFLDRFV-------SALHHYSAVFDAM-GA-SR---PDGEDAS------
RHLAEHGVLGREIANVL-AV----GGP--A-----R----SS-GREGPGS----WREVLAR-HGFAHAGGGGGGRAQ---LVAAACPGGLG------
YTVAGDH-------DG----------TVRLGWKGTPLYAVSAW
>OsGRAS-1 .
IRLVHLLMSCAGAIEAGD-----HA-LASAQLAD-----SHAALAAVS-----AASG----------IGRVAVHFTTA-LSRRLFP-S------------
----------------------------PVAPPTTDAEHAF------L---------------YHHFYEACPYLKFAHFTANQAILEAFH-------
GCDH--------------VHVIDFS-LMQG-----LQWPALIQALALRP--------GGP------P--------------FLRITGI------------
GPPSP-T--GRD---ELRDVGLRLADLARSVRVR-FSFRGVAAN-SLD----------------------------EV-RP-------W-M-----LQI-
-APG-----EAVAFNSVLQLHRLL---GDPAD-----
QAPIDAVLDCVASVRPKIFTVIEQEA-----------DH-N-KTGFLDRFT-------EALFYYSAVFDSL-DA------ASASGGA------
GNAMAEAYLQREICDIV-CG----EGA--A-----R----RE-RHEPLSR----WRDRLTR-AGLSAVPLGSNALRQARMLVGLF-SGEG-------
HSV-------EEA-DG----------CLTLGWHGRPLFSASAW
>AtRGA .
VRLVHALMACAEAIQQNN-----LT-LAEALVKQIGCL-------AVS-----QAGA----------MRKVATYFAEA-LARRIYR--------------
-----------------------------LSPPQNQIDHCLSD---TL---------------QMHFYETCPYLKFAHFTANQAILEAFE-------
GKKR--------------VHVIDFS-MNQG-----LQWPALMQALALRE--------GGP------P--------------TFRLTGI------------
GPPAPDN------SDHLHEVGCKLAQLAEAIHVE-FEYRGFVAN-SLA----------------------------DL-DA-------S-M-----LEL-
-RPSD---TEAVAVNSVFELHKLL---
GRPGGIEKVLGVVKQIKPVIFTVVEQES-----------NH-N-GPVFLDRFT-------ESLHYYSTLFDSL-EG------VPNSQDK--------
VMSEVYLGKQICNLV-AC----EGP--D-----R----VE-RHETLSQ----WGNRFGS-SGLAPAHLGSNAFKQASMLLSVFNSGQG-------YRV--
-----EES-NG----------CLMLGWHTRPLITTSAW
>OsGRAS-13 .
LRLLHLLMAAAEALSGPHK---SRE-LARVILVR---L-KE----MVSHTASANAAA--------SNMERLAAHFTDA-LQGLLDG-SHP----------
--------------------------VGGSGRQAAAAASHHH-------------AGDVLTA-FQMLQDMSPYMKFGHFTANQAILEAVS-------
GDRR--------------VHIVDYD-IAEG-----IQWASLMQAMTSRA--------DGVP----AP--------------HLRITAVSRS--------
GG--------G--GARAVQEAGRRLSAFAASIGQP-FSFGQCRLD-SDE----------------------------RF-RP-------A-T-----
VRM--VKG-----EALVANCVLHQAAAT--TTIRRPTG-------
SVASFLSGMAALGAKLVTVVEEEGEAEKDDDGDSAGDAA-AGGFVRQFM-------EELHRYSAVWDSL-EA-G----FPTQSRV------
RGLVERVILAPNIAGAV------------S-----R-A-YRGVDGEGRCG----WGQWMRG-SGFTAVPLSCFNHSQARLLLGLF--NDG-------
YTVE------ETG-PN----------KIVLGWKARRLMSASVW
>AtGRAS-23 .
LRLVHLLVAAADASTGANK---SRE-LTRVILAR---L-KD----LVS-----PGDR--------TNMERLAAHFTNG-LSKLLE---------------
-------------------------------RDSVLCPQQHRD--------DVYDQADVISA-FELLQNMSPYVNFGYLTATQAILEAVK-------
YERR--------------IHIVDYD-INEG-----VQWASLMQALVSRN--------TGPS----AQ--------------HLRITALSRA--------
TN--------GKKSVAAVQETGRRLTAFADSIGQP-FSYQHCKLD-TNA-----------------------------F-ST-------S-S-----
LKL--VRG-----EAVVINCMLHLPRFS---HQTPS-------
SVISFLSEAKTLNPKLVTLVHEE-----------VGLMG-NQGFLYRFM-------DLLHQFSAIFDSL-EA-G----LSIANPA------
RGFVERVFIGPWVANWL------------T-----RIT-ANDAEVESFAS----WPQWLET-NGFKPLEVSFTNRCQAKLLLSLF--NDG-------
FRVE------ELG-QN----------GLVLGWKSRRLVSASFW
>OsGRAS-6 .
LCLIHLLLNCAAAAAAGR-----LD-AANAALEH---I-AS----LAA-----PDGD--------A-MQRVAAAFAEA-LARRALR-AWP--GL---
CRALLL-------------------------PRASPTP---------------------AEVAAA-RRHFLDLCPFLRLAGAAANQSILEAME-------
SEKI--------------VHVIDLG-GADA-----TQWLELLHLLAARP--------EGP------P--------------HLRLTSV------------
---------HEH-KELLTQTAMALTKEAERLDVP-FQFNP-VVS-RLD----------------------------AL-DV-------E-S-----LRV-
-KTG-----EALAICSSLQLHCLLASDDD----------------------------AAA------VAGGDKERRSPESGLS---
PSTSRADAFLGALWGLSPKVMVVAEQEA-----------SH-N-AAGLTERFV-------EALNYYAALFDCL-EV-G----AARGSVE------
RARVERWLLGEEIKNIV-AC----DGG--E-----R----RE-RHERLER----WARRLEG-AGFGRVPLSYYALLQARRVAQ--------
GLGCDGFKVREE--------KG----------NFFLCWQDRALFSVSAW
>AtSCL3 .
LYLIHLLLTCANHVASGS-----LQ-NANAALEQ---L-SH----LAS-----PDGD--------T-MQRIAAYFTEA-LANRILK-SWP--GL---
YKALNA---TQTRTNNVSEEIHV-RRLFFEMFPILKVSYLLTNRAILEAME-------
GEKM--------------VHVIDLD-ASEP-----AQWLALLQAFNSRP--------EGP------P--------------HLRITGV------------
---------HHQ-KEVLEQMAHRLIEEAEKLDIP-FQFNP-VVS-RLD----------------------------CL-NV-------E-Q-----LRV-
-KTG-----
EALAVSSVLQLHTFLASDDDLMRKNCALRFQNNPSGVDLQRVLMMSHGSAAEARENDMSNNNGYSPSGDSASSLPLPSSGRTDSFLNAIWGLSPKVMVVT
EQDS-----------DH-N-GSTLMERLL-------ESLYTYAALFDCL-ET-K----VPRTSQD------RIKVEKMLFGEEIKNII-SC----EGF--
E-----R----RE-RHEKLEK----WSQRIDL-AGFGNVPLSYYAMLQARRLLQ--------GCGFDGYRIKEE--------SG----------
CAVICWQDRPLYSVSAW
>OsGRAS-26 .
VLMVSFLRSIAAFLADGT-----CQMQVNDGLSC---V-VD----LAG-----GDADGGGVGEGRS-AQRLASAFAEA-LALRFIL-PCD--GV---
CRSLHL-------------------------TRAPPPP----------------------AVSAA-RQGFRAMCPFVRLAAAAANLSIAEVME-------

AERA-------------VVHVVDLGGGVDA-----NQWVELVRLVAARP--------GGP------PG-------------LLRLTVV------------
---------NES-EDFLSAVAAYVAAEAQRLDLS-LQFHP-VLS-SIE----------------------------EL-SATATGSIGS-R-----LVV-
-IPG-----QPLAVVANLQIHRLLAFPDY----------------VD---------GVAS------RRPAAEQSGSSQHTMTT--
ATKTKADALLRAIRDLNPKLVVLTENEA-----------DH-N-VAELGARVW-------NALNYYAALFDAL-EA-SSTPPAAVPPHE------
RACVERWVLGEEIKDIV-VR----EGTG-------R----RE-RHETLGR----WAERMVA-AGFSPVTAA-
RALASTETLAQQMVAAGGGGAGAGVLRAAHG--------GGC---------FPVICWCDVPVFSVSTW
>OsGRAS-12 .
GDLKQVIAACGKAVDENS--------WYRDLLISE--L-RN----MVS-----ISGE--------P-MQRLGAYMLEG-LVARLSS-TGH--AL---
YKSLKC---KEPTSFELMSY-MHLLYEICPFFKFGYMSANGAIAEAVK-------
GENF--------------VHIIDFQ-IAQG-----SQWATMIQALAARP--------GGP------P--------------YLRITGI------------
DDSNS----AHARGGGLDIVGRRLFNIAQSCGLP-FEFNA-VPA-ASH----------------------------EVMLE-------H-------LDI-
-RSG-----EVIVVNFAYQLHHTP---DESVG----
IENHRDRILRMVKGLSPRVVTLVEQEA-----------NT-N-TAPFFNRYL-------ETLDYYTAMFEAI-DV-A----CPRDDKK------
RISTEQHCVARDIVNLI-AC----EGA--E-----R----VE-RHEPFGK----WRARLSM-AGFRPYPLSALVNNTIKKLLDSYHSY---------
YKLEER--------DG----------ALYLGWKNRKLVVSSAW
>AtSCL13 .
LDLKEVLVEAARAVADGD-----FA-TAYGFLDV---L-EQ----MVS-----VSGS--------P-IQRLGTYMAEG-LRARLEG-SGS--NI---
YKSLKC---NEPTGRELMSY-MSVLYEICPYWKFAYTTANVEILEAIA-------
GETR--------------VHIIDFQ-IAQG-----SQYMFLIQELAKRP--------GGP------P--------------LLRVTGV------------
DDSQS----TYARGGGLSLVGERLATLAQSCGVP-FEFHDAIMS-------------------------------GCKVQRE-------H-------LGL-
-EPG-----FAVVVNFPYVLHHMP---DESVS----
VENHRDRLLHLIKSLSPKLVTLVEQES-----------NT-N-TSPFLSRFV-------ETLDYYTAMFESI-DA-A----RPRDDKQ------
RISAEQHCVARDIVNMI-AC----EES--E-----R----VE-RHEVLGI----WRVRMMM-AGFTGWPVSTSAAFAASEMLKAYDKN---------
YKLGGH--------EG----------ALYLFWKRRPMATCSVW
>OsGRAS-2 .
VDLETLLIHCAQSVATDD-----RR-SATELLKQ---I-RQ----HAH-----ANGD--------G-DQRLAHCFANG-LEARLAG-TGS--QI---
YKNYTI---TRLPCTDVLKA-YQLYLAACPFKKISHYFANQTILNAVE-------
KAKK--------------VHIVDYG-IYYG-----FQWPCLIQRLSNRP--------GGP------P--------------KLRITGI------------
DTPQP----GFRPAERTEETGRYLSDYAQTFNVP-FEFQA-IAS-RFE----------------------------AVRME-------D-------LHI-
-EED-----EVLIVNCMFKFKNLM---DESVVAE----
SPRNMALKTIRKMNPHVFIHGVVNG-----------SY-N-APFFVTRFR-------EALFHYSAIFDML-ET-N----IPKDNEQ------
RLLIESALFSREAINVI-SC----EGL--E-----R----ME-RPETYKQ----WQVRNQR-VGFKQLPLNQDMMKRAREKVR-CYHKD--------
FII-------DED-NR----------WLLQGWKGRILFALSTW
>OsGRAS-46 .
VDLHTLLIHCAQAVATSD-----RR-SATELLKQ---I-KQ----NSS-----ARGD--------A-TQRLACCFAEG-LEARLAG-TGS--QV---
YKSLVA---KCTSTVDFLKA-YKLFAAACCIKKVSFIFSNKTILDAVA-------
GKRK--------------LHIVDYG-LSYG-----FQWPGLFKCLSERE--------GGP------P--------------EVRITGI------------
DFPQP----GFRPADQIEETGRRLSNCARQFGVP-FRFQA-IAA-KWE----------------------------TVRRE-------D-------
LHLDREEEEEEEEVLVVNCLHFLNALQ---DESVVVD--
SPSPRDMVLNNIRDMRPHVFVQCVVNG-----------AY-G-APFFLTRFR-------ETLFFYSSQFDML-DA-T----IPRDNDE------
RLLIERDILGRWALNVI-AC----EGA--D-----R----VD-RPETYKQ----WLVRNHR-AGLTQLPLQPQVVELVRDKVKKLYHKD--------
FVI-------DVD-HN----------WLLQGWKGRILYAMSTW
>OsGRAS-48 .
VDLHNLLLHCAQAVATDD-----RR-SAHELLRQ---I-KQ----HSS-----AWGD--------A-GQRLAHCFAQG-LEARLAG-TGS--QV---
YQSLMS---QRTSVVDFLKA-YRLYMEACCCKKVAFVFSNKTIYDAVA-------
GRRK--------------LHIVDYG-LSYG-----FQWPGLLRELAARR--------GGP------P--------------EVRITGI------------
DLPQP----GFRPDQHIEETGRRLSRYADELGVP-FKFHG-IAATKKE----------------------------SVRRE-------E-------LGE-
AEED-----EVVVVISLCHFRNVM---DESLQED-
SSRSPRDEVLGNIRRMRPDVFIHGIMNG-----------AY-G-ATYFLTRFR-------EALYYYAAQFDLL-DA-T----VGRESHE------
RMLVERDIFGRAALNVI-AC----EGA--E-----R----VE-RPEMYKQ----WQARNQR-AGLRQLPLNPQVVRLVLDKVRDKYHKD--------
FVV-------DED-QR----------WLLHRWKGRVLYALSTW
>AtGRAS-1 .
VDFRTLLTLCAQSVSAGDKI------TADDLLRQ---I-RK----QCS-----PVGD--------A-SQRLAHFFANA-LEARLEGSTGT--
MIQSYYDSISS---KKRTAAQILKS-YSVFLSASPFMTLIYFFSNKMILDAAK--
-----DASV--------------LHIVDFG-ILYG-----FQWPMFIQHLSKSN------------------PGLR-----------KLRITGI-------
-----EIPQH----GLRPTERIQDTGRRLTEYCKRFGVP-FEYNA-IASKNWE----------------------------TIKME-------E------
-FKI--RPN-----EVLAVNAVLRFKNLR---DVI------------PG---------------------------------------
EEDCPRDGFLKLIRDMNPNVFLSSTVNG-----------SF-N-APFFTTRFK-------EALFHYSALFDLF-GA-T----LSKENPE------
RIHFEGEFYGREVMNVI-AC----EGV--D-----R----VE-RPETYKQ----WQVRMIR-AGFKQKPVEAELVQLFREKMKKWGYHKD-------
FVL-------DED-SN----------WFLQGWKGRILFSSSCW
>AtSHR .
KWADSVLLEAARAFSDKD-----TA-RAQQILWT---L-NE----LSS-----PYGD--------T-EQKLASYFLQA-LFNRMTG-SGER-----
CYRTMVTAAA-T---EKTCSFESTRKT-VLKFQEVSPWATFGHVAANGAILEAVD------
-GEAK--------------IHIVDIS-----S-TFCTQWPTLLEALATRSD--------------DTP--------------HLRLTTVVVA-----
NKFVND-------QTASHRMMKEIGNRMEKFARLMGVP-FKFNI-IHH------------VGDLSEF-------------DL-NE---------------
LDV--KPD-----EVLAINCVGAMHGIA---
SRGSPRDAVISSFRRLRPRIVTVVEEEADLVGE------EEGGFDDEFLRGFG-------ECLRWFRVCFESW-EE-S----FPRTSNE------
RLMLERA-AGRAIVDLV-AC----EPS--D-----S----TE-RRETARK----WSRRMRN-SGFGAVGYSDEVADDVRALLRRYKEGV--------
WSMVQCP---DAA-------------GIFLCWRDQPVVWASAW
>OsGRAS-15 .
RWAAQLLMECARAVAGRD-----SQ-RVQQLMWM---L-NE----LAS-----PYGD---V------DQKLASYFLQG-LFARLTT-SGPR-----
TLRTLATASD---RNASFDSTRRT-ALKFQELSPWTPFGHVAANGAILESFLEA--
AAAGAAASSSSSSSSSTPPTRLHILDLS------NTFCTQWPTLLEALATRSSD-------------DTP--------------HLSITTVV--------
-----PTAAP--SAAAQRVMREIGQRLEKFARLMGVP-FSFRA-VHH------------SGDLADL-------------DL----------A-A-----
LDL--REGG--ATAALAVNCVNALRGVA---
RGRDAFVASLRRLEPRVVTVVEEEADLAAPEAD-ASSEADTDAAFVKVFG-------EGLRFFSAYMDSL-EE-S----FPKTSNE------RLSLERA-
VGRAIVDLV-SC----PAS--Q-----S----AE-RRETAAS----WARRMRS-AGFSPAAFSEDVADDVRSLLRRYKEG---------
WSMRDAGGATDDA-AG------AAAAGAFLAWKEQPVVWASAW

```
>AtGRAS-14 .
SVIIEQLFNAAELIGTTGNNNGDHTVLAQGILAR---L-NHH-LNTSS-----NHKS--------P-FQRAASHIAEA-LLSLIHN--------------
---------------------------ESSPPLITPENLILR----------------IAA-YRSFSETSPFLQFVNFTANQSILESCNES-----
GFDR--------------IHIIDFD-VGYG-----GQWSSLMQELASGV--------GGRRR--NRAS-------------SLKLTVF------------
APPPS----TVSDEFELRFTEENLKTFAGEVKIP-FEIEL-LSV---------------------------------EL--------LLNPAY--WPLSL-
-RSSE---KEAIAVNLPV-------------------------------------------------------------NSVAS------
GYLPLILRFLKQLSPNIVVCSDRGC-----------DR-N-DAPFPNAVI-------HSLQYHTSLLESL-DA-N----QNQDDSS---------
IERFWVQPSIEKLL-------------M----K-------RHRWIERS-PPWRILFTQ-CGFSPASLSQMAEAQAECLLQR------N--
PVRGFHVEKR--------QS----------SLVMCWQRKELVTVSAW
>AtSCL15 .
FDFIEDLIRVVDCVESDE-----LQ-LAQVVLSR---L-NQ---RLRS-----PAGR--------P-LQRAAFYFKEA-LGSFLTG--------------
---------------------------SNRNPIRLSSW--------------SEIVQRIRA-IKEYSGISPIPLFSHFTANQAILDSLS-------
SQSSS---------PF--VHVVDFE-IGFG-----GQYASLMREITEKSV-------SGG---------------------FLRVTAVV-----------
---------AEECAVETRLVKENLTQFAAEMKIR-FQIEF-VLMKTFE-----------------------------ML----------SFK----AIRF-
-VEG-----ERTVVLISPAIFRRL------------------------------------------------------------------
SGITDFVNNLRRVSPKVVVFVDSEG---------WTEIAG-SGSFRREFV-------SALEFYTMVLESL-DA-A----APPGDLV------
KKIVEAFVLRPKISAAV-------ETAA-D-----R-------RHTGEMT----WREAFCA-AGMRPIQLSQFADFQAECLLEKAQ--------
VRGFHVAKR--------QG----------ELVLCWHGRALVATSAW
>OsGRAS-45 .
DSLSDLLLAGAEAVEAGDSI------LASVAFSR---L-DD----FLS--GIPENGA-------ASSFDRLAYHFDQG-LRSRMSS-AST---------
---------------------------GCYQPEPLPSGNMLV--------------------HQIIQELSPFVKFAHFTTNQAILDAII-------
GDMD-------------VHVVDLN-IGEG-----IQWSSLMSDLAR---------CGGK---------------------SFRLTAITTY---------
---------ADCHASTHDTVVRLLSEFADSLELP-FQYNS-ICV-HNED---------------------------EL-HAFFEDCKGS-------VIV-
-SCD----TTSMYYKSLSTLQSLL------------------------------------------------------------------------
LVCVKKLQPKLVVTIEEDL--VRIGRG--VSP-S-SASFVEFFF-------EALHHFTTVFESM----ASC--FIGSSYE----
PCLRLVEMELLGPRIQDFV-----VKYGSV---------------RVEANASEVL---------EGFMACELSACNIAQARMLVGLFN---------
RVFGVVCE--------KG----------RLALCWKSRPLISVSVW
>OsGRAS-8 .
QQLLDELAAAAKATEAGNSV------GAREILAR---L-NQ----QLP-----PLGK--------P-FLRSASYLREA-LLLALAD-SHH--GV------
---------------------------SSVTTP--------------------LDVALKLAA-YKSFSDLSPVLQFANFTATQALLDEIG-------
GTAT------------SCIHVIDFD-LGVG-----GQWASFLQELAHRRAA------GGVTL----P--------------LLKLTAFVST---------
---------ASHHPLELHLTQDNLSQFAADLGIP-FEFNA-VSLDAFN--------PG------------------EL---I------S-----------
-STGD----EVVAVSLPVGCSARA----------------------------------------------------------------
PPLPAILRLVKQLSPKIVVAIDHGA-----------DR-A-DLSFSQHFL-------NCFQSCVFLLDSL-DA-AG---IDADSAC--------
KIERFLIQPRVHDMV----------L-G-------------RHKVHKA--IAWRSVFAA-AGFKPVPPSNLAEAQADCLLKRVQ--VR------
GFHVEK---------CG---------AALTLYWQRGELVSISSW
>OsGRAS-14 .
RWAEQLLNPCAVAVEAGN-----LS-RVQHLFYV---L-GE----LES-----FSGD--------A-NHRLAAHGLRA-LARWLPA------AVGP----
---AAA-AAV--------------RVPPCSERPTTAFAA--------------AEPRLFRAS-LIRFHEVSPWFALPNALANAAIAQASTCG--
AAGATPR-------------PLHVVDLG-VSHG-----VQWPTLLESL-TRQP-------GGRA----PP--------------SVRLTVVG--P-
GATATSPVAPFSASPPGYD-------FSPHLLRYAKSINLD-LRIS--------R--------AA------------------TL-DD-------A----
--VPG---DDG-----EALVVCLQFRLGHAA----------------------------------------------------AEE----------
RREVLRKARGLNPELVVL--SEL-----------DS-G-VGVVGGDGG-------SAAGEFAARLELL-WR-F----LESTSAAFKGKDV---
EERRLLEAEAGAILAAADVAAAGE--G-----R------------EG----WRERMAA-AGFEEAPFGAEAVESARSLLRKYDSG---------
WEMSAP----SPA-AA----------AVALRWKGQPVSFCSLW
>AtGRAS-16 .
RWAEKLLNPCALAITASN-----SS-RVQHYLCV---L-SE----LAS-----SSGD--------A-NRRLAAFGLRA-LQHHLSS----S-SVS-----
---------------------------SSFWPVFTFAS--------------AEVKMFQKT-LLKFYEVSPWFALPNNMANSAILQILAQDP----
KDKK------------D-LHIIDIG-VSHG-----MQWPTLLEALSCRL--------EGPP-----P--------------RVRITVISDL-------
TADIPFSVGPPGYN-------YGSQLLGFARSLKIN-LQIS--VLD--------------KL----------------QL------------------
IDT--SPH-----ENLIVCAQFRLHHLK--------------------------------------------------------H--
SINDERGETLKAVRSLRPKGVVLCENNGE---------CSS---SADFAAGFS-------KKLEYVWKFLDST-SS-G----FKEENSE----------
ERKLMEGEATKVLMNAGDMNEGK--E-----K------------------WYERMRE-AGFFVEAFEEDAVDGAKSLLRKYDNN---------WEIRME-
-------DG--------DTFAGLMWKGEAVSFCSLW
>OsGRAS-22 .
TTSRQLLSEAAAAIADGH-----NE-TAATHLTA---L-KR----AAN-----SRGD---V------EQRLVAMMVAA-LSSRIGQ-TASVPDICG----
----------------------------------------------------GETRAG-SQLLHDISPCFRLALHAANVAIVDAV--------
GDHRA-------------IHLVDFD-VSAP------QHADLIRCLAARRL----------------PGT------------SLKVTAV------------
TDPASPF--TQSVTATLH-----LQKLAERAGID-YRFKM-VSC-----------RAG------------------EI-EA-------S-K-----LGC-
-EAG-----EALAVNLAFALSHVP---------------------------------------------------------DESVS----
PANPRDEILRRVRALGPQVVALVEQEL-----------NS-N-TAPLTTRFT-------DACAHYGAILESL-DA-T----IPRESAE------
RARAEAA-LGGRAANAV-AR----EGA--D-----R----LE-RCEVFGK----WRSRFGM-AGFRPVALGPGIADQVLARQGPVAAG---------
FAVKAE--------NG----------VLRLGWMGRVVTVASAW
>AtSCL8 .
VCSRQTVMEIATAIAEGK-----TE-IATEILAR---V-SQ----TPNL----ERNS----------EEKLVDFMVAA-LRSRIAS------PV------
-------------------------------------------------TELYGKEHLIS-TQLLYELSPCFKLGFEAANLAILDAADNND----
GGMMI-------------PHVIDFD-IGEG-----GQYVNLLRTLSTRR--------NGKSQSQNSP--------------VVKITAVANNVYGCLV---
---------DDGGEERLKAVGDLLSQLGDRLGIS-VSFNV-VTSLRLG----------------------------DLNRE-------S-------LGC-
-DPD-----ETLAVNLAFKLYRVP---------------------------------------------------------DESVC----
TENPRDELLRRVKGLKPRVVTLVEQEM-----------NS-N-TAPFLGRVS-------ESCACYGALLESV-ES-T----VPSTNSD------
RAKVEEG-IGRKLVNAV-AC----EGI--D-----R----IE-RCEVFGK----WRMRMSM-AGFELMPLSEKIAESMKSRGNRVHPG---------
FTVKED--------NG----------GVCFGWMGRALTVASAW
```

The entire GRAS sequence was used for the alignment while the sequences upstream and downstream of the conserved LHRI and SAW domains, respectively, have been removed.

Figure S5. Phylogenetic reconstruction revealed the absent of ERAMOSA in legumes.

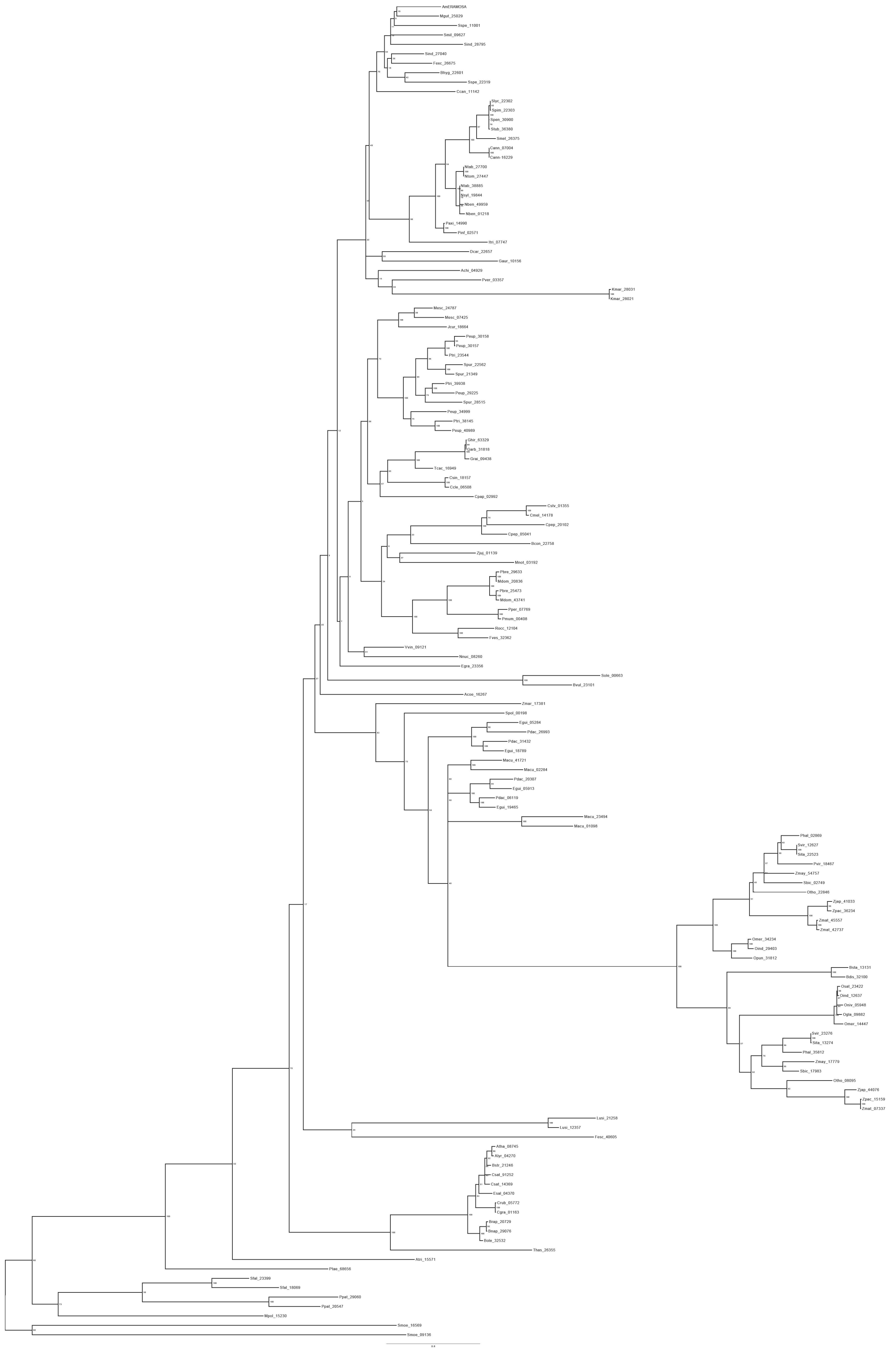

Phylogenetic tree containing all available ERAMOSA orthologues verifies that ERA cannot be found in the Leguminosae family. In order to avoid an ungainly tree, genes similar to ERA have not been included in the analysis. The phylogenetic tree has been constructed using PhyML software; the scale bar indicates number of substitutions per site.

Table S1. List of primers used in this study.

**Primers**		**Sequence**
Degenerate primers	OL135-ERAf	5'-CARTGGCCNCCNYTNATGCA-3'
	OL136-ERAr	5'-TGRTTYTGCCANCCNARRAA-3'
Genotyping	Am1-f	5'-ACAAGCCATCCACATACTCG-3'
	Am2-WTr	5'-AACACCGCCGCGTAATGATC-3'
	Am9-mutr	5'-TCTTGGGACATAGGTTTTATGCGACAGAT-3'
Semi-Q RT-PCR	Am7-ERAf	5'-TGAAGAAGCCCTACTTCAGTCC-3'
	Am12-ERAr	5'-TCGAGTATGTGGATGGCTTG-3'
	Ve21-ACTf	5'-GCCAAAGCAGTGATCTCTTTGCTC-3'
	Ve33-ACTr	5'-CACTCCTGCCATGTATGTCGCTAT-3'
In situ	Am1-ERAf	5'-ACAAGCCATCCACATACTCG-3'
	Am2-ERAr	5'-AACACCGCCGCGTAATGATC-3'
	Am5-ERAr	5'-taatacgactcactatagggAACACCGCCGCGTAATGATC-3'
	Am39-STMf	5'-TCTCAACAAGGGTTTTCAGAC-3'
	Am40-STMr	5'-GCTGCTTCTTCTAGCCTCGA-3'
	Am41-STMr	5'-taatacgactcactatagggGCTGCTTCTTCTAGCCTCGA-3'
Cloning	Am59-ERAf	5'-ggggacaagtttgtacaaaaaagcaggcttcATGTTGGGCAGCTCATTTGG-3'
	Am60-ERAr	5'-ggggaccactttgtacaagaaagctgggtTCAGTGCCACGAAGAAACCG-3'
	Am67-INCOf	5'-ggggacaagtttgtacaaaaaagcaggcttcATGGCTAGGGAGAAGATCCAGATC-3'
	Am68-INCOr	5'-ggggaccactttgtacaagaaagctgggtTTCAGTTTCAGCCAGAGTATGG-3'
	Am69-SQUAf	5'-ggggacaagtttgtacaaaaaagcaggcttcATGGGGAGAGGGAAAGTACAACTG-3'
	Am70-SQUAr	5'-ggggaccactttgtacaagaaagctgggtTCATGCAGCAAAGCATCCAAGATG-3'

Table S2. *Locus*/sequence ID of LATERAL SUPPRESSOR sequences used for the construction of the phylogenetic tree in Fig. 5.

	**Clade**	**Subfamily**	**Species**	**Locus/sequence ID**	**Database**	**References**
**Ppat Ls1**	*Ls*	*Ls*	*Physcomitrella patens*	Pp1s116_166V6	Phytozome	
**Ppat Ls2**	*Ls*	*Ls*	*Physcomitrella patens*	Pp1s240_118V6	Phytozome	
**Ppat SCL4**	*SCL4*	*Ls*	*Physcomitrella patens*	Pp1s362_33V6	Phytozome	
**Sm Ls1**	*Ls*	*Ls*	*Selaginella moellendorffii*	scaffold_17:651,163..652,533 (reverse orientation)	Phytozome	
**Sm Ls2**	*Ls*	*Ls*	*Selaginella moellendorffii*	84560	Phytozome	
**Sm SCL4-1**	*SCL4*	*Ls*	*Selaginella moellendorffii*	scaffold_78:396,615..398,099	Phytozome	
**Sm SCL4-2**	*SCL4*	*Ls*	*Selaginella moellendorffii*	122435	Phytozome	
**Paqu *Ls***	*Ls*	*Ls*	*Pteridium aquilinum*	EST_lrc34288		[2]
**Pa Ls**	*Ls*	*Ls*	*Picea abies*	MA_10255g0010	Congenie.org	
**Pa SCL4**	*SCL4*	*Ls*	*Picea abies*	MA_10435790g0010	Congenie.org	
**Atri Ls**	*Ls*	*Ls*	*Amborella trichopoda*	AmTr_v1.0_scaffold00061.21	Amborella Genome Database	
**Atri SCL4**	*SCL4*	*Ls*	*Amborella trichopoda*	AmTr_v1.0_scaffold00059.32	Amborella Genome Database	
**Pd Ls1**	*Ls*	*Ls*	*Phoenyx dactylifera*	PDK_30s827291c009		[3]
**Pd Ls2**	*Ls*	*Ls*	*Phoenyx dactylifera*	PDK_30s722381c002		[3]
**Pd Ls3**	*Ls*	*Ls*	*Phoenyx dactylifera*	PDK_30s772531c007		[3]
**Ma Ls1**	*Ls*	*Ls*	*Musa acuminata*	GSMUA_Achr1G09360_001	Genoscope/Cirad	
**Ma Ls2**	*Ls*	*Ls*	*Musa acuminata*	GSMUA_Achr7G16880_001	Genoscope/Cirad	
**Ma Ls3**	*Ls*	*Ls*	*Musa acuminata*	GSMUA_Achr1G19470_001	Genoscope/Cirad	
**Ma Ls4**	*Ls*	*Ls*	*Musa acuminata*	GSMUA_AchrUn_randomG25630_001	Genoscope/Cirad	
**Ma SCL4-1**	*SCL4*	*Ls*	*Musa acuminata*	GSMUA_Achr3G30530_001	Genoscope/Cirad	
**Ma SCL4-2**	*SCL4*	*Ls*	*Musa acuminata*	GSMUA_AchrUn_randomG22160_001	Genoscope/Cirad	
**Ma SCL4-3**	*SCL4*	*Ls*	*Musa acuminata*	GSMUA_Achr4G07320_001	Genoscope/Cirad	
**Os MOC1**	*Ls*	*Ls*	*Oryza sativa*	LOC_Os06g40780	Phytozome	[4]
**OsGRAS-7**	*Ls*	*Ls*	*Oryza sativa*	LOC_Os02g10360	Phytozome	[5]
**OsGRAS-19**	*SCL4*	*Ls*	*Oryza sativa*	LOC_Os03g51330	Phytozome	[5]
**Ac Ls**	*Ls*	*Ls*	*Aquilegia coerulea*	Aquca_045_00170	Phytozome	

**Ac SCL4-1**	*SCL4*	*Ls*	*Aquilegia coerulea*	Aquca_010_00478	Phytozome	
**Ac SCL4-2**	*SCL4*	*Ls*	*Aquilegia coerulea*	Aquca_013_00373	Phytozome	
**Vv Ls**	*Ls*	*Ls*	*Vitis vinifera*	GSVIVG01014862001	Phytozome	
**Vv SCL4**	*SCL4*	*Ls*	*Vitis vinifera*	GSVIVG01018975001	Phytozome	
**Rc Ls**	*Ls*	*Ls*	*Ricinus communis*	28966.t000014	Phytozome	
**Rc SCL4**	*SCL4*	*Ls*	*Ricinus communis*	29634.t000102	Phytozome	
**Me Ls1**	*Ls*	*Ls*	*Manihot esculenta*	cassava4.1_022846m.g	Phytozome	
**Me Ls2**	*Ls*	*Ls*	*Manihot esculenta*	cassava4.1_022511m.g	Phytozome	
**Me SCL4-1**	*SCL4*	*Ls*	*Manihot esculenta*	cassava4.1_003937m.g	Phytozome	
**Me SCL4-2**	*SCL4*	*Ls*	*Manihot esculenta*	cassava4.1_003952m.g	Phytozome	
**Lu Ls1**	*Ls*	*Ls*	*Linum usitatissimum*	Lus10032882.g	Phytozome	
**Lu Ls2**	*Ls*	*Ls*	*Linum usitatissimum*	Lus10027123.g	Phytozome	
**Lu SCL4-1**	*SCL4*	*Ls*	*Linum usitatissimum*	Lus10041740.g	Phytozome	
**Lu SCL4-2**	*SCL4*	*Ls*	*Linum usitatissimum*	Lus10024014.g	Phytozome	
**Pt Ls1**	*Ls*	*Ls*	*Populus trichocarpa*	Potri.001G473200	Phytozome	
**Pt Ls2**	*Ls*	*Ls*	*Populus trichocarpa*	Potri.015G008300	Phytozome	
**Pt Ls3**	*Ls*	*Ls*	*Populus trichocarpa*	Potri.012G020200	Phytozome	
**Pt SCL4-1**	*SCL4*	*Ls*	*Populus trichocarpa*	Potri.007G026300	Phytozome	
**Pt SCL4-2**	*SCL4*	*Ls*	*Populus trichocarpa*	Potri.005G123800	Phytozome	
**Mt SCL4**	*SCL4*	*Ls*	*Medicago truncatula*	Medtr4g077760 and Medtr4g077750	Phytozome	
**Pv SCL4**	*SCL4*	*Ls*	*Phaseolus vulgaris*	Phvul.011G020600	Phytozome	
**Gm SCL4-1**	*SCL4*	*Ls*	*Glycine max*	Glyma12g02060	Phytozome	
**Gm SCL4-2**	*SCL4*	*Ls*	*Glycine max*	Glyma11g09761	Phytozome	
**Cs Ls**	*Ls*	*Ls*	*Cucumis sativus*	Cucsa.041490	Phytozome	
**Cs SCL4-1**	*SCL4*	*Ls*	*Cucumis sativus*	Cucsa.201400	Phytozome	
**Cs SCL4-2**	*SCL4*	*Ls*	*Cucumis sativus*	Cucsa.127390	Phytozome	
**Pp Ls**	*Ls*	*Ls*	*Prunus persica*	ppa025834m.g	Phytozome	
**Pp SCL4**	*SCL4*	*Ls*	*Prunus persica*	ppa003124m.g	Phytozome	
**Fv Ls**	*Ls*	*Ls*	*Fragaria vesca*	gene01184-v1.0-hybrid	Phytozome	
**Fv SCL4**	*SCL4*	*Ls*	*Fragaria vesca*	gene14780-v1.0-hybrid	Phytozome	

**Md Ls1**	*Ls*	*Ls*	*Malus domestica*	MDP0000159341	Phytozome	
**Md Ls2**	*Ls*	*Ls*	*Malus domestica*	MDP0000389025	Phytozome	
**Md SCL4-1**	*SCL4*	*Ls*	*Malus domestica*	MDP0000256486	Phytozome	
**Md SCL4-2**	*SCL4*	*Ls*	*Malus domestica*	MDP0000575908	Phytozome	
**At LAS**	*Ls*	*Ls*	*Arabidopsis thaliana*	AT1G55580	Phytozome	[6]
**At SCL4 (AtGRAS-32)**	*SCL4*	*Ls*	*Arabidopsis thaliana*	AT5G66770	Phytozome	[5,7]
**At SCL7 (AtGRAS-19)**	*SCL4*	*Ls*	*Arabidopsis thaliana*	AT3G50650	Phytozome	[5,7]
**Cr Ls**	*Ls*	*Ls*	*Capsella rubella*	Carubv10011270m.g	Phytozome	
**Cr SCL4-1**	*SCL4*	*Ls*	*Capsella rubella*	Carubv10016973m.g	Phytozome	
**Cr SCL4-2**	*SCL4*	*Ls*	*Capsella rubella*	Carubv10017026m.g	Phytozome	
**Es Ls**	*Ls*	*Ls*	*Eutrema salsugineum*	Thhalv10011480m.g	Phytozome	
**Es SCL4-1**	*SCL4*	*Ls*	*Eutrema salsugineum*	Thhalv10003860m.g	Phytozome	
**Es SCL4-2**	*SCL4*	*Ls*	*Eutrema salsugineum*	Thhalv10011122m.g	Phytozome	
**Cp Ls**	*Ls*	*Ls*	*Carica papaya*	evm.TU.supercontig_12.48	Phytozome	
**Cp SCL4**	*SCL4*	*Ls*	*Carica papaya*	evm.TU.supercontig_6.108	Phytozome	
**Gr Ls**	*Ls*	*Ls*	*Gossypium raimondii*	Gorai.004G034900	Phytozome	
**Gr SCL4-1**	*SCL4*	*Ls*	*Gossypium raimondii*	Gorai.008G068100	Phytozome	
**Gr SCL4-2**	*SCL4*	*Ls*	*Gossypium raimondii*	Gorai.003G015400	Phytozome	
**Tc Ls**	*Ls*	*Ls*	*Theobroma cacao*	Thecc1EG030498	Phytozome	
**Tc SCL4**	*SCL4*	*Ls*	*Theobroma cacao*	Thecc1EG000399	Phytozome	
**Eg Ls**	*Ls*	*Ls*	*Eucalyptus grandis*	Eucgr.F01978	Phytozome	
**Eg SCL4**	*SCL4*	*Ls*	*Eucalyptus grandis*	Eucgr.I02451	Phytozome	
**Csi Ls**	*Ls*	*Ls*	*Citrus sinensis*	orange1.1g048299m.g	Phytozome	
**Csi SCL4**	*SCL4*	*Ls*	*Citrus sinensis*	orange1.1g006986m.g	Phytozome	
**Cc Ls**	*Ls*	*Ls*	*Citrus clementina*	Ciclev10008226m.g	Phytozome	
**Cc SCL4**	*SCL4*	*Ls*	*Citrus clementina*	Ciclev10025169m.g	Phytozome	
**Am ERA**	*Ls*	*Ls*	*Antirrhinum majus*		This work	
**Mg Ls**	*Ls*	*Ls*	*Mimulus guttatus*	mgv1a017979m.g	Phytozome	
**Mg SCL4**	*SCL4*	*Ls*	*Mimulus guttatus*	mgv1a018392m.g	Phytozome	

**Sl Ls**	*Ls*	*Ls*	*Solanum lycopersicum*	Solyc07g066250.1	Phytozome	[8]
**Sl SCL4**	*SCL4*	*Ls*	*Solanum lycopersicum*	Solyc02g085340.1	Phytozome	
**St Ls**	*Ls*	*Ls*	*Solanum tuberosum*	PGSC0003DMG400022244	Phytozome	
**St SCL4**	*SCL4*	*Ls*	*Solanum tuberosum*	PGSC0003DMG400014900	Phytozome	
**At SCL15 (AtGRAS-25)**		*HAM*	*Arabidopsis thaliana*	AT4G36710	Phytozome	
**AtGRAS-14**		*HAM*	*Arabidopsis thaliana*	AT2G45160	Phytozome	
**AtGRAS-23**		*HAM*	*Arabidopsis thaliana*	AT4G08250	Phytozome	
**OsGRAS-8**		*HAM*	*Oryza sativa*	LOC_Os02g44370	Phytozome	
**OsGRAS-45**		*HAM*	*Oryza sativa*	LOC_Os11g06180	Phytozome	
**OsGRAS-13**		*HAM*	*Oryza sativa*	LOC_Os03g15680	Phytozome	
**At SCR**		*SCR*	*Arabidopsis thaliana*	AT3G54220	Phytozome	
**AtGRAS-28**		*SCR*	*Arabidopsis thaliana*	AT5G41920	Phytozome	
**OsGRAS-28**		*SCR*	*Oryza sativa*	LOC_Os05g40710	Phytozome	
**OsGRAS-41**		*SCR*	*Oryza sativa*	LOC_Os11g03110	Phytozome	
**AtGRAS-8**			*Arabidopsis thaliana*	AT1G63100	Phytozome	
**OsGRAS-32**			*Oryza sativa*	LOC_Os06g03710	Phytozome	
**At RGA**		*DELLA*	*Arabidopsis thaliana*	AT2G01570	Phytozome	
**OsGRAS-1**		*DELLA*	*Oryza sativa*	LOC_Os01g45860	Phytozome	
**At SCL3 (AtGRAS-5)**		*SCL3*	*Arabidopsis thaliana*	AT1G50420	Phytozome	
**OsGRAS-6**		*SCL3*	*Oryza sativa*	LOC_Os01g71970	Phytozome	
**OsGRAS-26**		*SCL3*	*Oryza sativa*	LOC_Os05g31380	Phytozome	
**At SHR**		*SHR*	*Arabidopsis thaliana*	AT4G37650	Phytozome	
**AtGRAS-16**		*SHR*	*Arabidopsis thaliana*	AT3G13840	Phytozome	
**OsGRAS-14**		*SHR*	*Oryza sativa*	LOC_Os03g29480	Phytozome	
**OsGRAS-15**		*SHR*	*Oryza sativa*	LOC_Os03g31880	Phytozome	
**At SCL13 (AtGRAS-24)**		*PAT1*	*Arabidopsis thaliana*	AT4G17230	Phytozome	
**At SCL8 (AtGRAS-30)**		*PAT1*	*Arabidopsis thaliana*	AT5G52510	Phytozome	

**OsGRAS-12**		*PAT1*	*Oryza sativa*	LOC_Os03g09280	Phytozome	
**OsGRAS-22**		*PAT1*	*Oryza sativa*	LOC_Os04g49110	Phytozome	
**AtGRAS-1**		*LISCL*	*Arabidopsis thaliana*	AT1G07520	Phytozome	
**OsGRAS-2**		*LISCL*	*Oryza sativa*	LOC_Os01g62460	Phytozome	
**OsGRAS-46**		*LISCL*	*Oryza sativa*	LOC_Os11g47870	Phytozome	
**OsGRAS-48**		*LISCL*	*Oryza sativa*	LOC_Os11g47900	Phytozome	
**OsGRAS-4**			*Oryza sativa*	LOC_Os01g67650	Phytozome	
**OsGRAS-5**			*Oryza sativa*	LOC_Os01g67670 / LOC_Os01g71970	Phytozome	

SUPPLEMENTARY REFERENCES

1. Vincent, C. A. & Coen, E. S. A temporal and morphological framework for flower development in Antirrhinum majus. *Can. J. Bot.* **82,** 681–690 (2004).

2. Der, J. P., Barker, M. S., Wickett, N. J., dePamphilis, C. W. & Wolf, P. G. De novo characterization of the gametophyte transcriptome in bracken fern, Pteridium aquilinum. *BMC Genomics* **12,** 99 (2011).

3. Al-Dous, E. K. *et al.* De novo genome sequencing and comparative genomics of date palm (Phoenix dactylifera). *Nat. Biotechnol.* **29,** 521–527 (2011).

4. Li, X. *et al.* Control of tillering in rice. *Nature* **422,** 618–621 (2003).

5. Tian, C., Wan, P., Sun, S., Li, J. & Chen, M. Genome-wide analysis of the GRAS gene family in rice and Arabidopsis. *Plant Mol. Biol.* **54,** 519–532 (2004).

6. Greb, T. *et al.* Molecular analysis of the LATERAL SUPPRESSOR gene in Arabidopsis reveals a conserved control mechanism for axillary meristem formation. *Genes Dev.* **17,** 1175–1187 (2003).

7. Pysh, L. D., Wysocka-Diller, J. W., Camilleri, C., Bouchez, D. & Benfey, P. N. The GRAS gene family in Arabidopsis: sequence characterization and basic expression analysis of the SCARECROW-LIKE genes. *Plant J.* **18,** 111–119 (1999).

8. Schumacher, K., Schmitt, T., Rossberg, M., Schmitz, G. & Theres, K. The Lateral suppressor (Ls) gene of tomato encodes a new member of the VHIID protein family. *Proc. Natl. Acad. Sci. U. S. A.* **96,** 290–295 (1999).
